# The Acute Demands of Repeated-Sprint Training on Physiological, Neuromuscular, Perceptual and Performance Outcomes in Team Sport Athletes: A Systematic Review and Meta-analysis

**DOI:** 10.1007/s40279-023-01853-w

**Published:** 2023-05-24

**Authors:** Fraser Thurlow, Jonathon Weakley, Andrew D. Townshend, Ryan G. Timmins, Matthew Morrison, Shaun J. McLaren

**Affiliations:** 1 School of Behavioural and Health Sciences, Australian Catholic University, Brisbane, Australia; 2 Carnegie Applied Rugby Research (CARR) Centre, Carnegie School of Sport, Leeds Beckett University, Leeds, UK; 3 Sports Performance, Recovery, Injury and New Technologies (SPRINT) Research Centre, Australian Catholic University, Brisbane, Australia; 4 Newcastle Falcons Rugby Club, Newcastle Upon Tyne, UK; 5 Institute of Sport, Manchester Metropolitan University, Manchester, UK

## Abstract

**Background:**

Repeated-sprint training (RST) involves maximal-effort, short-duration sprints (≤ 10 s) interspersed with brief recovery periods (≤ 60 s). Knowledge about the acute demands of RST and the influence of programming variables has implications for training prescription.

**Objectives:**

To investigate the physiological, neuromuscular, perceptual and performance demands of RST, while also examining the moderating effects of programming variables (sprint modality, number of repetitions per set, sprint repetition distance, inter-repetition rest modality and inter-repetition rest duration) on these outcomes.

**Methods:**

The databases Pubmed, SPORTDiscus, MEDLINE and Scopus were searched for original research articles investigating overground running RST in team sport athletes ≥ 16 years. Eligible data were analysed using multi-level mixed effects meta-analysis, with meta-regression performed on outcomes with ~ 50 samples (10 per moderator) to examine the influence of programming factors. Effects were evaluated based on coverage of their confidence (compatibility) limits (CL) against elected thresholds of practical importance.

**Results:**

From 908 data samples nested within 176 studies eligible for meta-analysis, the pooled effects (± 90% CL) of RST were as follows: average heart rate (HR_avg_) of 163 ± 9 bpm, peak heart rate (HR_peak_) of 182 ± 3 bpm, average oxygen consumption of 42.4 ± 10.1 mL·kg^−1^·min^−1^, end-set blood lactate concentration (B[La]) of 10.7 ± 0.6 mmol·L^−1^, deciMax session ratings of perceived exertion (sRPE) of 6.5 ± 0.5 au, average sprint time (*S*_avg_) of 5.57 ± 0.26 s, best sprint time (*S*_best_) of 5.52 ± 0.27 s and percentage sprint decrement (*S*_dec_) of 5.0 ± 0.3%. When compared with a reference protocol of 6 × 30 m straight-line sprints with 20 s passive inter-repetition rest, shuttle-based sprints were associated with a substantial increase in repetition time (*S*_avg_: 1.42 ± 0.11 s, *S*_best_: 1.55 ± 0.13 s), whereas the effect on sRPE was trivial (0.6 ± 0.9 au). Performing two more repetitions per set had a trivial effect on HR_peak_ (0.8 ± 1.0 bpm), B[La] (0.3 ± 0.2 mmol·L^−1^), sRPE (0.2 ± 0.2 au), *S*_avg_ (0.01 ± 0.03) and *S*_dec_ (0.4; ± 0.2%). Sprinting 10 m further per repetition was associated with a substantial increase in B[La] (2.7; ± 0.7 mmol·L^−1^) and *S*_dec_ (1.7 ± 0.4%), whereas the effect on sRPE was trivial (0.7 ± 0.6). Resting for 10 s longer between repetitions was associated with a substantial reduction in B[La] (−1.1 ± 0.5 mmol·L^−1^), *S*_avg_ (−0.09 ± 0.06 s) and *S*_dec_ (−1.4 ± 0.4%), while the effects on HR_peak_ (−0.7 ± 1.8 bpm) and sRPE (−0.5 ± 0.5 au) were trivial. All other moderating effects were compatible with both trivial and substantial effects [i.e. equal coverage of the confidence interval (CI) across a trivial and a substantial region in only one direction], or inconclusive (i.e. the CI spanned across substantial and trivial regions in both positive and negative directions).

**Conclusions:**

The physiological, neuromuscular, perceptual and performance demands of RST are substantial, with some of these outcomes moderated by the manipulation of programming variables. To amplify physiological demands and performance decrement, longer sprint distances (> 30 m) and shorter, inter-repetition rest (≤ 20 s) are recommended. Alternatively, to mitigate fatigue and enhance acute sprint performance, shorter sprint distances (e.g. 15–25 m) with longer, passive inter-repetition rest (≥ 30 s) are recommended.

**Supplementary Information:**

The online version contains supplementary material available at 10.1007/s40279-023-01853-w.

## Key Points

**Table:** 

The most common RST set configuration is 6 × 30 m straight-line sprints with 20 s of passive inter-repetition rest.
The reference estimates for HR_avg_ (90% HR_max_), *V*O_2avg_ (~ 70–80% *V*O_2max_) and B[La] (10.8 mmol·L^−1^) demonstrate the substantial physiological demands of RST in team sport athletes. Associated prediction intervals for these estimates suggest that most of these demands are consistently substantial across many RST protocols, sports and athlete characteristics.
Shorter inter-repetition rest periods (≤ 20 s) and longer repetition distances (> 30 m) amplify physiological demands and cause greater inter-set reductions in sprint performance (i.e. performance fatigue). Inversely, longer inter-repetition rest periods (≥ 30 s) and shorter repetition distances (≤ 20 m) enhance acute sprint performance and reduce the physiological demands.
Shuttle-based protocols are associated with slower repetition times, likely due to the added change-of-direction component, but may reduce sprint decrement. The effect of shuttle versus straight-line RST protocols on physiological and perceptual outcomes remains inconclusive.
Performing two less repetitions per set (e.g. four as opposed to six repetitions) maintains the perceptual, performance and physiological demands of RST.
The findings from our investigation provide practitioners with the expected demands of RST and can be used to help optimise training prescription through the manipulation of programming variables.

## Introduction

Repeated-sprint training (RST) involves maximal-effort, short-duration sprints (≤ 10 s), interspersed with brief (≤ 60 s) recovery times [1]. It appears an effective and time-efficient training modality for physical adaptations in team-sport athletes, with as few as six sessions over two weeks shown to enhance high-speed running abilities [2]. The implementation of RST can also provide athletes with exposure to maximal sprinting, acceleration and deceleration, which are important components of team sport [3–5]. Throughout an athlete’s training program, there is a range of opportunities for RST to be used, such as during a pre-season where a progressive reduction in running volume and an increase in intensity is often implemented [6]. Alternatively, it could be employed during the playing season to promote the maintenance of specific physical qualities (e.g. speed, aerobic fitness), used as part of late-stage rehabilitation or implemented at a time when a training ‘shock-cycle’ is required. However, each training program requires different outcomes, with these attained through the manipulation of programming variables.

The type of stimulus is an important driver of the chronic adaptive response to training [7]. Repeated-sprint training is low-volume and short in duration, typically lasting 10–20 min per session, but due to the maximal intensity at which it is performed, it can generate adaptive events that ultimately result in the capacity for enhanced performance [8, 9]. This includes an improved aerobic and metabolic capacity [10–17]. However, there is considerable variation in RST prescription, with acute programming variables (e.g. sprint distance, rest duration, number of repetitions) regularly manipulated in research and practice [8, 18]. These changes can influence the internal and external load experienced by athletes during RST (i.e. the acute demands) and subsequently have the potential to cause diverse training adaptations [12]. For instance, in a study by Iaia et al. [19], higher within-set blood lactate concentration (~ 3 mmol⋅L^−1^ B[La]) was recorded during RST with shorter rest times (15 s versus 30 s), which can indicate a greater anaerobic contribution to exercise [20]. Accordingly, after six-weeks of training, the 15 s rest group achieved greater improvement in 200 m sprint time and the Yo-Yo intermittent recovery test level 2 compared with the 30 s group [19], with anaerobic energy production central to performance in these events [21, 22]. Thus, it is important to understand how the manipulation of programming variables affects the acute demands of RST, as this evidence can be useful to help explain how and why training adaptations may manifest.

There is conflicting evidence within and across studies regarding the effects of programming variables on the acute demands of RST. In a study by Alemdaroğlu et al. [23], B[La] and percentage sprint decrement (*S*_dec_) were greater with 6 × 40 m shuttle repeated-sprints compared with the same straight-line protocol. Conversely, compared with shuttle-based sprints, straight-line sprints induced greater demands when more repetitions were performed over a shorter distance (8 × 30 m repeated-sprints) [23]. The prescription of active inter-repetition rest has been shown to promote higher heart rate and oxygen consumption (*V*O_2_) compared with passive rest [24]. However, Keir et al. [25] found that demands were greater when passive rest, fewer repetitions, shorter rest time and a longer sprint distance were prescribed. Ultimately, there is an infinite combination of programming variables that can alter the training outcome, but the acute effects of these factors are not well understood. Therefore, to guide training prescription and enhance the effectiveness of RST, it is important to gain a quantitative understanding of the acute effects of each programming factor.

While excessive training loads can contribute to fatigue, an appropriate training dose may allow for greater improvements in fitness and performance [26]. Knowledge of the acute demands of RST can help practitioners manage fatigue and target specific training outcomes. Therefore, our systematic review and meta-analysis aims to (1) identify the most common RST set configuration, (2) evaluate and summarise the acute physiological, neuromuscular, perceptual and performance demands of RST, and (3) examine the meta-analytic effects of sprint modality, number of repetitions per set, sprint repetition distance, inter-repetition rest modality and inter-repetition rest duration on the acute RST demands.

## Methods

### Search Strategy

This study was conducted in accordance with the ‘Preferred Reporting Items for Systematic Reviews and Meta-analyses’ (PRISMA) guidelines [27] and registered on Open Science Framework (Registration 10.17605/OSF.IO/2XQ3A). A systematic search of the literature was conducted to find original research articles investigating the acute demands of RST in team sport athletes. The latest search was performed on 10 January 2022, using the electronic databases Pubmed, SPORTDiscus, MEDLINE and Scopus. No restrictions were imposed on the publication date. Relevant keywords for each search term were identified through pilot searching of titles/abstracts/full-texts of previously known articles. Key search terms were grouped and searched within the article title, abstract and keywords using the search phrase (‘repeat* sprint*’ OR ‘intermittent sprint*’ OR ‘multiple sprint*’) AND (‘exercise’ OR ‘ability’ OR ‘training’) AND (‘team sport’ OR ‘players’ OR ‘athletes’) AND (‘physiological’ OR ‘perceptual’ OR ‘neuromuscular’ OR ‘metabolic’ OR ‘fatigue’) NOT (‘cycling’ OR ‘swimming’). No medical subject headings were applied to the search phrase.

**Fig. 1:**
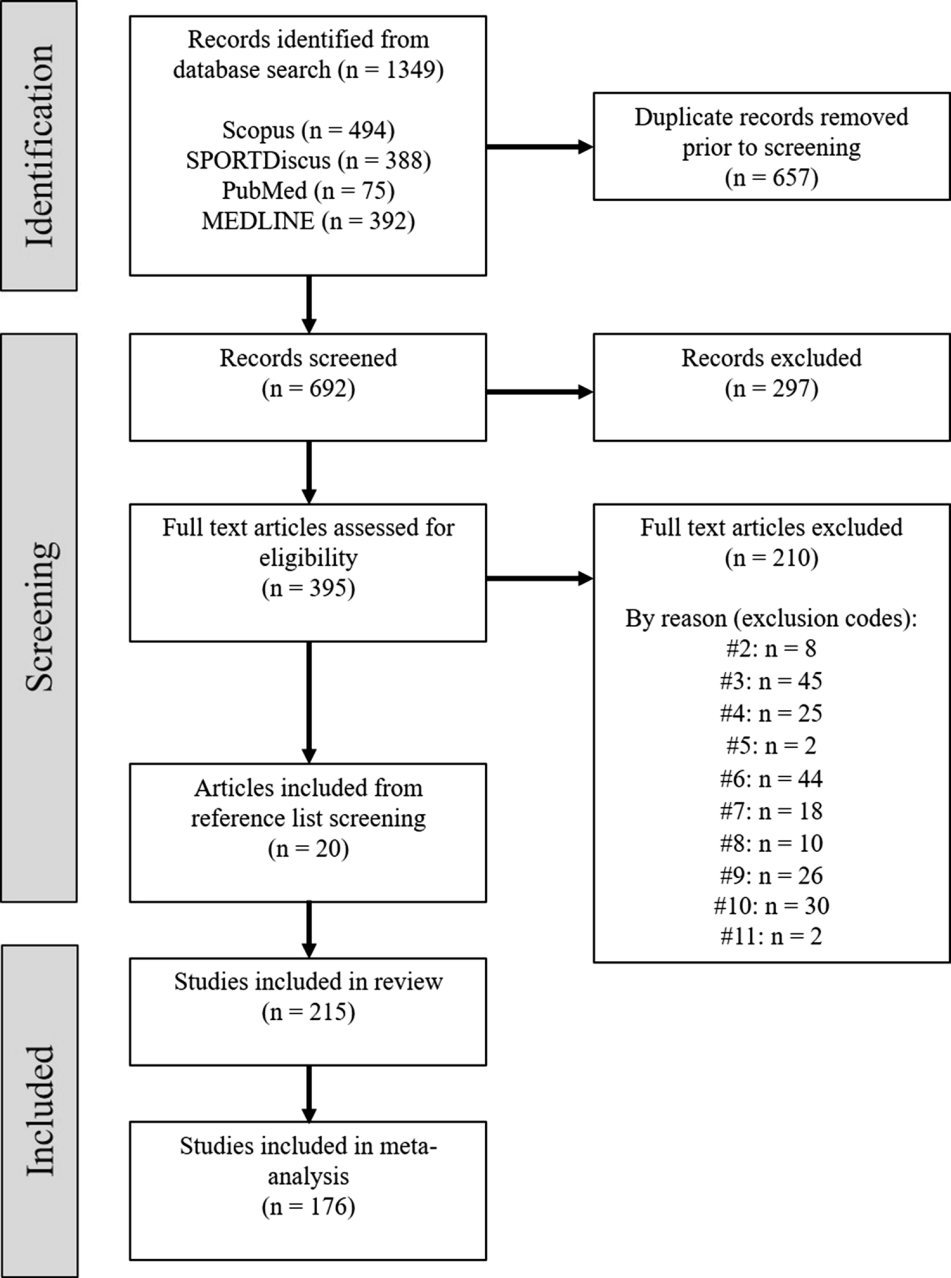
Flow diagram of the study selection process

Following the initial search of the literature, results were exported to EndNote library (Endnote X9, Clarivate Analytics, USA) and duplicates were removed. The remaining articles were then uploaded to Covidence (http://www.covidence.org, Melbourne, Australia), with the titles and abstracts independently screened by two authors (F.T., M.M.). Full-texts of the remaining articles were then accessed to determine their final inclusion–exclusion status. Articles selected for inclusion were agreed upon by both authors, with any disagreements resolved by discussion or a third author (J.W.). Furthermore, Google Scholar, as well as reference lists of all eligible articles and reviews [1, 8, 9, 28], were searched to retrieve any additional studies. Figure 1 displays the strategy for the study selection process used in this review.

### Inclusion–Exclusion Criteria

The inclusion and exclusion criteria can be found in Table 1. We chose to omit any studies in which the mean athlete age was ≤ 16 years, as children may respond differently to RST [29, 30]. Studies were excluded if RST was performed in ≥ 30 °C because larger performance decrements may occur in hot compared with cool conditions [31]. We acknowledge that the residual effects of intense exercise may last up to 72 h [32], but acute demands measured up to 24 h following RST was selected because: (a) it is common for RST and other team sport activity to be interspersed with minimal recovery time (i.e. < 72 h), (b) pilot scoping of the literature only identified five studies [33–37] that recorded measurements on athletes > 24 h. Several studies/protocols were excluded from this investigation that implemented repeated-sprint sequences with sport skill elements [38–42] or involved a reactive component in response to an external stimulus (e.g. light sensor) [43–46]. Evidence from studies involving both single-set and multi-set repeated sprints was recorded, including the acute demands from repeated-sprint ability tests. For studies that involved pre-post testing of RST, separated by an intervention period (e.g. training, supplementation), only the RST baseline results were reported to ensure that the intervention period did not bias the results. Where observational time-series studies measured RST across a season, results were included for each phase (e.g. pre-season, mid-season, post-season), providing that no intervention was implemented outside of usual practice.

**Table 1:** Study inclusion–exclusion criteria

Criteria	Inclusion	Exclusion
1	Original research article	Reviews, surveys, opinion pieces, books, periodicals, editorials, case studies, non-academic/non-peer-reviewed text
2	Full-text available in English	Cannot access the full text in English
3	Team sport athletes (field- or court-based invasion sports) of any gender	Non-team sports (e.g. solo, racquet or combat sports), ice-, sand- or water-based team sports, match officials, non-athletic populations. Studies that described participants as playing intermittent sports or used a combination of team sport and non-team sport athletes, unless group results were separated
4	Participants mean age ≥ 16 years. Where mean age was not provided, and if an age group was listed as U17 or above, this was accepted	Mean athlete age was < 16 years, or participants were described as U16 or below. Additionally, studies that used a combination of athletes below and above the age cut-off, unless group results were separated
5	Healthy, able-bodied, non-injured athletes	Special populations (e.g. clinical, patients), athletes with a physical or mental disability, or athletes considered to be injured or returning from injury
6	RST was over-ground running on a flat surface	RST was performed on a treadmill, cycle or another implement. RST was performed on a slope or sand
7	RST was performed at maximal intensity, with a mean work duration of ≤ 10 s or ≤ 80 m in distance, a recovery duration of ≤ 60 s and ≥ 2 repetitions performed in total. Single set and multi-set repeated-sprints	RST was performed at submaximal intensity, with a work duration of > 10 s or > 80 m, a recovery duration of > 60 s, and only a single sprint repetition
8	RST was a fixed protocol, without any sport skill elements	RST involved a reactive change of direction in response to an external stimulus (e.g. light sensor) or sport skill elements (e.g. passing, kicking, shooting)
9	Studies must have reported ≥ 1 acute outcome measure (outcome measures are presented in Sect. 2.3). Acute demands must have occurred during (within) or immediately following RST up to 24 h	No relevant outcome measures were reported. RST demands occurred > 24 h
10	≥ 1 condition or group must have performed the intervention under normal conditions (e.g. usual nutritional intake, hydrated state, normoxia, absence of ergogenic aids, ≤ 30 °C, regular warm-up protocol)	RST was performed in a possibly fatigued or potentiated state (e.g. sports training, maximal fitness assessment, pre-conditioning strategies) occurring within or 24 h before RST. Placebo treatments were used before or during RST
11	Sprint times were recorded using electronic timing gates	Sprint times were recorded with a hand-held stopwatch or a video-camera

*RST* repeated-sprint training, *U17* under 17 age group, *U18* under 18 age group

### Classification of Study Design

To provide information on study design (Supplementary Table S2), studies were categorised under four designs as follows: (1) observational – non-experimental, (2) single group pre-test post-test – experimental treatment applied to a single group of participants, with the dependent variable/s measured before and after treatment, (3) crossover – two or more experimental conditions applied to the same participants, with or without a control condition, (4) parallel groups – two or more experimental conditions applied to two groups of different participants, with or without a control condition. Additionally, single-group time-series designs were categorised under observational and denoted.

### Selection of Outcome Measures and Programming Variables

The outcome measures (Table 2) were selected on the basis of pilot scoping of the literature that identified commonly used indicators of internal responses to exercise and performance capacity in team sport settings [28, 47, 48]. Percentage sprint decrement, as defined by Fitzsimons et al. [49] and Glaister et al. [50], was chosen as it is the most ecologically valid index to quantify fatigue during RST [50]. However, caution should be taken when interpreting *S*_dec_ as weak relative and absolute reliability exists between repeated-sprint ability tests [51]. Blood lactate is sensitive to changes in exercise intensity and duration and is one of the preferred methods used to assess the anaerobic glycolytic contribution to exercise [20]. Sprint force–velocity–power parameters, as defined by Samozino et al. [52], and spring-mass model parameters, as defined by Morin et al. [53], were chosen as they represent field-based methods used to assess the mechanical effectiveness of sprinting and the neuromuscular manifestation of fatigue during over-ground running [54].

**Table 2:** Summary of the outcome measures of interest

Category	Measure	Metric
Physiological	HR	HR_avg_, HR_peak,_ HR_post_ and/or % HR_max_
	CK	CK 24 h
	B[La]	Post (0–10 min)
	*V*O_2_	*V*O_2avg_, *V*O_2peak_ and/or % *V*O_2max_
Neuromuscular	CMJ	JH
	Sprint FVP parameters as defined by Samozino et al. [43]	*V*_0_, *F*_0_, *P*_0_, RF_peak_, *D*_RF_
	SMM parameters as defined by Morin et al. [44]	*K*_vert_, *K*_leg_, Δ*L*, Δ*z*, *F*_z_max
Perceptual	sRPE	CR10^®^ and 6–20 sRPE scales [46]
Performance	Sprint times	*S*_best_, *S*_avg_, *S*_total_
	*S*_dec_	As defined by Fitzsimons [40] and Glaister et al. [41]

*sRPE* session ratings of perceived exertion, *CR10* Category-Ratio 10, *CMJ* counter movement jump, *JH* jump height, *FVP* force–velocity–power, *V*_*0*_ theoretical maximal velocity, *F*_*0*_ theoretical maximal force, *P*_*0*_ theoretical maximal power, *RF*_*peak*_ maximal ratio of force, *D*_*RF*_ slope/rate of decrease in ratio of force with increasing velocity, *SMM* spring-mass model, *K*_*vert*_ vertical stiffness, *K*_*leg*_ leg stiffness, *ΔL* leg compression, *Δz* centre of mass vertical displacement, *F*_*z*_*max* maximal vertical force, *HR* heart rate, *HR*_*avg*_ average heart rate, *HR*_*peak*_ peak heart rate, *HR*_*post*_ heart rate recorded immediately post exercise, *% HR*_*max*_ percentage of maximal heart rate, *CK* serum creatine kinase, *CK 24h* serum creatine kinase measured 24 h post exercise, *B*[*La*] blood lactate, *VO*_*2avg*_ average oxygen consumption, *% VO*_*2peak*_ percentage of peak oxygen consumption, *% VO*_*2max*_ percentage of max oxygen consumption, *S*_*best*_ best sprint time, *S*_*avg*_ average sprint time, *S*_*total*_ total sprint time, *S*_*dec*_ percentage sprint decrement

Programming variables recorded were: sprint modality (i.e. straight-line, 180° shuttle or multi-directional), number of repetitions per set, number of sets per session, sprint distance or duration per repetition, inter-repetition rest duration, inter-repetition rest modality, inter-set rest duration and inter-set rest modality.

### Extraction of Study Information

Mean and standard deviation data were extracted directly from tables and within the text of the included studies. To obtain data from studies where information was provided in figures, graph digitising software (WebPlotDigitizer, version 4.3, USA) was used. For studies where rest duration was given as an exercise to rest ratio or on a time cycle that included sprint time, an estimated ‘actual’ rest time was also established. This was determined by extracting average sprint time (*S*_avg_) data from studies, where provided. For example, if *S*_avg_ was 3.2 s and the recovery duration was given as 1:5 exercise to rest ratio, then the estimated recovery duration was 16 s, or if the recovery duration was given on a 30 s cycle, then the estimated recovery duration was 27 s, with recovery durations rounded to the nearest whole number.

With regards to sprint modality, shuttle repeated-sprints were defined as RST where one or more 180° changes of direction were performed. Multi-directional repeated-sprints involved RST where changes of direction were performed with angles other than 180°, but due to the large variety of designs (e.g. different angles and courses), this format was excluded from the meta-analysis. For rest modality, ‘passive’ included protocols where participants were required to walk back to a two-way start line (sprints alternating from both ends) in preparation for the next sprint. Where information relating to exercise protocols (e.g. sprint distance) could not be found within the study or clarification was required, authors were contacted. If authors did not respond, samples were removed from the meta-analysis. The Participant Classification Framework [55] was used to define training and performance calibre of the athletes included in our investigation (Supplementary Table S2).

Twenty-four estimates nested within 13 studies collected session ratings of perceived exertion (sRPE) via Borg’s 6–20 scale. For consistency with other included studies and to comply with more standard practice, 6–20 values were converted to Category–Ratio 10 (CR10^®^) units (deciMax) using the appropriate table conversion [56]. Standard deviations were converted by a factor that was proportionate to the mean value of each estimate, which ranged between 13–19 (conversion factors = 0.27–0.53). Where *V*O_2_ was expressed in absolute terms (L·min^−1^) [25], it was converted to relative terms (mL⋅min^−1^⋅kg^−1^) by extracting the mean body mass of the participants from the study. Where *S*_dec_ of 5% was set as the termination criteria [57], the mean number of repetitions was used for meta-analysis. Heart rates were inclusive of both the sprint component and inter-repetition rest periods, but samples were excluded [58] which continuously recorded heart rate during the inter-set rest periods. Due to a lack of studies reporting the effect of RST on peak heart rate (HR_peak_) as a percentage of maximal heart rate (HR_max_), this data was unable to be meta-analysed. However, these results [2, 59–62] are summarised in section 3.4.3. Post-exercise B[La] samples were meta-analysed together, irrespective of the exact time point that they were measured (i.e. 0–10 min). Although, for context, specific timepoints of each sample are given in Supplementary Table S3. Where studies provided multiple timepoints of B[La] collection, the highest value was used for meta-analysis. The considerable variation in measurement error between different jump systems makes it difficult to compare counter-movement jump (CMJ) height between different studies [63] and as such, CMJ height results were recorded, but not meta-analysed. For context, the type of jump measurement systems used in each study are noted alongside the results in Supplementary Table S3.

### Assessment of Reporting Quality and Risk of Bias

To assess the reporting quality and risk of bias within the studies included in this review, two authors (F.T. and M.M.) independently evaluated the literature using a modified version of the Downs and Black index. This scale includes 14 original items and ranks each item as 0 or 1, with higher total scores (out of 14) indicating higher quality studies. The original Downs and Black scale was reported to have acceptable test–retest (*r* = 0.88) and inter-rater reliability (*r* = 0.75) [64]. If there was an absence of clear information to assess an item on either scale, it was scored as 0. Any disagreements between the two authors were resolved by discussion or a third author (J.W.).

### Data Analysis

All analyses were performed in the statistical computing software R (Version 4.0.0; R Core Team, 2020). Studies eligible for meta-analysis often reported RST outcomes from several subgroups (e.g. elite versus non-elite, males versus females, etc.), from repeated measures taken on the same group of athletes (e.g. set 1 and set 2, warm-up A versus warm-up B, etc.), or a combination of both. To appropriately account for this hierarchical structure, in particular, the within-study correlation arising from repeated measures [65] and on the assumption that the true acute demand of RST varies between studies [66], data were analysed using multi-level mixed-effects meta-analysis via the *metafor* package [67]. Initial (baseline) models were run for each outcome measure with 10 or more estimates and fit using restricted maximum-likelihood. These models included only random effects, which were specified in a nested structure as studies (i.e. individual research papers; outer factor) and groups within studies (inner factor, [65]). Units of analysis were therefore individual estimates from groups within studies, given as the mean value of the outcome measure following RST. Both the associated standard deviation (SD) and sample size were used to calculate the variance of each estimate. When a study involved repeated measures (i.e. multiple rows of data for the same group of athletes), dependency was accounted for by replacing variance with the entire ‘V’ matrix; that is, the variance–covariance matrix of the estimates [65]. Block-diagonal covariance matrices were estimated with an assumed correlation of *r* = 0.5 using the *clubSandwich* package [68]. Since it is uncommon for studies to report the correlation coefficient between repeated measures [69], our assumption was informed by re-analysis of our previous (unpublished) work in team-sport RST.

Uncertainty in meta-analysed estimates was expressed using 90% compatibility (confidence) intervals (CI), calculated based on a *t*-distribution with denominator degrees of freedom given from the unique number of ‘group’ levels (i.e. the inner level of the random effects structure). Pooled estimates were also presented with 90% prediction intervals, which convey the likely range of the true demand of RST in similar future studies [70]. Between-study and between-group heterogeneity in each meta-analysed estimate was quantified as a SD [Sigma (*σ*)] [71]), with 90% CI calculated using the Q-profile method [72].

To examine the effect of programming variables on acute RST outcomes, candidate factors were added to the aforementioned baseline models as fixed effects for outcomes with sufficient estimates available (approximately 10 per moderator [73]). The five moderator variables were: sprint modality (categorical: straight-line or 180° shuttle), number of repetitions per set (continuous, linear), total distance covered in each repetition (continuous, linear), inter-repetition rest modality (categorical: active or passive) and inter-repetition rest duration (continuous, linear). Factors were re-scaled so that the reference (intercept) effect represented the performance or response to 6 m × 30 m straight-line sprints with 20 s passive rest between repetitions. The effects of each moderator were then estimated (along with 90% CI and 90% prediction intervals, where appropriate), with all other factors being held constant. Categorical moderators were given as the difference between levels (shuttle compared with straight-line sprints and active compared with passive inter-repetition rest). Continuous moderators were evaluated at a magnitude deemed to be practically relevant for training prescription: performing two more repetitions, sprinting 10 m further per repetition and resting for 10 s longer between repetitions. The effects of repetition distance on repetition time (average and fastest sprint) were not shown (but were still offset to a distance of 30 m), because the time taken to complete a sprint repetition is almost entirely dependent on the distance to be covered. The total amount of variance explained by the combination of moderators was given as a pseudo-*R*^2^ value, calculated by subtracting the total (pooled) variance from final models ($\sigma_{{{\text{mods}}}}^{2}$) as a fraction of baseline models ($\sigma_{{{\text{base}}}}^{2}$) from 1 (1 − $[\sigma_{{{\text{mods}}}}^{2} /\sigma_{{{\text{base}}}}^{2} ]$).

To provide an interpretation of programming moderators, we (subjectively) considered the entire range of the CI representative of values compatible with our models and assumptions [74], relying mostly on the point estimate. To further contextualise the practical relevance of moderators, we visually scaled effects against regions of practical significance. That is, reference values for each outcome measure that have been empirically or theoretically anchored to some real-world importance in the context of team-sport athletes and/or RST. These thresholds were: 2 bpm (~ 1%) in HR_peak_ [75], 1 au in CR10-scaled sRPE [76], a 1% faster or slower sprint time [77] based on the reference performance given as the intercept: 0.05 s for *S*_avg_, 0.04 s for best sprint time (*S*_best_) and 1% for *S*_dec_ across a set [77]. In absence of a recognised practical reference value for a change in B[La] above the anaerobic threshold, we used the value of a small, standardized effect. Between-athlete standard deviations from included estimates (*n* = 120) were meta-analysed on the log scale, as previously described (SD = 1.9 mmol·L^−1^, 90% CI 1.7–2.22), before being multiplied by 0.2. The threshold for a moderate standardised effect (0.6 × 1.9 mmol·L^−1^) was also calculated and shown for visual purposes. When a CI fell entirely inside the region of practical significance or predominantly inside one region, we declared an effect as trivial. When a CI fell entirely outside the region of practical significance or predominantly outside the region, we declared an effect substantial. If there was equal coverage of the CI across the trivial region and the substantial region in only one direction (i.e. positive or negative), the effect was deemed compatible with both trivial and substantial effects. Finally, when the CI spanned across substantial regions in both positive and negative directions, including the trivial region, an effect was deemed inconclusive.

## Results

Following the screening process (Fig. 1), 215 publications were included in our investigation, with data from 908 samples nested within 176 studies eligible for meta-analysis. Across all studies, there were 4818 athlete inclusions from 282 repeated-sprint protocols reported.

### Study Characteristics

The most common study design for investigations of acute demands of RST was single group, cross sectional observational (*n* = 87 studies, 40%). Soccer was the most investigated sport (*n* = 104, 48%), followed by basketball (*n* = 33, 15%), rugby (league, union and sevens) (*n* = 15, 7%), futsal (*n* = 14, 7%), handball (*n* = 12, 6%), field hockey (*n* = 10, 5%), Australian rules football (*n* = 5, 2%), volleyball (*n* = 3, 1%), netball (*n* = 2, 1%) and a mixture of team sports (*n* = 17, 8%). Of these sports, 21 (10%) studies involved elite/international level athletes, 125 (58%) studies involved highly trained/national level athletes and 58 (27%) studies involved trained/development level athletes, with 11 (5%) studies not reporting the training and performance calibre of the athletes. Female athletes were represented in 31 (14%) studies. A summary of the participants and study characteristics of included publications are provided in Supplementary Table S2.

### Outcomes for the Assessment of Reporting Quality and Risk of Bias

Supplementary Table S1 summarises the outcomes of the modified Downs and Black scale for the assessment of reporting quality and risk of bias. Results ranged from 7 to 12, with a mean score of 9.6 ± 0.9.

### Study Outcomes

A summary of the training protocols and study outcomes of included publications are provided in Supplementary Table S3.

**Fig. 2:**
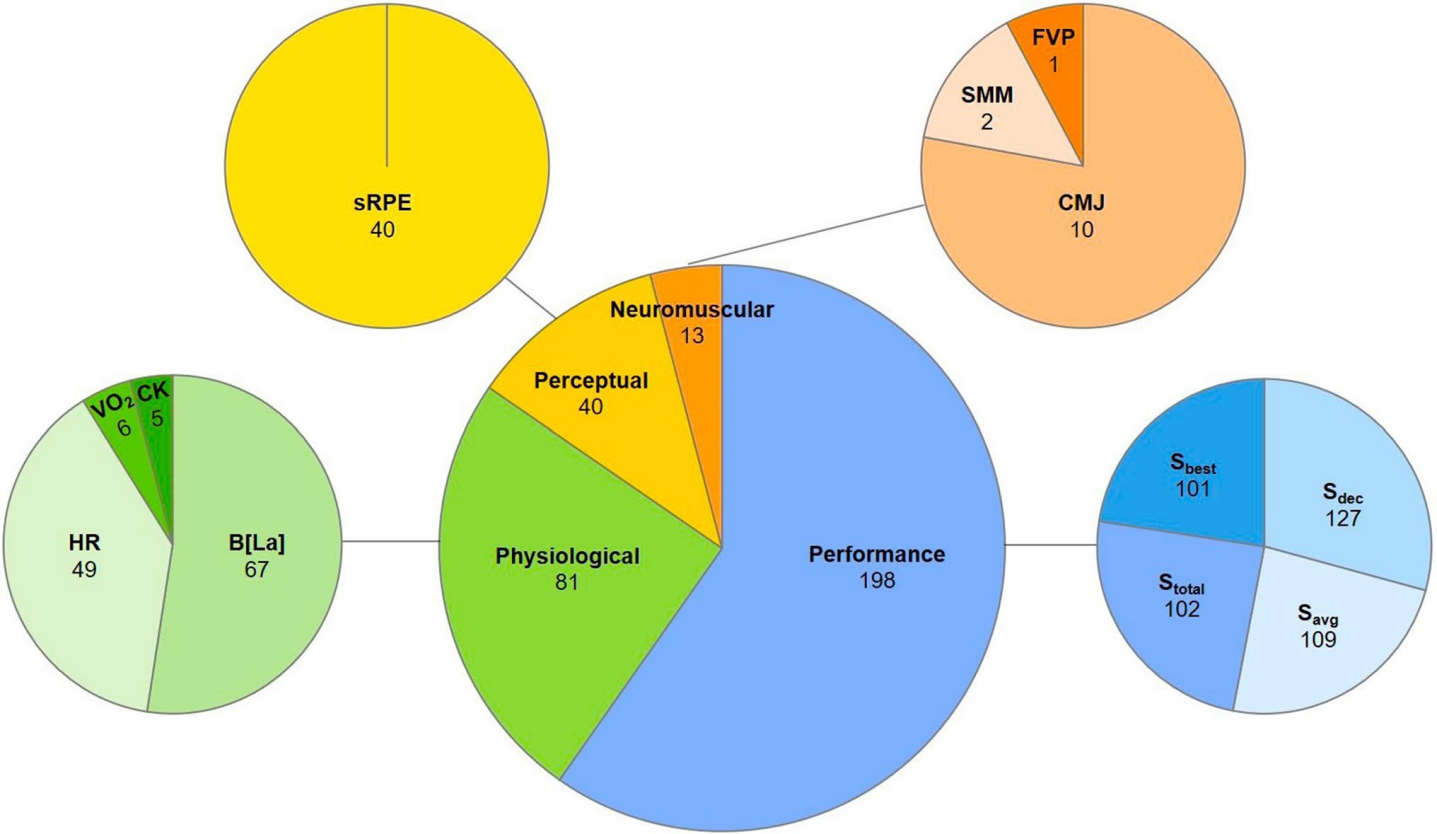
The distribution of outcome measures. Data given as the total number of studies represented (out of 215). *S*_*best*_ best sprint time, *S*_*avg*_ average sprint time, *S*_*total*_ total sprint time, *S*_*dec*_ percentage sprint decrement, *CMJ* counter-movement jump, *SMM* spring-mass model characteristics, *FVP* sprint force–velocity–power profiling, *sRPE* ratings of perceived exertion, *HR* heart rate, *B*[*La*] blood lactate, *CK* serum creatine kinase, *VO*_*2*_ oxygen consumption

**Fig. 3:**
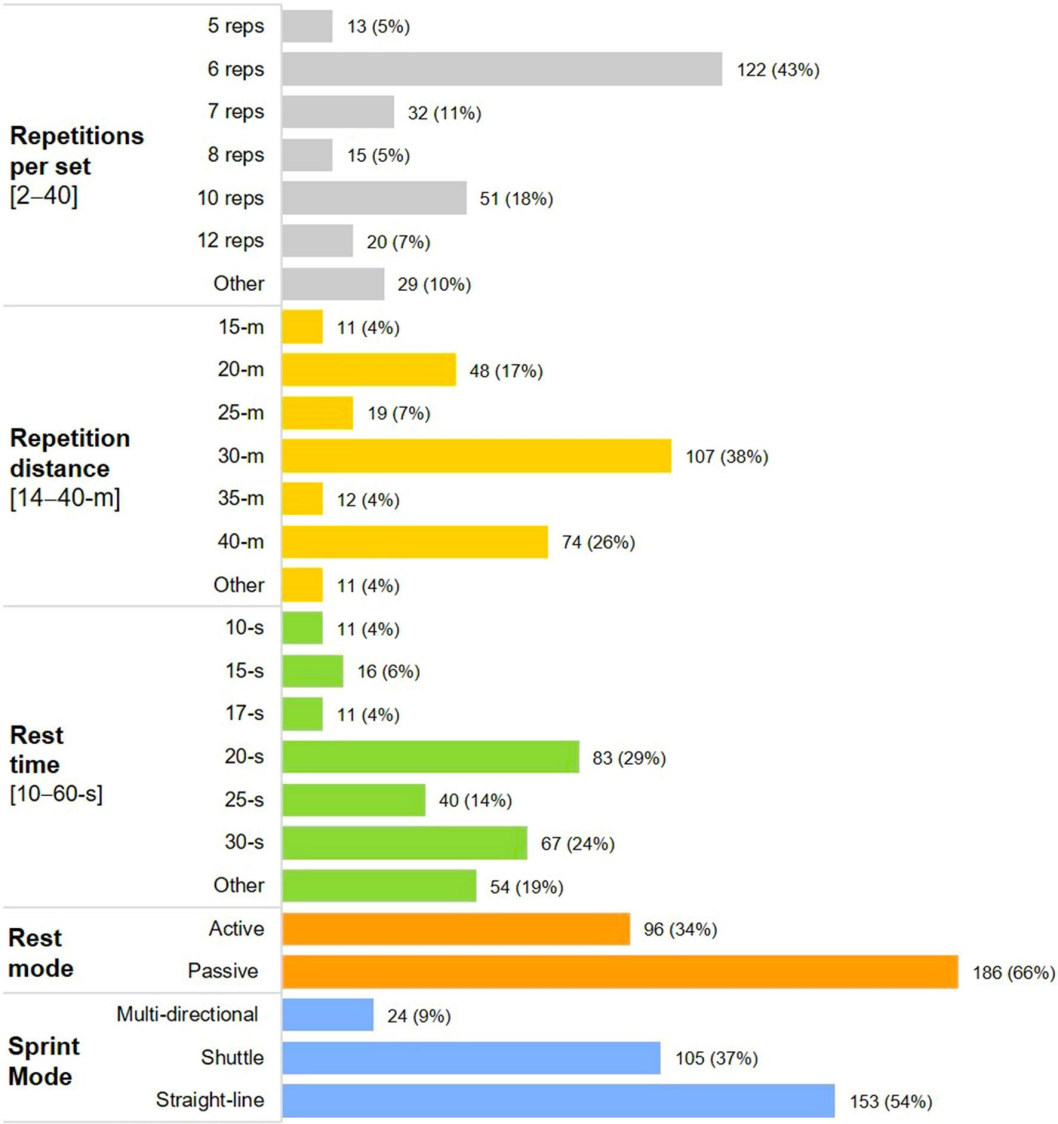
The distribution of RST prescription across all 282 protocols. Data are given as the total number of protocols represented (percentage) [range]

Performance outcomes were represented in 198 (92%) of studies and the most common outcome measure was *S*_dec_ (*n* = 127 studies, 59%) (Fig. 2). The most common prescription of each programming variable were straight-line sprints (*n* = 153 protocols, 54%), performed over 30 m (*n* = 107, 38%), with a passive recovery (*n* = 186, 66%) lasting 20 s (*n* = 83, 29%), prescribed as one set of six repetitions (*n* = 122, 43%; Fig. 3). The majority of protocols (*n* = 263, 93%) employed one set of repeated-sprints, with two sets, three sets and four sets used in five (2%), ten (4%) and four (1%) protocols, respectively. The most common inter-set rest times for all multi-set protocols were 4 (six protocols) and 5 mins (five protocols). The number of 180° changes of direction prescribed for shuttle repeated-sprints ranged from one to two. The most prescribed mode of active recovery was a slow jog back to a one-way start line (*n* = 32 protocols, 33%, i.e. sprints start from one end only). There was one study [33] that strictly enforced a 5 m deceleration zone and one other study [78] that enforced a 6 m deceleration zone.

#### Meta-analysed Acute Demands of Repeated-Sprint Training

The acute physiological, perceptual and performance demands of RST in team sport athletes are presented in Table 3. Also presented are the 90% CI and PI for each estimate, as well as the between sample and between study variation (*σ*).

**Table 3:** Meta-analysed acute physiological, perceptual and performance demands of repeated-sprint training in team sport athletes

Outcome measure		Number of…		Pooled effect			Variation (*σ*, 90% CI) between…	
		Studies	Samples	Estimate	90% CI	90% PI	Studies (*σ*_1_)	Samples (*σ*_2_)
HR_avg_	bpm	12	24	163	154 to 171	131 to 194	16 (11 to 24)	6 (4 to 9)
	% HR_max_	10	21	90	87 to 92	82 to 97	3 (2 to 6)	2 (1 to 3)
HR_peak_	bpm	29	54	182	179 to 184	168 to 195	7 (6 to 10)	2 (1 to 3)
*V*O_2avg_	mL·kg^−1^·min^−1^	6	6	42.4	32.3 to 52.4	16.0 to 68.7	9.2 (0.0 to 20.6)	2.4 (0.8 to 9.4)
B[La]	mmol·L^−1^	64	120	10.7	10.1 to 11.3	5.6 to 15.8	2.6 (2.1 to 3.1)	1.7 (1.4 to 2.0)
sRPE	au (deciMax)	40	68	6.5	6.0 to 6.9	3.5 to 9.5	1.2 (0.7 to 1.6)	1.3 (1.1 to 1.6)
*S*_best_	s	103	191	5.52	5.26 to 5.79	2.79 to 8.25	1.57 (1.40 to 1.79)	0.45 (0.40 to 0.51)
*S*_avg_	s	112	200	5.57	5.31 to 5.82	2.83 to 8.3	1.54 (1.37 to 1.74)	0.57 (0.51 to 0.65)
*S*_dec_	%	125	224	5.0	4.7 to 5.3	1.4 to 8.7	2.0 (1.8 to 2.3)	0.9 (0.8 to 1.1)

Multi-directional protocols are excluded. Heart rate results are independent of each other (HRpeak ≠ HRmax)

*CI* confidence interval, *PI* prediction interval, *HRavg* average heart rate, *% HRmax* percentage of maximal heart rate, *HRpeak* peak heart rate, *VO2avg* average oxygen consumption, *B[La]* blood lactate, *sRPE* session ratings of perceived exertion, *Sbest* best sprint time, *Savg* average sprint time, *Sdec* percentage sprint decrement

#### Moderating Effects of Programming Variables on the Acute Demands of Repeated-Sprint Training

**Fig. 4:**
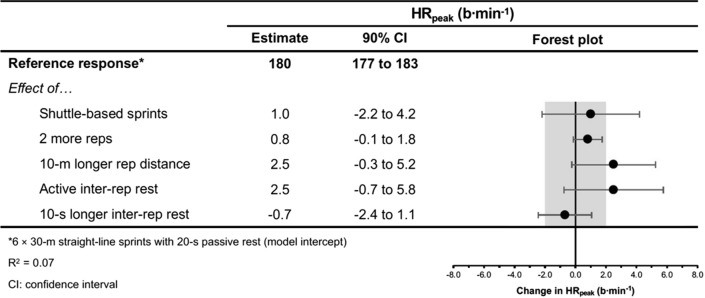
The moderating effects of programming variables on peak heart rate during repeated-sprint training with team sport athletes

**Fig. 5:**
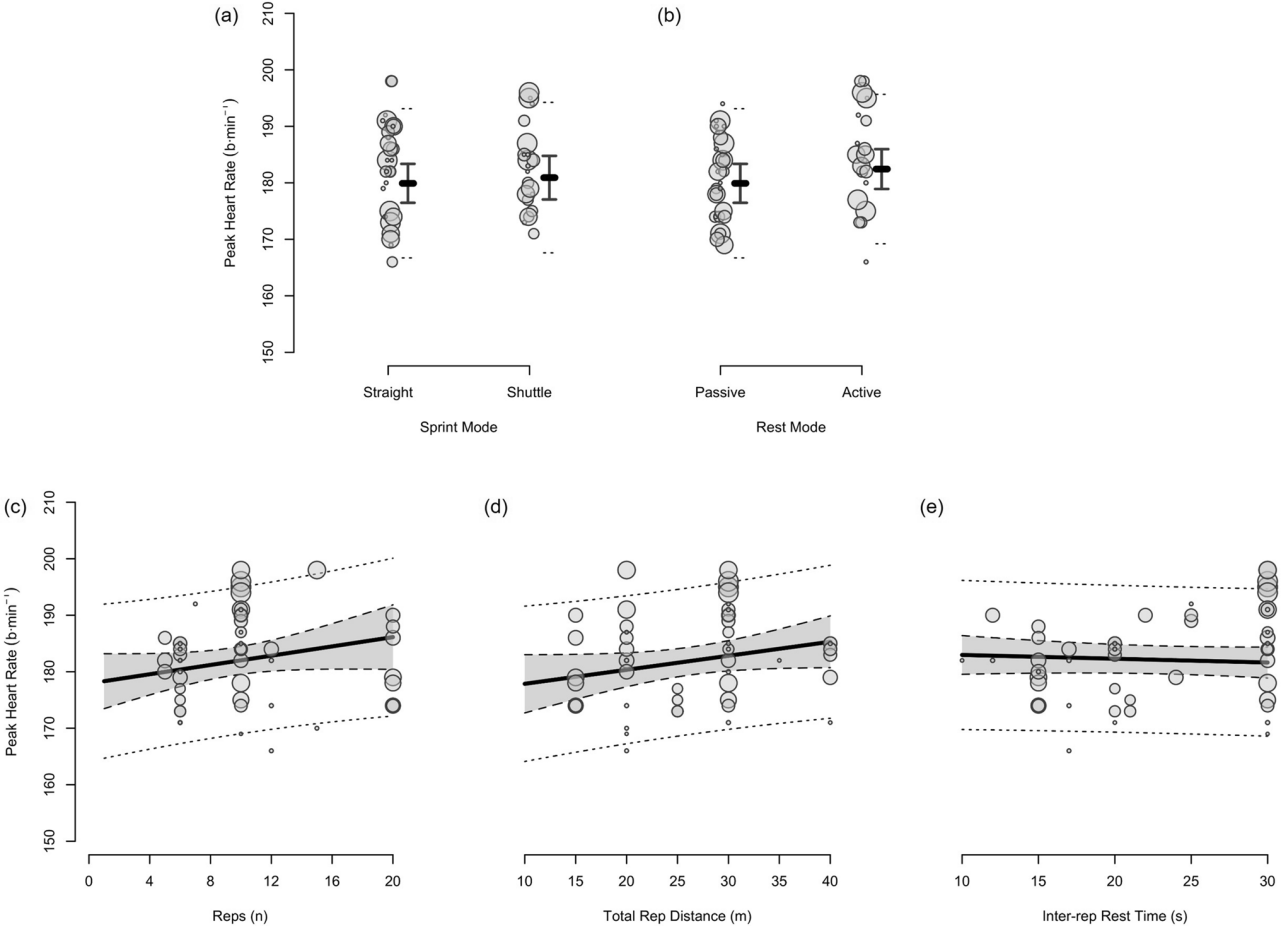
The moderating effects of **a** sprint modality, **b** inter-repetition rest mode, **c** repetitions per set, **d** total repetition distance and **e** inter-repetition rest time on peak heart rate during repeated-sprint training with team sport athletes. Larger circles, greater study size

**Fig. 6:**
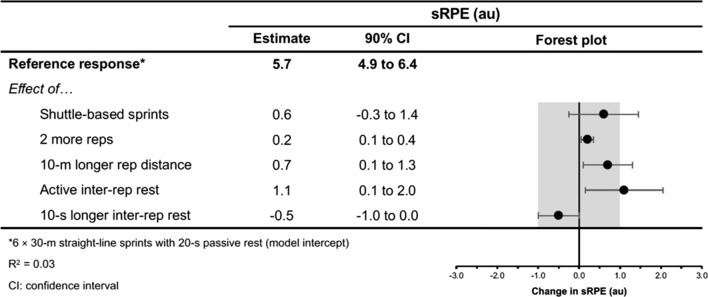
The moderating effects of programming variables on session ratings of perceived exertion following repeated-sprint training with team sport athletes

**Fig. 7:**
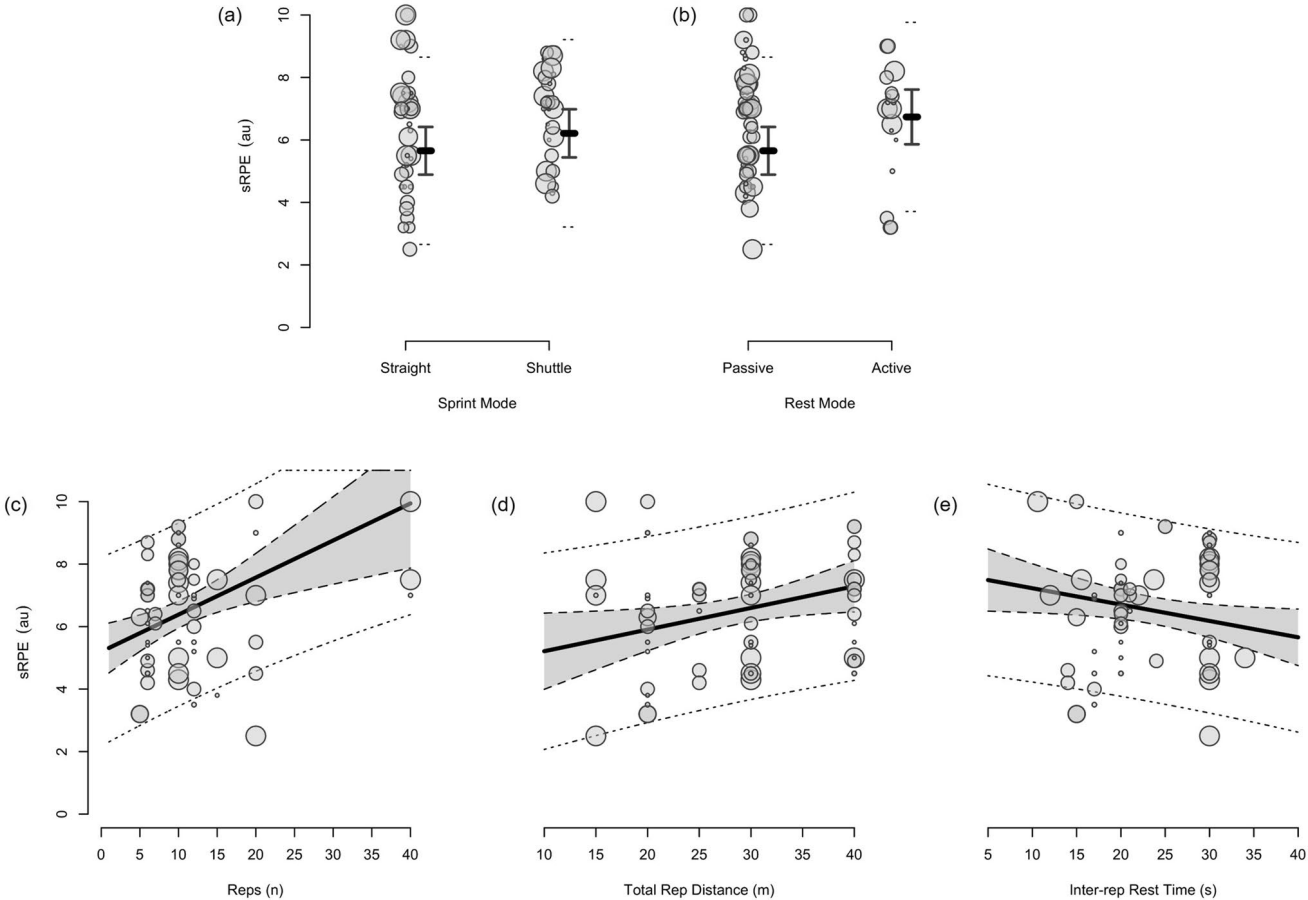
The moderating effects of **a** sprint modality, **b** inter-repetition rest modality, **c** repetitions per set, **d** total repetition distance and **e** inter-repetition rest time on session ratings of perceived exertion following repeated-sprint training with team sport athletes. Larger circles, greater study size

**Fig. 8:**
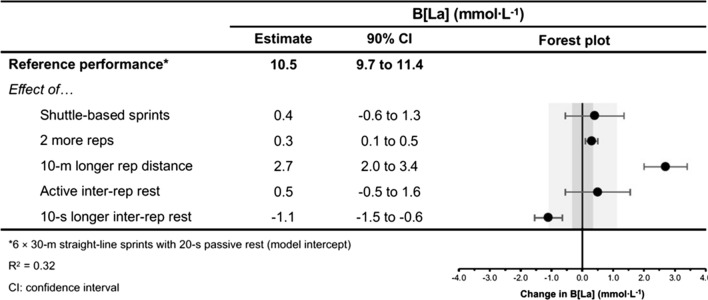
The moderating effects of programming variables on end-set blood lactate following repeated-sprint training with team sport athletes

**Fig. 9:**
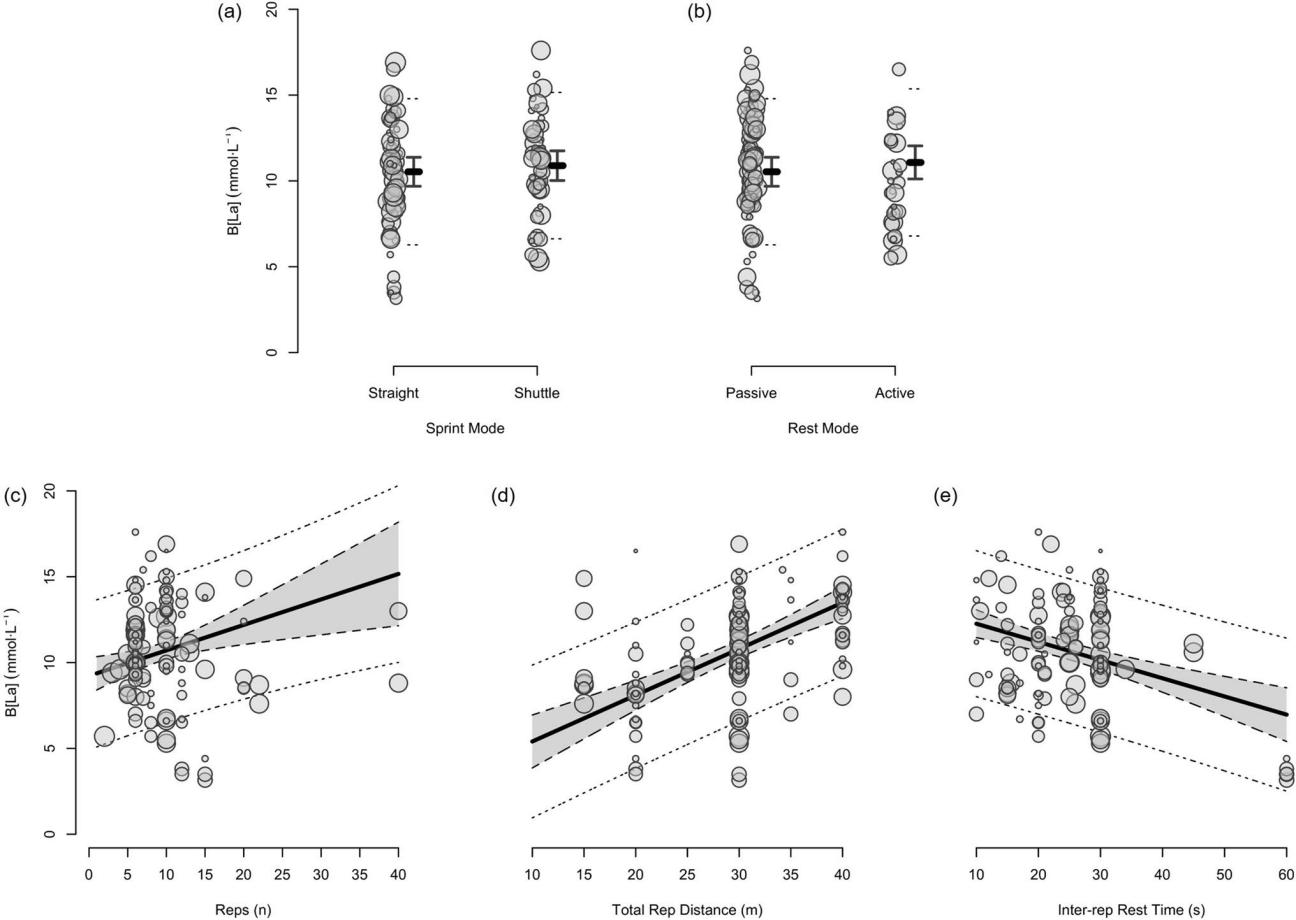
The moderating effects of **a** sprint modality, **b** inter-repetition rest modality, **c** total number of repetitions, **d** total repetition distance and **e** inter-repetition rest time on end-set blood following repeated-sprint training with team sport athletes. Larger circles, greater study size

**Fig. 10:**
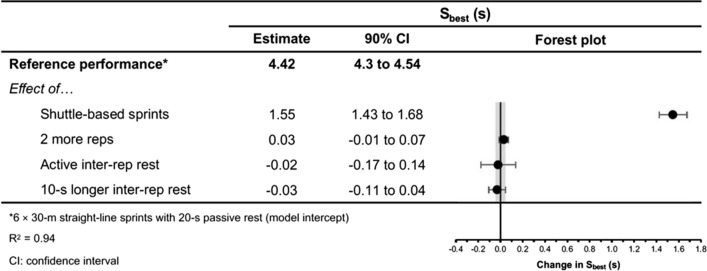
The moderating effects of programming variables on best sprint time during repeated-sprint training with team sport athletes

**Fig. 11:**
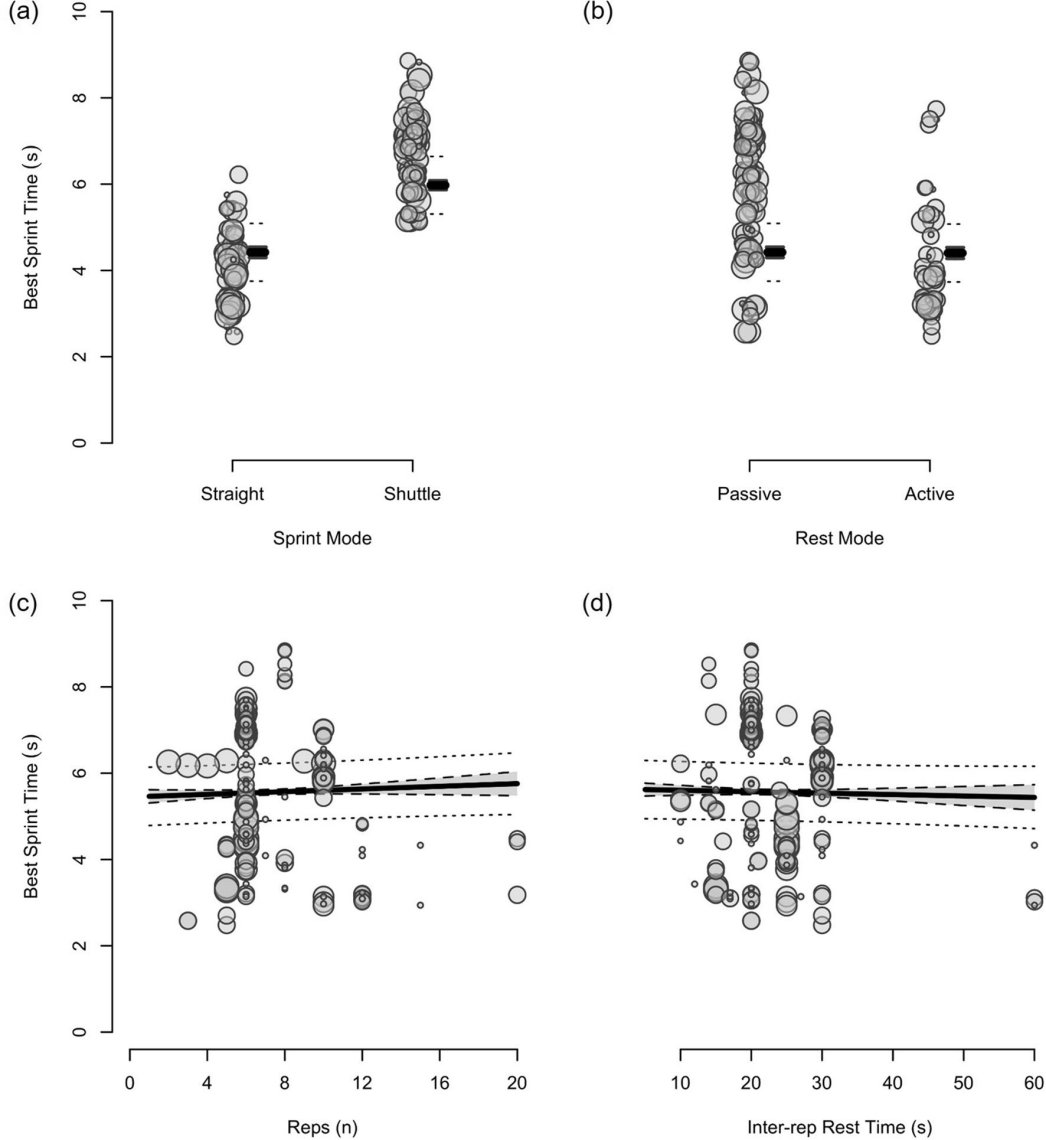
The moderating effects of **a** sprint modality, **b** inter-repetition rest modality, **c** repetitions per set and **d** inter-repetition rest time on best sprint time during repeated-sprint training with team sport athletes. Larger circles,  greater study size

**Fig. 12:**
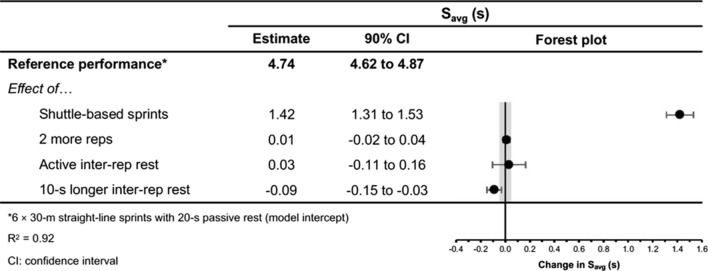
The moderating effects of programming variables on average sprint time during repeated-sprint training with team-sport athletes

**Fig. 13:**
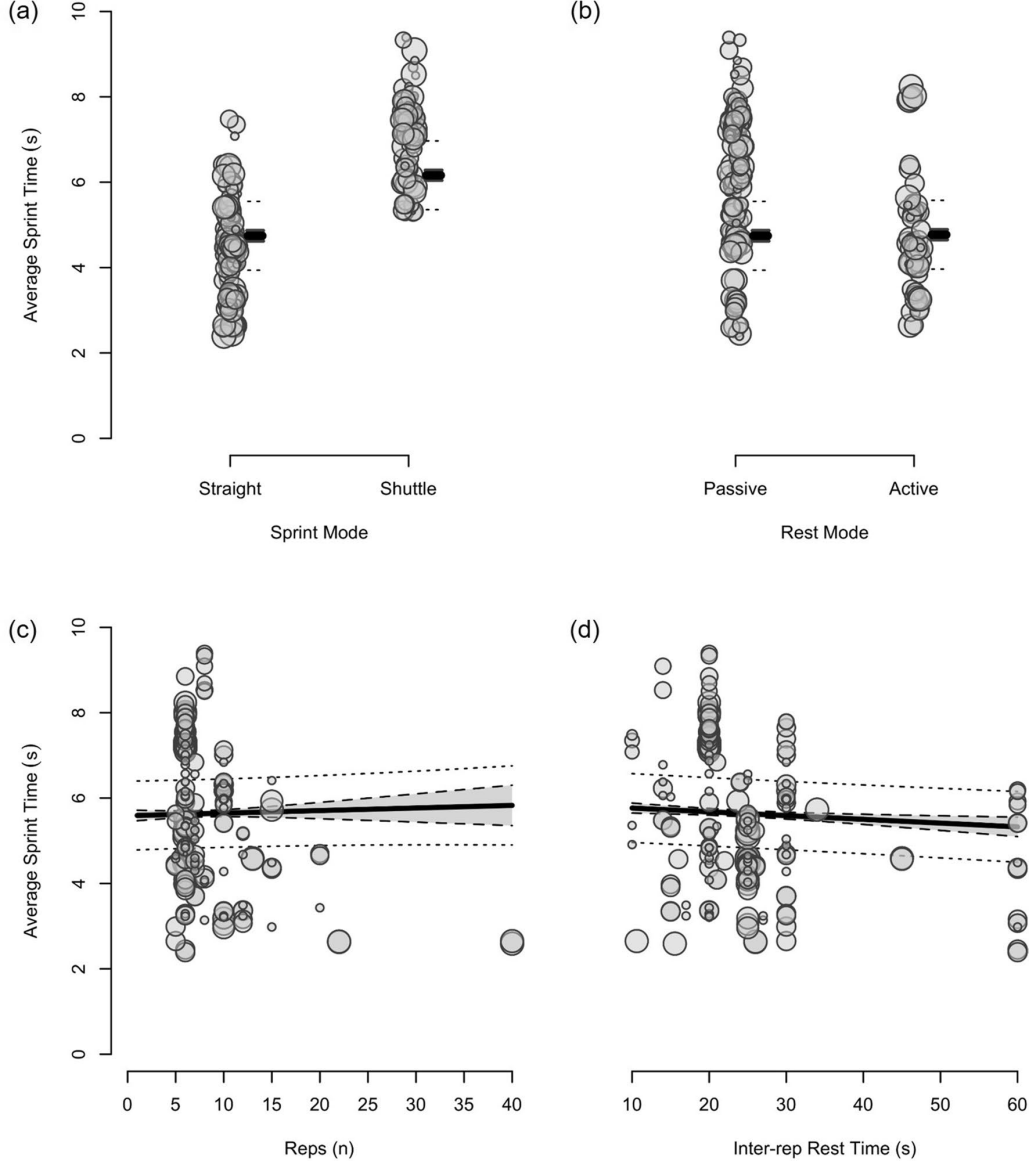
The moderating effects of **a** sprint modality, **b** inter-repetition rest modality, **c** repetitions per set and **d** inter-repetition rest time on average sprint time during repeated-sprint training with team sport athletes. Larger circles, greater study size

**Fig. 14:**
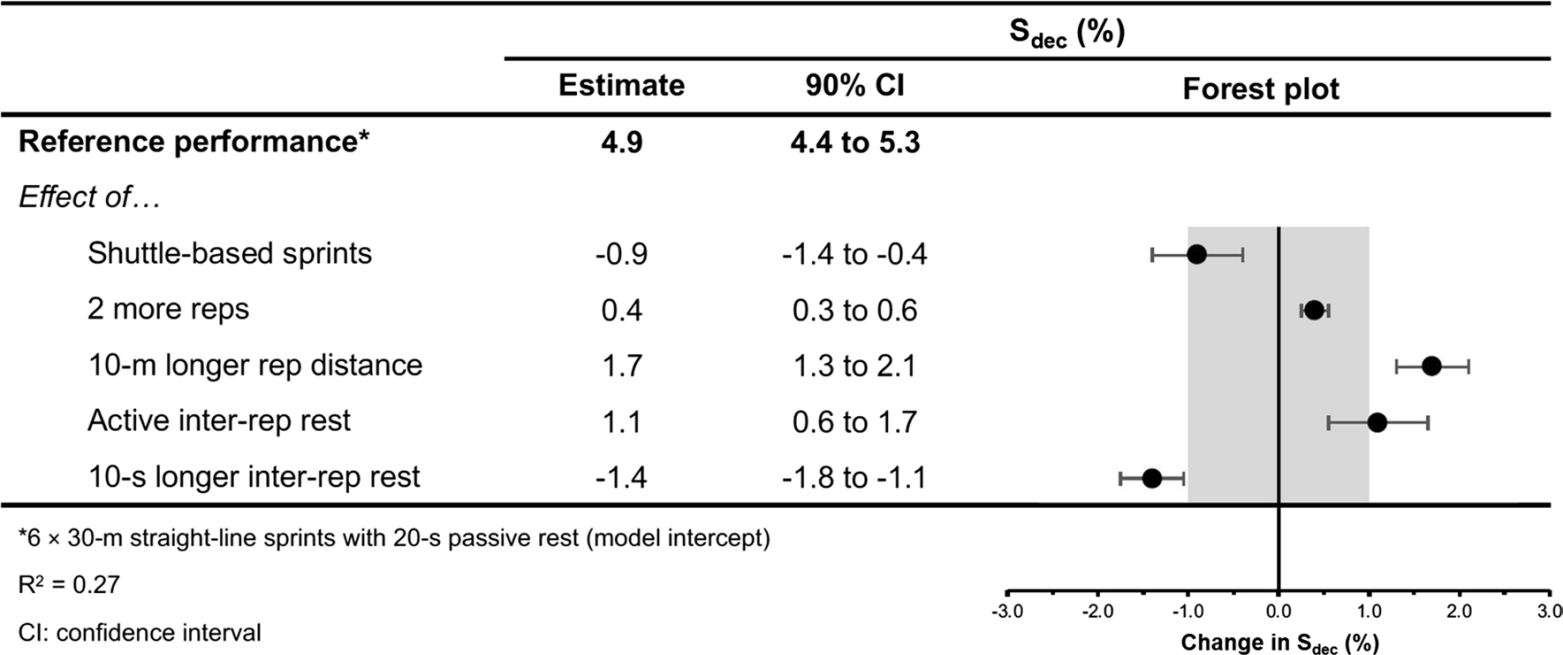
The moderating effects of programming variables on sprint time decrement during repeated-sprint training with team sport athletes

**Fig. 15:**
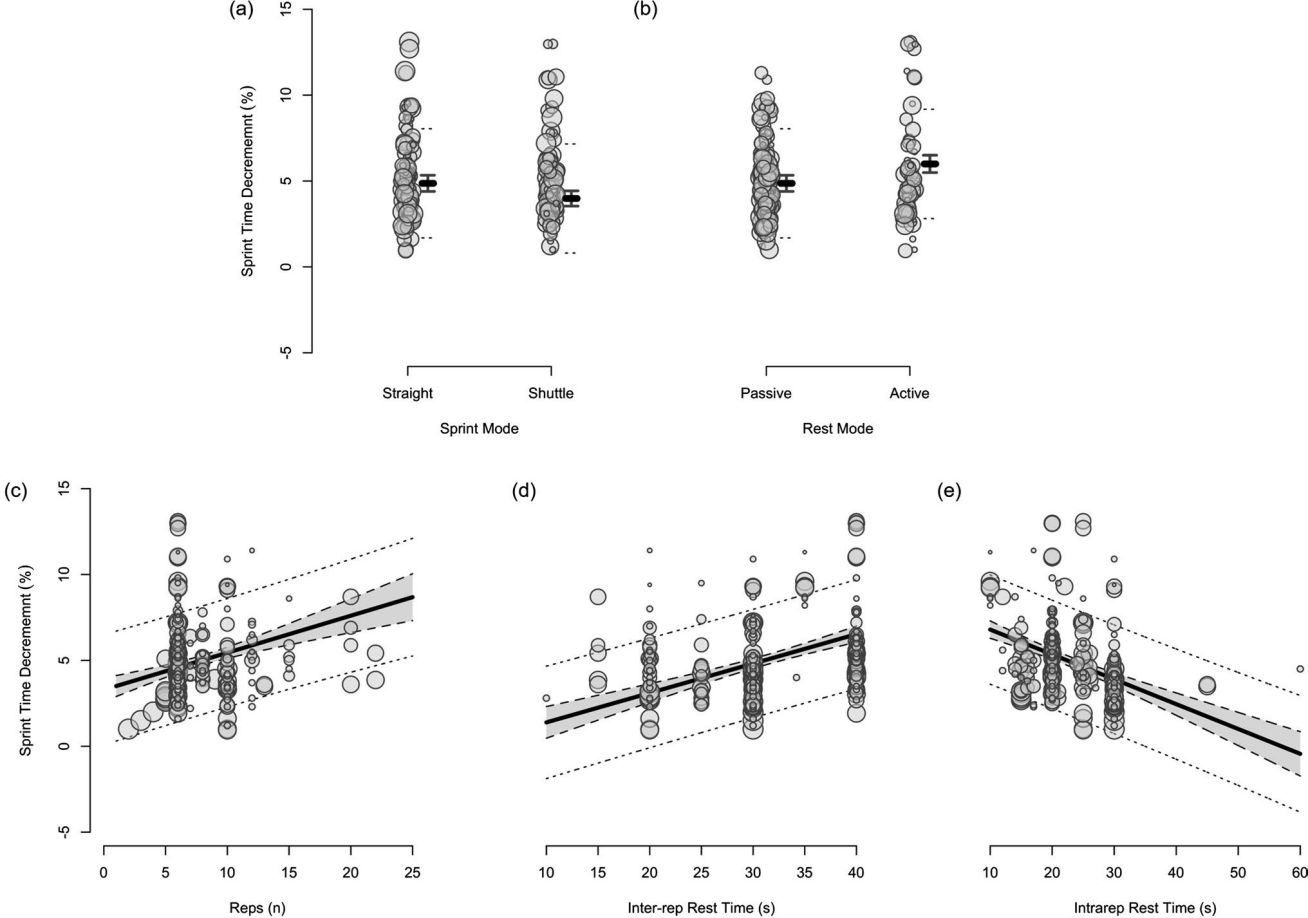
The moderating effects of **a** sprint modality, **b** inter-repetition rest modality, **c** repetitions per set, **d** total repetition distance and **e** inter-repetition rest time on sprint time decrement during repeated-sprint training with team sport athletes. Larger circles, greater study size

The moderating effects of programming variables on the acute physiological, perceptual and performance demands of RST are presented in Figs. 4, 5, 6, 7, 8, 9, 10, 11, 12, 13, 14, 15. All effects were evaluated as the change in each outcome measure when compared with a reference protocol of 6 m × 30 m straight-line sprints with 20 s passive inter-repetition rest. Unless noted in the subsequent sections, moderating effects were deemed inconclusive [i.e. a confidence level (CL) spanning across substantial regions in both positive and negative directions, including the trivial region].

##### Shuttle-Based Sprints

Shuttle-based sprints were associated with a substantial increase in *S*_avg_ and *S*_best_ (i.e. slower times; Figs. 10, 11, 12, 13), whereas the effect on sRPE was trivial (Figs. 6, 7). Performing shuttle-based sprints was compatible with both a trivial and substantial reduction in *S*_dec_ [i.e. a less pronounced decline in sprint times (faster) throughout the set; Figs. 14 and 15].

##### Performing Two More Repetitions Per Set

Performing two more repetitions per set had a trivial effect on HR_peak_ (Figs. 4 and 5), sRPE (Figs. 6 and 7), *S*_avg_ (Figs. 12 and 13), *S*_dec_ (Figs. 14 and 15) and B[La] (Figs. 8 and 9). Additionally, performing two more repetitions per set was compatible with both a trivial and substantial increase in *S*_best_ (i.e. slower time; Figs. 10 and 11).

##### Sprinting 10 m Further Per Repetition

Sprinting 10 m further per repetition was associated with a substantial increase in B[La] (Figs. 6 and 7) and *S*_dec_ [i.e. a more pronounced decline in sprint times (slower) throughout the set; Figs. 14 and 15], whereas the effect on sRPE was trivial (Figs. 6 and 7). Additionally, sprinting 10 m further per repetition was compatible with both a trivial and substantial increase in HR_peak_ (Figs. 4 and 5). The effects on *S*_best_ and *S*_avg_ were not evaluated.

##### Resting for 10 s Longer

Resting for 10 s longer between repetitions was associated with a substantial reduction in B[La] (Figs. 8 and 9), *S*_avg_ (Figs. 12 and 13), and *S*_dec_ (Figs. 14 and 15), while the effects on HR_peak_ (Figs. 4 and 5) and sRPE (Figs. 6 and 7) were trivial. Resting for 10 s longer between repetitions was compatible with both a trivial and substantial reduction in *S*_best_ (i.e. faster time; Figs. 10 and 11).

##### Performing Active Inter-Repetition Rest

Using an active inter-repetition rest modality was compatible with both a trivial and substantial increase in HR_peak_ (Figs. 4 and 5), sRPE (Figs. 6 and 7) and *S*_dec_ (Figs. 14 and 15).

#### Acute Demands of Repeated-Sprint Training on Non-Meta-Analysed Outcomes

The acute demands of straight-line and shuttle RST on non-meta-analysed outcomes are as follows: total sprint time ranged from 7.82 to 86.09 s (number of studies = 102, number of samples = 185), end-set heart rate (HR_post_) ranged from 139 to 191 bpm (*n* = 4 and 12), HR_peak_ as % HR_max_ ranged from 85% to 97% (*n* = 4 and 12), average *V*O_2_ as a percentage of maximal oxygen consumption (*V*O_2max_) ranged from 73% to 83% (*n* = 3 and 6) and creatine kinase measured 24 h post-session ranged from 354 to 1120 µ·L^−1^ (*n* = 6 and 8). The absolute change in CMJ height ranged from 2.4 to −8.6 cm (*n* = 9 and 20) and the percent change ranged from 8% to −27% (*n* = 10 and 21). Results from studies that investigated spring-mass model parameters (*n* = 2 and 2) and sprint force–velocity–power parameters (*n* = 1 and 1) are provided in Supplementary Table S3.

#### Acute Demands of Multi-directional Repeated-sprint Training

The acute demands of multi-directional RST are as follows: *S*_dec_ ranged from 1% to 7% (number of studies = 13, number of samples = 24), *S*_best_ ranged from 4.36 to 8.21 s (*n* = 11 and 19), *S*_avg_ ranged from 4.14 to 8.39 s (*n* = 12 and 22), total sprint time ranged from 32.22 to 83.99 s (*n* = 9 and 11), end-set B[La] ranged from 5.4 to 15.4 mmol·L^−1^ (*n* = 6 and 8), sRPE ranged from 5.5 to 9.1 au (*n* = 6 and 10) and HR_peak_ ranged from 178 to 195 b·min^−1^ (*n* = 6 and 10).

## Discussion

This systematic review and meta-analysis provides the first comprehensive synthesis of the acute demands of RST in team sport athletes. It contains data from 215 studies, 282 repeated-sprint protocols and 4818 athlete inclusions. We demonstrate that physiological, neuromuscular, perceptual and performance demands incurred during RST are consistently substantial; a finding supported by both the meta-analysed point estimates and their 90% prediction intervals (Table 3). Moreover, the magnitude of these acute demands can be influenced by the manipulation of programming variables (Table 4). Prescribing longer sprint distances (> 30 m) and/or shorter (≤ 20 s) inter-repetition rest can increase physiological demands and performance decrement. Conversely, the most effective strategy to mitigate the acute decline in sprint performance is the prescription of longer inter-repetition rest times (≥ 30 s) and shorter sprint distances (15–25 m). The effects of performing two more repetitions per set on our outcomes was trivial, which suggests that prescribing a lower number of successive sprints (e.g. four as opposed to six) may be a useful strategy to reduce sprint volume, while maintaining the desired physiological demands. The influence of shuttle-based protocols and inter-repetition rest modality remain largely inconclusive. These findings from our review and meta-analysis can be used to inform RST prescription and progression in team sport athletes.

**Table 4:** Summary of the effects of programming variables on the acute demands of repeated-sprint training in team sport athletes

	HR_peak_	B[La]	sRPE	*S*_best_	*S*_avg_	*S*_dec_
Shuttle RST	?	?	=	↑	↑	= ↓
Two more repetitions	=	=	=	= ↑	=	=
10 m longer distance	= ↑	↑	=	*	*	↑
Active rest	= ↑	?	= ↑	= ↓	↓	= ↑
10 s longer rest	=	↓	=	↓	↓	↓
Acute demands based on meta-analytic inferences and compared with the reference protocol of 6 m × 30 m straight-line sprints with 20 s passive inter-repetition rest Symbols: ‘=’ indicates ‘trivial’, ‘↑’ substantial increase’, ‘↓’ indicates a ‘substantial decrease’, ‘= ↓’ indicates ‘compatibility with both a trivial and substantial decrease’, ‘= ↑’ indicates ‘compatibility with both a trivial and substantial increase’, ‘?’ indicates ‘inconclusive’ and ‘*’ indicates that the effects were not evaluated. Note: a decrease in *S*_best_ and *S*_avg_ indicates that sprint times are faster *RST* repeated-sprint training, *HR*_*peak*_ peak heart rate, *B*[*La*] blood lactate, *sRPE* session ratings of perceived exertion, *S*_*best*_ best sprint time, *S*_*avg*_ average sprint time, *S*_*dec*_ percentage sprint decrement						

Repeated-sprint training is one method among an array of training options that practitioners can use to enhance the physical performance of team sport athletes. The meta-analytic estimate of sRPE (Table 3) indicates that RST is perceived to be ‘very hard’ (90% PI: ‘moderate’ to ‘extremely hard’), which agrees with the intended prescription of this training modality [18, 79]. Taking into account that a typical RST session lasts for between 10–20 min, the sRPE-training load (sRPE × training duration) is a fraction of that observed during team sport practice [80–82], being approximately 65–130 au (deciMax units). However, this should be considered alongside the physiological and neuromuscular stresses imposed by the RST session. The 10.1–11.3 mmol·L^−1^ reference estimate of B[La] is well above the second lactate threshold (~ 4 mmol·L^−1^) and therefore indicates that there is an immediate and intensive demand placed on the anaerobic glycolytic system during RST [83]. A high rate of anaerobic energy production, accompanied by a B[La] response exceeding 10 mmol·L^−1^, may be an important stimulus to elicit positive long-term changes in enzymes central for anaerobic glycolysis [28, 84]. Therefore, to potentially optimise the anaerobic adaptations to RST for team sport athletes, sessions that cause a B[La] demand of > 10 mmol·L^−1^ should be prescribed. Practitioners should also be conscious of the neuromuscular demands (i.e. impairment in the muscles ability to produce force) imposed by RST, with considerable decrements in CMJ height observed immediately after its implementation. However, while fatigue may be detrimental to acute performance, it also can be important for adaptation [85].

Athletes can reach *V*O_2max_ during RST [86] and the average *V*O_2_ demand is considerable (Table 3), corresponding to approximately 70%–80% of *V*O_2max_ for the normal team sport athlete [87–90]. This also agrees with studies reporting the average *V*O_2_ demands of RST as a percentage of the athletes measured *V*O_2max_ [24, 59, 60]. Training sessions spent with longer periods of time at a high percentage of *V*O_2max_ have been suggested to be an optimal stimulus for enhancing aerobic fitness, particularly in well-trained athletes [79, 91–93]. If the objective is to maximise aerobic adaptations, practitioners should therefore prescribe RST sessions that induce an average *V*O_2_ demand of > 90% max (or > 95% maximal heart rate) [79, 94], which could be achieved by manipulating certain programming variables in isolation and/or combination. Although moderator analysis of *V*O_2_ was not feasible due to a low number of samples, qualitative synthesis indicates that longer sprint distances [86], active rest periods [24] and shuttle-based RST [59, 60] can amplify the *V*O_2_ demands. While RST is a time-efficient training method that can induce small to large improvements across a range of physical parameters [8, 9], practitioners should, however, consider that RST is unlikely to be the best tool for eliciting time at or near *V*O_2max_ and ultimately, for enhancing aerobic fitness [9, 79]. Pursuing utmost change in this area by implementing excessively demanding protocols could mitigate the improvement of other physical qualities (e.g. speed). Manipulating programming variables based on the goals of the training program is therefore crucial to regulate the acute demands of RST and optimise specific adaptations.

### Sprint Modality

There were a greater number of RST protocols that prescribed straight-line sprints (*n* = 153, 54%) compared with shuttle RST (*n* = 105, 37%) and multi-directional RST (*n* = 24, 9%). Across the 24 protocols that prescribed multi-directional repeated-sprints [46, 95–111], there were a variety of different designs and angles implemented, ranging from 45° to 135°, for 2–5 changes of direction. Given the multitude of programming variables to consider, meta-analysis of multi-directional RST was not feasible. Nonetheless, we found that consistently high HR_peak_ (178–195 bpm and 92%–100% HR_max_), sRPE (5.5–9.1 au) and post-session B[La] (5.4–15.4 mmol·L^−1^) were reported across all multi-directional protocols. Multi-directional sequences were designed to replicate specific movement demands of team sports, where rapid changes of direction are common [5, 112, 113]. Moreover, previous research has identified that straight-line speed and change of direction ability are different physical qualities because of their distinct biomechanical determinants [112, 113]. Greater application of multi-directional and shuttle-based RST may therefore be used to help develop change of direction ability, but practitioners should be aware of the acute demands of each modality.

Compared to straight-line RST, our meta-analysis shows that sprint times are clearly slower during shuttle-based RST (Figs. 10 and 12), but *S*_dec_ is less (Fig. 14). Practitioners can therefore expect slower sprint velocity when changes of direction are implemented, but athletes may be able to better sustain their initial sprint performance. The effects on HR_peak_ and B[La] were inconclusive (Figs. 4 and 8), while the effect on sRPE was mostly trivial (Fig. 6), which may suggest that these physiological and perceptual demands of RST are independent of sprint modality. It should be noted, however, that the acute demands of RST performed with changes of direction are conditional to the number and angle of direction changes, the distance between each direction change and the duration of the sequence [60, 99, 106, 114, 115]. These factors affect the absolute speeds that are attained and the muscular work performed during the sprint, propulsive and braking components. Additionally, by integrating changes of direction into RST, there is accumulation of acceleration and deceleration which can increase the neuromuscular demand [99]. This seems evident by greater reductions in CMJ height following shuttle-based RST [104, 116, 117].

Shuttle-based sprints can be applied during a RST program to emphasise change of direction, limit absolute running speeds and induce a similar physiological demand to straight-line RST. There may be instances, such as towards the end of season, where practitioners want to limit the physiological stress on the athlete during shuttle or multi-directional RST. In these cases, it has been demonstrated that decreasing the sprint duration through time-matched protocols is an effective strategy [99]. Therefore, when designing RST, practitioners need to consider the influence of the direction changes on the duration of the sprint, rather than just the overall distance, as this can have a marked effect on the internal demands [99]. Of course, straight-line sprints should be implemented if the goal is to expose athletes to higher speeds.

### Number of Sprint Repetitions and Sets

Repeated-sprint training is implemented in research and practice to target a broad range of outcomes, which is reflected by considerable variation in the number of sprint repetitions prescribed across studies (range 2–40 repetitions per set). The vast majority of protocols (*n* = 257, 94%) implemented just one set, with six repetitions the most prescribed number of sprints per set (*n* = 122 protocols, 43%). Protocols that prescribed ≥ 12-repetitions per set [19, 33–35, 61, 62, 86, 118–128] were often designed to induce a high degree of fatigue. Accordingly, high creatine kinase responses (542–1127 µ·L^−1^) were reported in studies prescribing high repetition protocols [33–35, 123], despite longer inter-repetition rest times (≥ 30 s). These long-series of exhaustive efforts are counterintuitive to the movement demands of team sports, where sprint efforts are more likely to occur in small clusters [129, 130]. While the moderating effects of the number of sets per session was not meta-analysed due to the low number of samples, it is worth noting that with an increasing number of sets, sprint times decayed and heart rate was increased, but changes in B[La] seem negligible [58, 122, 131]. Further investigation is required to better understand the impact of the number of sets performed per session, as well as the overall session volume, on the acute demands of RST.

A substantial physiological demand is induced with the prescription of just six sprint repetitions, as demonstrated by the estimates and PI’s for HR_peak_ and B[La] (Figs. 4 and 8). A large cardiac demand, inferred by the 182 bpm reference estimate of HR_peak_, coupled with a B[La] response exceeding 10 mmol·L^−1^, provide a strong aerobic and anaerobic stimulus, which may underpin the improvements in high-speed running abilities observed after RST interventions [2, 8]. With the reference estimate of B[La] above 10 mmol·L^−1^ and HR_peak_ close to maximal after six repetitions, further pursuing small increases in these acute physiological outcomes by performing more repetitions does not seem worthwhile. Our meta-analytic estimates show that the effects of performing two more repetitions per set was trivial on all outcome measures except *S*_best_, which was compatible with both trivial and substantial effects (Fig. 10). Therefore, other programming factors appear to have a greater effect on physiological, perceptual and performance outcomes. Crude estimation of the number of additional sprints required for the point estimate of each outcome measure to equal the minimum practically important difference reveals an unrealistic and impractical expectation. For example, the number of additional repetitions needed to increase sRPE by a one-unit scale change in our data is ten (i.e. 16-repetitions per set in total). This increase in volume and the neuromuscular demands of high repetition sets (greater than ten repetitions) may induce excessive muscle damage [33–35, 123]. Moreover, large numbers of repetitions can result in ‘pacing’ strategies that influence the maximal nature of RST and accumulated fatigue reduces the effectiveness of later sprints [132]. This is supported by our findings that show a *S*_dec_ of 1.2% would be expected to occur in studies (groups) performing 6 more repetitions (i.e. 12-repetitions per set in total) [77]. Therefore, excessive numbers of sprint repetitions can exacerbate fatigue and cause sub-optimal performance during RST.

Lower numbers of repetitions per set (e.g. greater than six repetitions) may be a more effective programming approach during competition periods to reduce training volume while still providing a potent physiological stimulus and allowing for the quality of each repetition to be maintained. In this regard, the trivial reduction expected in each outcome measure when performing four versus six repetitions may be beneficial, when viewing from a risk-reward perspective. However, a one-size-fits-all approach regarding the RST prescription for team sport athletes can lead to some athletes being under-stimulated, while others can be overloaded, depending on the athletes’ speed and fitness profile [133, 134]. When the number of repetitions performed is fixed, there is considerable inter-individual variation in the degree of fatigue experienced across the same group of athletes [48]. This can be incurred despite two athletes having similar maximal aerobic speeds but different maximal sprinting speeds (i.e. differences in anaerobic speed reserve) [134, 135]. In our review, all studies, except one [57], prescribed a fixed number of repetitions. However, in the study by Akenhead et al. [57] the level of relative sprint decrement (5%) was prescribed with a ‘flexible’ repetition scheme, which allowed more control over the magnitude of fatigue accrued by all participants. By prescribing a level of relative sprint decrement or relative performance threshold, instead of a fixed number of repetitions, practitioners can individualise RST prescription. This could provide practitioners with the ability to autoregulate training load based on differences in physical capacities and fluctuations in prior fatigue.

### Sprint Distance

A sprint distance of 30 m was most implemented (*n* = 107 protocols, 38%), which is longer than the average sprint distance typically observed during field-based team-sports competitions (15–25 m) [136]. Additionally, 40 m was the longest sprint distance prescribed (*n* = 74, 26%). This distance is commonly used as a proxy measure of maximal speed in team sport athletes [137, 138], as it can allow maximal velocity to be reached when it is applied in a straight-line format. Furthermore, both 30 m and 40 m were often implemented as a shuttle format, with one to two changes of direction. A distance of 14 m was the shortest sprint effort prescribed, represented in two protocols [139], while 15 m was prescribed in 11 (4%) protocols. Compared with longer sprints (> 30 m), these shorter distances emphasise the acceleration phase of sprinting and were often applied with court-based athletes (i.e. basketball and handball) [122, 139–141]. Shorter distances may better reflect the competitive environment of court-based team sports where players are engaged in sprint efforts of 15 m and less [119, 142, 143].

Despite the prevalence of studies implementing a sprint distance of 30 m, altering the distance of each sprint effort by 10 m had the largest moderating effect on B[La] (substantial increase), *S*_dec_ (substantial increase [more pronounced decline in sprint times]) and HR_peak_ (compatible with a trivial and substantial increase). Longer sprints increase phosphocreatine (PCr) depletion and glycolytic activity, while also resulting in an increased accumulation of metabolic by-products (e.g. hydrogen ions, inorganic phosphate) [1, 136]. Furthermore, longer sprints provide exposure to faster absolute running speeds and higher vertical ground reaction forces that are attained via upright running mechanics [144, 145]. This is compared with shorter distances, where the athlete spends a high proportion of time in the acceleration phase, resulting in a greater horizontal propulsive force, but smaller braking force [144, 145]. Consequently, there can be a greater strain on the musculoskeletal system during longer sprints [146–148]. This is evident through greater declines in sprint kinematics (i.e. vertical stiffness and centre of mass vertical displacement) when longer sprint distance (35 m versus 20 m) was prescribed in two studies that investigated spring-mass model characteristics [54, 149]. Despite a greater physiological and neuromuscular demand imposed by longer sprints, the effect of a 10 m longer sprint on sRPE was trivial (Fig. 6). This suggests that greater distances can be prescribed without inducing a practically substantial increase in perceived exertion.

When beginning a RST program, shorter distances (15–25 m) are a more conservative option that can be used to limit metabolic stress and neuromuscular strain. It may also be beneficial to prescribe shorter distances during maintenance/taper sessions or for athletes who may never be exposed to longer sprints during competition (e.g. court-based athletes, goalkeepers). Training progression and overload can then be achieved by gradually increasing distance (> 30 m) with a view to expose athletes to faster absolute running speeds, greater fatigue and a high physiological demand. This could be implemented during preparation phases before commencing high-intensity training drills and match-play, or during late-stage return to play following injury.

### Inter-repetition Rest Duration

There was considerable heterogeneity in the distribution of inter-repetition rest duration across the protocols, which ranged from 10 to 60 s. This was partly due to differences in the approach to rest prescription, whereby pre-determined times, time-cycles and work-to-rest ratios were all employed in different literature. A 10 s rest duration was prescribed in 11 (4%) protocols, but such short rest may make it difficult for athletes to safely decelerate and make it back to the start-line in time for the next sprint. The most common rest durations were 20 s and 30 s, represented in 83 (29%) and 67 (24%) protocols, respectively. These rest durations are similar to the amount of recovery time typically afforded between sprints during team sport competition [129, 130]. A 60 s rest duration was implemented in 9 (3%) protocols.

Shorter rest times (e.g. 10 s versus 20 s) are associated with slower sprint times, greater performance fatigue and an increased metabolic response. Additionally, shorter rest may lead to greater decrements in CMJ height following RST [150]. Inversely, longer inter-repetition rest times (e.g. 30 s vs 20 s) have a substantial influence on the reduction of B[La] and allow for sprint performance to be better maintained across a set (i.e. faster *S*_avg_ and lower *S*_dec_). This is likely due to greater clearance of metabolic by-products and increased PCr resynthesis [1, 121]. An interesting finding of our study was that a 10-s longer inter-repetition rest had a trivial effect on HR_peak_ and sRPE. Longer inter-repetition rest may allow athletes to perform each repetition with greater speed [151] and reduce the desire for pacing. Furthermore, longer rest would be expected to increase set duration, thereby allowing both heart rate and *V*O_2_ to increase with time [86, 106, 122]. It is possible, however, that the cardiorespiratory demand could be blunted if prolonged rest times (e.g. 60 s) are implemented. This was demonstrated in a group of well-trained university students where *V*O_2_ was 9% less when 60-s rest times were used during RST, compared with 30 s rest [151].

Collectively, our findings support the use of longer rest durations (≥ 30 s) to reduce within session fatigue and maintain repetition quality. Longer rest times could therefore be implemented during periods of fixture congestion to reduce player fatigue during RST, or used during the intensification stage of a pre-season to maximise sprint performance [19]. Additionally, longer rest times are recommended when longer sprint distances are prescribed, which can help account for the extended work duration of these sequences. However, longer rest durations reduce the metabolic demand of RST, which could limit certain physiological adaptations (e.g. maximal accumulated oxygen deficit, changes in glycolytic enzymes) [28, 152] and performance in activities that require a substantial anaerobic component [19]. Therefore, shorter rest durations (≤ 20 s) can be prescribed to induce greater levels of fatigue, which could help prepare team-sport athletes for peak periods of a match, where sprint efforts can be interspersed with minimal rest [129, 130].

### Inter-repetition Rest Modality

There were a higher number of protocols that implemented passive inter-repetition rest (*n* = 186, 66%), as opposed to an active rest period (*n* = 96, 34%). Active recovery protocols were commonly combined with inter-repetition rest durations of ≥ 25 s. Most protocols that prescribed an active recovery involved a slow jog at pre-defined running speeds (e.g. 2 m⋅s^−1^) or self-selected speeds, which were often returning to a one-way start line. Other active recovery protocols implemented faster running speeds such as 8 km⋅h^−1^ [23, 118] and 50% of maximal aerobic speed [24, 86, 153, 154]. When these faster running speeds were prescribed, the physiological demands (i.e. heart rate, *V*O_2_, B[La]) were amplified and there was a greater *S*_dec_ compared with passive rest and active rest performed at a slow jog [24, 153–155]. Repeated jumps were performed during the inter-repetition rest period in two studies [59, 156], which increased the cardiorespiratory and muscular demands [59, 156]. However, the internal demands are likely to be more varied compared with a precise running intensity.

The findings of our meta-analysis suggest that active rest may cause a substantial increase in HR_peak_ (Fig. 4), sRPE (Fig. 6) and *S*_dec_ (Fig. 14), although we acknowledge that these effects are also compatible with trivial values (i.e. there could be no substantial influence). Active recovery limits the oxidative potential for PCr resynthesis before each sprint, which affects the maintenance of muscle power [24, 133, 150]. This leads to greater declines in anaerobic work capacity and subsequently, repeated-sprint performance. On the contrary, passive recovery is associated with an enhanced PCr resynthesis and as our results confirm, a smaller *S*_dec_ [157, 158]. While there were no substantial differences in B[La] (Fig. 8), our meta-analysis does not consider the intensity of the recovery period, which ultimately determines the extent of the acute demands [59, 153, 157].

The prescription of active recovery might amplify the physiological and perceptual demands to RST, as well as performance decrement, without increasing the sprint volume. This could be achieved, for example, by prescribing active recovery at an intensity of ≥ 50% maximal aerobic speed. It would be practical to implement this with a standardised recovery-run distance and rest durations of ≥ 25 s to allow the athlete to gradually decelerate from the sprint component into the recovery running speed. Yet, once again, acknowledging that the influence of active recovery on HR_peak_, sRPE and *S*_dec_ were compatible with both trivial and substantial effects, we advise practitioners to place more emphasis on recovery duration for manipulating RST acute demands at present. For this reason, future research should examine the effects of specific active recovery intensities on RST physiological, perceptual, neuromuscular and performance demands.

### RST Protocols with Additional Modifications

The use of additional modifications to RST can be applied to augment or attenuate internal demands. Short enforced deceleration zones (< 10 m), which were prescribed in two studies [33, 78], reduce sprint performance and exacerbate the magnitude of muscle damage due to the large eccentric forces applied during rapid braking, which is further accentuated when higher numbers of repetitions are performed. Gradual deceleration zones (> 10 m) are therefore recommended to mitigate undue muscular damage. Performing repeated jumps within the inter-repetition rest period may be an effective strategy to induce a greater physiological stimulus during RST, while exposing athletes to sport-specific actions, without an increase in the volume of high-intensity running [59, 156]. When jumps were prescribed in studies by Buchheit et al. [59] and Padulo et al. [156], very high B[La] (10.2–13.1 m⋅mol^−1^), HR_peak_ (96%–97% heart rate max) and sRPE (7.9–8.0 au) were observed. The additional muscular work performed during the recovery period with jumps has previously been shown to increase muscle deoxygenation of the lower limbs, but it should be noted that these sequences are also likely to reduce acute sprint performance [59, 156]. Furthermore, with only two studies investigating the effects of jumps within the inter-repetition rest period, the optimal volume and intensity of these actions are yet to be established. There is potential for other modifications to be implemented during RST, such as sport-specific skills (e.g. passing, shooting), grappling, push-ups and tackling into contact bags. These types of explosive efforts typically precede or follow high-intensity runs/sprints during match play [159–161] and may help to better simulate the physiological demands associated with competition. Furthermore, flying sprints that incorporate a submaximal acceleration zone may provide exposure to repeated bouts of maximal velocity sprinting, without the neuromuscular demands of rapid acceleration [162].

### Limitations

There are several important issues to consider when interpreting our findings. Depending on the outcome measure, a proportion of the variation in the meta-analysed acute demands of RST can be explained by factors other than the programming variables investigated (Supplementary Table S4). Factors directly related to individual differences in human physiology have been shown to influence the acute demands to RST, such as age [36, 100, 101, 111, 163–166], fitness level [167], playing status [46, 168–174], gender [131, 139, 175, 176] and ethnicity [177]. Furthermore, a proportion of the variation in the acute demands may also be due to the impact of programming variables not investigated (e.g. number of sets), as well varied data collection methods, conditions and reporting. For example, there are inter- and intra-individual differences in B[La] accumulation depending on sampling procedures (time and site), hydration status, previous exercise and ambient temperature [18, 47, 178]. Nevertheless, the influence of the latter factors on the present review are likely to be low considering that item ten in the inclusion–exclusion criteria ensures that RST must have been performed under normal conditions (e.g. hydrated state, ≤ 30 °C) and without fatiguing exercise occurring in the previous 24 h. We also appreciate the concerns of comparing CMJ height between different methods and devices [179], which is why CMJ outcomes were not meta-analysed.

When interpreting acute heart rate and *V*O_2_ responses to training, it is important to consider the starting value at the commencement of exercise, which will influence the magnitude of change. However, the majority of studies did not present this information, and thus, we were unable to account for this in our analyses. Additionally, there was an insufficient number of samples to determine the moderating effects of programming variables on average heart rate and *V*O_2_. There was also a low number of samples for HR_peak_ as % HR_max_, creatine kinase, spring mass-model parameters and sprint force–velocity–power parameters, which meant we were unable to meta-analyse these outcomes. Therefore, in future, researchers may wish to investigate the effects of RST on these outcomes. Finally, it should be noted that while our elected reference adjustments of 10 m and 10 s allow for comparison between sprint distance and inter-repetition rest time, respectively, this will not always represent the same relative change (i.e. an increased sprint distance from 10 m to 20 m represents a 100% change, while 30 m–40 m represents a 25% change). Therefore, this information should be treated with caution and used within the context of the programmed session.

## Conclusions

Our systematic review and meta-analysis is the first to summarise the acute physiological, neuromuscular, perceptual and performance demands of RST in team sport athletes, while providing a quantitative synthesis of the effects of programming variables. RST provides a potent physiological stimulus for the physical development of team sport athletes, with the magnitude of the acute demands influenced by several programming variables (Table 4). Longer sprint distances and shorter inter-repetition rest periods are the most efficacious strategies to increase RST demands. When manipulated in combination, these factors are likely to have an even greater effect, from which the magnitude of within-session fatigue and acute training response can be expected to follow. Reducing the number of repetitions per set (e.g. four as opposed to six) can maintain the physiological, perceptual and performance demands of RST while reducing sprint volume. When combined with shorter sprint distances and increased inter-repetition rest periods, this might be a useful strategy during strenuous training and competition periods [26]. Additionally, straight-line, shuttle and multi-directional repeated-sprints can be prescribed to target movement specific outcomes, depending on the aims of the training program. While there is a large quantity of evidence relating to acute performance outcomes of RST, there is a lack of literature on cardiorespiratory (e.g. *V*O_2_) and neuromuscular demands. The insights from our review and meta-analysis provide practitioners with the expected demands of RST and can be used to help optimise training prescription through the manipulation of programming variables.

## Supplementary Information

Below is the link to the electronic supplementary material.

Supplementary file 1 (PDF 1467 KB)

## Declarations

### Data Availability

All data and material reported in this systematic review and meta-analyses are from peer-reviewed publications. All extracted data is available in Supplementary Tables S2 and S3.
