# Supplementary material for: The Acute Demands of Repeated-Sprint Training on Physiological, Neuromuscular, Perceptual and Performance Outcomes in Team Sport Athletes: A Systematic Review and Meta-analysis
Source: Sports Med. 2023 May 24;53(8):1609–40. doi: 10.1007/s40279-023-01853-w (PMC10356687; doi:10.1007/s40279-023-01853-w)
Supplement: Supplementary file 1 — (PDF 1467 KB) [file 40279_2023_1853_MOESM1_ESM.pdf]

Full title: The Acute Demands of Repeated-Sprint Training on Physiological, Neuromuscular, Perceptual and Performance Outcomes in Team Sport Athletes: A Systematic Review and Meta-Analysis

Running heading: Acute Demands of Repeated-Sprint Training

Authors: Fraser Thurlow[1,3], Jonathon Weakley[1,2,3], Andrew Townshend[1,3], Ryan G. Timmins[1,3], Matthew Morrison[1,3], Shaun J. McLaren[4,5]

Affiliations:
[1] School of Behavioural and Health Sciences, Australian Catholic University, Brisbane, Australia
[2] Carnegie Applied Rugby Research (CARR) Centre, Carnegie School of Sport, Leeds Beckett University, United Kingdom
[3] Sports Performance, Recovery, Injury and New Technologies (SPRINT) Research Centre, Australian Catholic University, Queensland, Australia
[4] Newcastle Falcons Rugby Club, Newcastle upon Tyne, United Kingdom
[5] Institute of Sport, Manchester Metropolitan University, Manchester, UK

ORCID Identifiers: Fraser Thurlow: 0000-0002-0234-9615
Jonathon Weakley: 0000-0001-7892-4885
Andrew D. Townshend: 0000-0002-6714-8304
Ryan G. Timmins: 0000-0003-4964-1848
Matthew Morrison: 0000-0002-3535-6707
Shaun J. McLaren: 0000-0003-0480-3209

Corresponding Author:

Fraser Thurlow
School of Behavioural and Health Sciences,
Australian Catholic University,
1100 Nudgee Road, Banyo 4014,
Queensland,
AUSTRALIA
E: fraser.thurlow@acu.edu.au

Supplementary Table S1. Modified Downs and Black scale outcomes for the assessment of reporting quality and risk of bias.

Study	Item number														Total score (out of 14)
	1	2	3	6	7	10	12	15	16	18	20	22	23	25	
Abt et al. [118]	1	1	0	1	1	1	0	0	1	1	1	0	0	1	9
AbuMoh'D et al. [180]	1	1	0	1	1	1	0	1	1	1	1	0	1	1	11
Aguiar et al. [95]	1	1	1	1	1	0	0	0	1	1	1	0	1	0	9
Akenhead et al. [57]	1	1	1	1	1	1	0	0	1	1	1	0	0	1	10
Alemdaroğlu et al. [23]	1	1	1	1	1	1	0	0	1	1	1	0	0	1	10
Almansba et al. [96]	1	1	1	1	1	0	0	0	1	1	1	0	0	1	9
Alizadeh et al. [167]	1	1	1	1	1	1	0	0	1	1	1	0	0	1	10
Altimari et al. [181]	1	1	1	1	1	0	0	0	1	1	1	0	0	1	9
Archiza et al. [182]	1	1	1	1	1	1	0	1	1	1	1	0	1	1	12
Attene et al. [115]	1	1	1	1	1	1	0	0	1	1	1	0	1	1	11
Ayarra et al. [183]	1	1	1	1	1	1	0	0	1	1	1	0	0	0	9
Aziz et al. [184]	1	1	1	1	1	0	0	0	1	1	1	0	0	0	8
Baldi et al. [185]	1	1	1	1	1	0	0	0	1	1	1	0	0	1	9
Balsalobre-Fernández et al. [186]	1	1	1	1	1	0	0	0	1	1	1	0	0	1	9
Beato et al. [187]	1	1	1	1	1	1	0	0	1	1	1	0	1	1	10
Beato et al. [188]	1	1	1	1	1	0	0	0	1	1	1	0	1	0	9
Beato & Drust [162]	1	1	1	1	1	1	0	0	1	1	1	0	0	1	9
Beaven et al. [189]	1	1	1	1	1	1	0	0	1	1	1	0	0	1	10
Binnie et al. [190]	1	1	1	1	1	1	0	0	1	1	1	0	1	1	11
Binnie et al. [191]	1	1	1	1	1	1	0	0	1	1	1	0	0	1	10
Binnie et al. [192]	1	1	1	1	1	1	0	0	1	1	1	0	0	1	10
Blasco-Lafarga et al. [108]	1	1	1	1	1	1	0	0	1	1	1	0	0	1	10
Borges et al. [193]	1	1	1	1	1	1	0	0	1	1	1	0	1	0	10
Brahim et al. [97]	1	1	1	1	1	1	0	0	1	1	1	0	0	1	10
Brini et al. [154]	1	1	0	1	1	0	0	0	1	1	1	0	0	1	8
Brini et al. [98]	1	1	1	1	1	1	0	0	1	1	1	0	0	1	10
Brini et al. [194]	1	1	1	1	1	0	0	0	1	1	1	0	1	0	9
Brini et al. [195]	1	1	1	1	1	1	0	0	1	1	1	0	0	1	10
Brini et al. [46]	1	1	1	1	1	1	0	0	1	1	1	0	0	1	10
Brocherie et al. [196]	1	1	1	1	1	1	0	0	1	1	1	0	0	1	10
Brocherie et al. [54]	1	1	1	1	1	0	0	0	1	1	1	0	0	1	9
Brocherie et al. [197]	1	1	1	1	1	1	0	1	1	1	1	0	1	1	12
Broderick et al. [141]	1	1	1	1	1	1	0	0	1	1	1	0	0	1	10
Buchheit [198]	1	1	1	1	1	1	0	0	1	1	1	0	0	1	10
Buchheit et al. [59]	1	1	1	1	1	1	0	0	1	1	1	0	0	1	10
Buchheit et al. [60]	1	1	1	1	1	0	0	0	1	1	1	0	0	1	9
Buchheit et al. [99]	1	1	1	1	1	0	0	0	1	1	1	0	0	1	9
Campa et al. [168]	1	1	1	1	1	1	0	0	1	1	1	0	0	1	10
Campos et al. [199]	1	1	1	1	1	0	0	0	1	1	1	0	0	1	9
Campos-Vazquez et al. [200]	1	1	1	1	1	1	0	0	1	1	1	0	1	1	10
Caprino et al. [201]	1	1	1	1	1	0	0	0	1	1	1	0	0	0	8
Castagna et al. [153]	1	1	1	1	1	1	0	0	1	1	1	0	0	1	10
Castagna et al. [202]	1	1	1	1	1	1	0	0	1	1	1	0	0	1	10
Chaouachi et al. [203]	1	1	1	1	1	1	0	0	1	1	1	0	0	0	9
Charlot et al. [204]	1	1	1	1	1	1	0	0	1	1	1	0	0	1	10
Chen et al. [205]	1	1	1	1	1	1	0	0	1	1	1	0	1	1	11
Clifford et al. [34]	1	1	1	1	1	1	0	0	1	1	1	0	1	1	11
Corrêa et al. [206]	1	1	1	1	1	0	0	0	1	1	1	0	0	0	8
Costello et al. [127]	1	1	1	1	1	1	0	0	1	1	1	0	0	1	10
Cuadrado-Peñafiel et al. [207]	1	1	1	1	1	0	0	0	1	1	1	0	0	0	8
Da Silva et al. [208]	1	1	1	1	1	0	0	0	1	1	1	0	0	0	8
Dal Pupo et al. [116]	1	1	1	1	1	1	0	0	1	1	1	0	0	1	10

Study	Item number														Total score (out of 14)
	1	2	3	6	7	10	12	15	16	18	20	22	23	25	
Dal Pupo et al. [209]	1	1	1	1	1	1	0	0	1	1	1	0	0	0	9
Daneshfar et al. [210]	1	1	1	1	1	0	0	0	1	1	1	0	0	1	9
Dardouri et al. [211]	1	1	1	1	1	1	0	0	1	1	1	0	0	1	10
de Andrade et al. [212]	1	1	1	1	1	1	0	0	1	1	1	0	0	1	10
Delextrat et al. [213]	1	1	1	1	1	1	0	0	1	1	1	0	1	1	11
Delextrat et al. [214]	1	1	1	1	1	1	1	0	1	1	1	0	0	1	11
Delextrat et al. [175]	1	1	1	1	1	1	0	0	1	1	1	0	0	1	10
Dellal et al. [61]	1	1	1	1	1	0	0	0	1	1	1	0	0	1	9
Dellal & Wong [100]	1	1	1	1	1	0	0	0	1	1	1	0	0	1	9
Dent et al. [131]	1	1	0	1	1	0	0	0	1	1	1	0	0	1	8
Donghi et al. [215]	1	1	1	1	1	0	0	0	1	1	1	0	0	1	9
Doyle et al. [216]	1	1	1	1	1	1	0	0	1	1	1	0	0	1	10
Dupont et al. [217]	1	1	1	1	1	0	0	0	1	1	1	0	0	1	9
Dupont et al. [86]	1	1	1	1	1	0	0	0	1	1	1	0	0	1	10
Eliakim et al. [124]	1	1	1	1	1	0	0	0	1	1	1	0	0	1	9
Elias et al. [218]	1	1	1	1	1	0	0	0	1	1	1	0	0	1	9
Elias et al. [219]	1	1	1	1	1	0	0	0	1	1	1	0	0	1	9
Eniseler et al. [220]	1	1	1	1	1	1	0	0	1	1	1	0	1	1	11
Eryilmaz & Kaynak [221]	1	1	1	1	1	1	0	0	1	1	1	0	0	0	9
Eryilmaz et al. [37]	1	1	1	1	1	0	0	0	1	1	1	0	0	0	8
Essid et al. [222]	1	1	1	1	1	1	0	0	1	1	1	0	0	1	10
Farjallah et al. [223]	1	1	1	1	1	0	0	0	1	1	1	0	0	1	9
Figueira et al. [119]	1	1	1	1	1	0	0	0	1	1	1	0	0	0	8
Fornasier-Santos et al. [224]	1	1	1	1	1	0	0	1	1	1	1	0	1	0	10
Fort-Vanmeerhaeghe et al.[225]	1	1	1	1	1	1	0	0	1	1	1	0	0	0	9
Fortin & Billaut [226]	1	1	1	1	1	0	0	1	1	1	1	0	0	1	10
Freitas et al. [227]	1	1	1	1	1	0	0	0	1	1	1	0	0	1	9
Gabbett [228]	1	1	0	1	1	1	0	0	1	1	1	0	0	0	9
Gabbett et al. [89]	1	1	0	1	1	1	0	0	1	1	1	0	0	0	8
Gabbett et al. [229]	1	1	1	1	1	1	0	0	1	1	1	0	0	0	9
Gabbett et al. [230]	1	1	0	1	1	1	0	0	1	1	1	0	0	0	9
Galvin et al. [231]	1	1	1	1	1	1	0	1	1	1	1	0	1	1	12
Galy et al. [177]	1	1	1	1	1	1	0	0	1	1	1	0	0	1	10
Gantois et al. [232]	1	1	0	1	1	0	0	0	1	1	1	0	0	0	7
Gantois et al. [14]	1	1	1	1	1	1	0	0	1	1	1	0	1	1	11
Gantois et al. [233]	1	1	1	1	1	1	0	0	1	1	1	0	0	1	10
García-Unanue et al. [169]	1	1	1	1	1	0	0	0	1	1	1	0	0	1	9
Gatterer et al. [234]	1	1	1	1	1	1	0	1	1	1	1	0	1	1	12
Gharbi et al. [83]	1	1	1	1	1	1	0	0	1	1	1	0	0	1	10
Gharbi et al. [235]	1	1	1	1	1	0	0	0	1	1	1	0	0	1	9
Gibson et al. [101]	1	1	1	1	1	1	0	0	1	1	1	0	1	0	10
Girard et al. [236]	1	1	1	1	1	1	0	0	1	1	1	0	0	1	10
Girard et al. [149]	1	1	1	1	1	1	0	0	1	1	1	0	0	1	10
González-Frutos et al. [237]	1	1	1	1	1	1	0	0	1	1	1	0	0	0	9
Gonzalo-skok et al. [102]	1	1	1	1	1	0	0	0	1	1	1	0	1	1	10
Goodall et al. [238]	1	1	1	1	1	1	0	0	1	1	1	0	0	1	10
Hamlin et al. [239]	0	1	1	1	1	0	0	0	1	1	1	0	1	1	9
Hamlin et al. [240]	1	1	1	1	1	0	0	1	1	1	1	0	1	1	11
Hammami et al. [241]	1	1	1	1	1	1	0	0	1	1	1	0	1	1	11
Haugen et al. [62]	1	1	1	1	1	1	0	0	1	1	1	0	1	1	11
Haugen et al. [128]	1	1	1	1	1	0	0	0	1	1	1	0	1	1	10
Hermassi et al. [242]	1	1	1	1	1	1	0	0	1	1	1	0	0	1	10
Higham et al. [90]	1	1	1	1	1	0	0	0	1	1	1	0	0	1	9
Hollville et al. [243]	1	1	1	1	1	1	0	0	1	1	1	0	0	0	9
Howatson et al. [35]	1	1	1	1	1	1	0	0	1	1	1	0	0	0	9

Study	Item number														Total score (out of 14)
	1	2	3	6	7	10	12	15	16	18	20	22	23	25	
Iaia et al. [120]	1	1	1	1	1	1	0	0	1	1	1	0	1	1	11
Iaia et al. [19]	1	1	1	1	1	1	0	0	1	1	1	0	1	1	11
Impellizzeri et al. [170]	1	1	1	1	1	1	0	0	1	1	1	0	0	1	10
Ingebrigtsen et al. [244]	1	1	1	1	1	0	0	0	1	1	1	0	0	1	9
Ingebrigtsen et al. [171]	1	1	1	1	1	0	0	0	1	1	1	0	0	1	9
Iacono et al. [42]	1	1	1	1	1	1	0	0	1	1	1	0	1	0	10
Izquierdo et al. [140]	1	1	1	1	1	0	0	1	1	1	1	0	1	1	11
Jang & Joo [245]	1	1	1	1	1	1	0	0	1	1	1	0	1	1	11
Jiménez-Reyes et al. [246]	1	1	1	1	1	0	0	0	1	1	1	0	0	1	9
Johnston & Gabbett [40]	1	1	1	1	1	1	0	0	1	1	1	0	0	1	10
Joo [110]	1	1	1	1	1	1	0	0	1	1	1	0	0	1	10
Jorge et al. [247]	1	1	1	1	1	0	0	0	1	1	1	0	0	1	9
Kaplan [109]	1	1	1	1	1	1	0	0	1	1	1	0	0	1	10
Keir et al. [25]	1	1	1	1	1	1	0	0	1	1	1	0	0	1	10
Keogh et al. [172]	1	1	1	1	1	1	0	0	1	1	1	0	0	0	9
Kilduff et al. [248]	1	1	1	1	1	1	0	0	1	1	1	0	0	1	10
Klatt et al. [36]	1	1	1	1	1	1	0	0	1	1	1	0	0	0	9
Krakan et al. [249]	1	1	0	1	1	1	0	0	1	1	1	0	0	0	8
Krueger et al. [250]	1	1	1	1	1	1	0	0	1	1	1	0	0	1	10
Lakomy et al. [78]	1	1	1	1	1	0	0	0	1	1	1	0	0	1	9
Lapointe et al. [251]	1	1	1	1	1	1	0	0	1	1	1	0	1	1	11
Le Rossignol et al. [173]	1	1	1	1	1	0	0	0	1	1	1	0	0	0	8
Little & Williams [121]	1	1	0	1	1	0	0	0	1	1	1	0	0	0	7
Lockie et al. [252]	1	1	1	1	1	1	0	0	1	1	1	0	0	0	9
Lockie et al. [253]	1	1	1	1	1	1	0	0	1	1	1	0	0	0	9
Lockie et al. [254]	1	1	1	1	1	1	0	0	1	1	1	0	0	0	9
Lombard et al. [255]	1	1	1	1	1	1	0	0	1	1	1	0	0	0	9
Madueno et al. [24]	1	1	1	1	1	1	0	0	1	1	1	0	0	1	10
Maggioni et al. [16]	1	1	1	1	1	1	0	0	1	1	1	0	1	0	10
Mancha-Triguero et al. [139]	1	1	1	1	1	1	0	0	1	1	1	0	0	0	9
Marcelino et al. [256]	1	1	1	1	1	0	0	0	1	1	1	0	0	1	9
Matzenbacher et al. [257]	1	1	1	1	1	0	0	0	1	1	1	0	0	1	9
McGawley & Andersson [258]	1	1	1	1	1	1	0	0	1	1	1	0	0	1	10
Meckel et al. [259]	1	1	0	1	1	0	0	0	1	1	1	0	0	1	8
Meckel et al. [260]	1	1	1	1	1	1	0	0	1	1	1	0	0	1	10
Meckel et al. [261]	1	1	1	1	1	1	0	0	1	1	1	0	0	1	10
Meckel et al. [262]	1	1	1	1	1	0	0	0	1	1	1	0	0	1	9
Meckel et al. [263]	1	1	1	1	1	0	0	0	1	1	1	0	0	1	9
Michalsik et al. [264]	1	1	1	1	1	0	0	0	1	1	1	0	0	1	9
Mohr et al. [265]	1	1	1	1	1	0	0	0	1	1	1	0	1	1	10
Mohr et al. [266]	1	1	1	1	1	0	0	0	1	1	1	0	1	1	10
Mohr et al. [267]	1	1	1	1	1	0	0	0	1	1	1	0	0	1	9
Moncef et al. [268]	1	1	1	1	1	0	0	0	1	1	1	0	0	0	8
Morcillo et al. [48]	1	1	1	1	1	0	0	0	1	1	1	0	0	1	9
Moreira et al. [269]	1	1	1	1	1	0	0	0	1	1	1	0	0	0	8
Mujika et al. [164]	1	1	1	1	1	0	0	0	1	1	1	0	0	1	9
Müller et al. [270]	1	1	0	1	1	1	0	0	1	1	1	0	0	0	8
Okuno et al. [271]	1	1	1	1	1	0	0	0	1	1	1	0	0	1	9
Nakamura et al. [272]	1	1	1	1	1	1	0	0	1	1	1	0	0	1	10
Nascimento et al [273]	1	1	1	1	1	1	0	0	1	1	1	0	1	0	10
Nedrehagen & Saeterbakken [274]	1	1	1	1	1	1	0	0	1	1	1	0	1	0	10
Nikolaidis et al. [275]	1	1	1	1	1	1	0	0	1	1	1	0	0	1	10
Padulo et al. [276]	1	1	1	1	1	1	0	0	1	1	1	0	0	1	10
Padulo et al. [277]	1	1	1	1	1	1	0	0	1	1	1	0	0	1	10
Padulo et al. [114]	1	1	1	1	1	1	0	0	1	1	1	0	0	1	10

Study	Item number														Total score (out of 14)
	**1**	**2**	**3**	**6**	**7**	**10**	**12**	**15**	**16**	**18**	**20**	**22**	**23**	**25**	
Padulo et al [156]	1	1	1	1	1	1	0	0	1	1	1	0	0	1	10
Padulo et al. [150]	1	1	1	1	1	1	0	0	1	1	1	0	0	1	10
Paulauskas et al. [122]	1	1	1	1	1	0	0	0	1	1	1	0	0	1	9
Perroni et al. [103]	1	1	1	1	1	1	0	0	1	1	1	0	0	0	9
Petisco et al. [278]	1	1	1	1	1	0	0	0	1	1	1	0	0	1	9
Purkhús et al. [279]	1	1	1	1	1	0	0	0	1	1	1	0	1	0	9
Pyne et al. [280]	1	1	1	1	1	0	0	0	1	1	1	0	0	0	8
Ramírez-Campillo et al. [281]	1	1	1	1	1	0	0	1	1	1	1	0	1	1	11
Rampinini et al. [282]	1	1	1	1	1	0	0	0	1	1	1	0	0	1	9
Rampinini et al. [174]	1	1	1	1	1	1	0	0	1	1	1	0	0	1	10
Rey et al. [283]	1	1	1	1	1	1	0	0	1	1	1	0	1	1	11
Rodríguez-Fernández et al. [165]	1	1	1	1	1	0	0	0	1	1	1	0	0	1	9
Rodríguez-Fernández et al. [284]	1	1	1	1	1	0	0	0	1	1	1	0	0	0	8
Røksund et al. [285]	1	1	1	1	1	1	0	0	1	1	1	0	0	1	10
Ruscello et al. [286]	1	1	1	1	1	1	0	0	1	1	1	0	1	1	11
Ruscello et al. [104]	1	1	1	1	1	1	0	0	1	1	1	0	1	1	11
Russell et al. [123]	1	1	1	1	1	1	0	0	1	1	1	0	0	1	10
Salleh et al. [287]	1	1	1	1	1	1	0	0	1	1	1	0	0	0	9
Sánchez-Sánchez et al. [117]	1	1	1	1	1	0	0	0	1	1	1	0	0	1	9
Sánchez-Sánchez et al. [288]	1	1	1	1	1	1	0	0	1	1	1	0	0	0	9
Sánchez-Sánchez et al. [289]	1	1	1	1	1	1	0	0	1	1	1	0	0	0	9
Sanders et al. [290]	1	1	1	1	1	1	0	0	1	1	0	0	0	0	8
Scanlan et al. [291]	1	1	1	1	1	1	0	0	1	1	1	0	0	0	9
Scanlan et al. [292]	1	1	1	1	1	1	0	0	1	1	1	0	0	0	9
Selmi et al. [58]	1	1	1	1	1	1	0	0	1	1	1	0	0	0	9
Selmi et al. [293]	1	1	1	1	1	1	0	0	1	1	1	0	1	1	11
Shalfawi et al. [294]	1	1	1	1	1	0	0	0	1	1	1	0	0	0	8
Shalfawi et al. [295]	1	1	1	1	1	0	0	0	1	1	1	0	1	0	9
Shalfawi et al. [296]	1	1	1	1	1	1	0	0	1	1	1	0	1	0	10
Silva et al. [297]	1	1	1	1	1	1	0	0	1	1	1	0	0	0	9
Soares-Caldeira et al. [298]	1	1	1	1	1	1	0	0	1	1	1	0	1	0	10
Spineti et al. [299]	1	1	1	1	1	1	0	0	1	1	1	0	1	0	10
Stojanovic et al. [300]	1	1	1	1	1	0	0	0	1	1	1	0	0	0	8
Suarez-Arrones et al. [105]	1	1	1	1	1	1	0	0	1	1	1	0	1	1	11
Taylor et al. [2]	1	1	1	1	1	1	0	0	1	1	1	0	0	1	10
Teixeira et al. [301]	1	1	1	1	1	1	0	0	1	1	1	0	1	1	11
Thomassen et al. [302]	1	1	1	1	1	1	0	0	1	1	1	0	1	1	11
Tønnessen et al. [303]	1	1	1	1	1	1	0	0	1	1	1	0	1	0	10
Torreblanca-Martinez et al. [304]	1	1	1	1	1	1	0	0	1	1	1	0	0	0	9
Tounsi et al. [176]	1	1	1	1	1	1	0	0	1	1	1	0	0	1	10
Trecroci et al. [305]	1	1	1	1	1	1	0	0	1	1	1	0	1	1	11
Turki et al. [111]	1	1	1	1	1	1	0	0	1	1	1	0	0	1	10
Ulupinar et al. [126]	1	1	1	1	1	1	0	0	1	1	1	0	0	1	10
Ulupinar et al. [125]	1	1	1	1	1	1	0	0	1	1	1	0	0	1	10
Van den Tillaar et al. [306]	1	1	1	1	1	1	0	0	1	1	1	0	0	0	9
Vasquez-Bonilla et al. [307]	1	1	1	1	1	1	0	0	1	1	1	0	0	1	10
Wadley & Le Rossignol [308]	1	1	1	1	1	0	0	0	1	1	1	0	0	0	8
West et al. [309]	1	1	1	1	1	1	0	0	1	1	1	0	0	1	10
Woolley et al. [33]	1	1	1	1	1	0	0	0	1	1	1	0	0	0	8
Yanci et al. [310]	1	1	1	1	1	0	0	0	1	1	1	0	1	0	9
Zagatto et al. [106]	1	1	1	1	1	1	0	0	1	1	1	0	0	1	10
Zagatto et al. [311]	1	1	1	1	1	1	0	0	1	1	1	0	1	1	11
Zagatto et al. [107]	1	1	1	1	1	1	0	0	1	1	1	0	0	0	9

Notes: 0 = no; 1 = yes; U = unable to determine. Item 1: clear aim/hypothesis; Item 2: outcome measures clearly described; Item 3: patient characteristics clearly described; Item 6: main findings clearly described; Item 7: measures of random variability provided; Item 10: actual probability values reported; Item 12: participants prepared to participate representative of the entire population; Item 15: blinding of outcome measures; Item 16: analysis completed was planned; Item 18: appropriate statistics; Item 20: valid and reliable outcome measures; Item 22: participants recruited over the same period; Item 23: randomised; Item 25: adjustment made for confounding variables.

Supplementary Table S2. Summary of participant and study characteristics from all included studies.

Study	Participants						Experimental Approach		
	N[#]	Sport	Level	Age (yrs)	Stature (cm)	Body mass (kg)	Design	Type	Details
Abt et al. [118]	11 (NR)	SOC	TRA	NR	NR	NR	NC	CRO (ran)	6 different, time-matched RS protocols (~60 s), performed twice each on an indoor synthetic sports floor, separated by 3–7 days.
AbuMoh'd et al. [180]	18	SOC	NAT	NR	NR	NR	C	PAG (r)	Baseline RS test on an athletics track, before an intervention.
Aguiar et al. [95]	34	SOC	NAT	INT: 27 ± 5<br>CON: 27 ± 5	INT: 175 ± 5<br>CON: 175 ± 6	INT: 73 ± 5<br>CON: 73 ± 7	NC	PAG (r)	RS test before an intervention
Akenhead et al. [57]	9	SOC	NAT	26 ± 3	172 ± 6	71 ± 7	NC	OBS	RS test performed in an indoor sports hall. Test ends when S_{dec} = 5% for 2 consecutive trials.
Alemdaroğlu et al. [23]	9	SOC	TRA	18 ± 1	177 ± 5	74 ± 7	NC	CRO (ran)	4 different RS tests performed twice each on an AG pitch, separated by 48 hrs.
Alizadeh et al. [167]	41	SOC	NAT	High: 17 ± 1<br>Med: 18 ± 1<br>Low: 17 ± 1	High: 177 ± 3<br>Med: 174 ± 5<br>Low: 171 ± 5	High: 71 ± 4<br>Med: 66 ± 5<br>Low: 67 ± 5	NC	OBS	Single RS test. Results according to the criterion of VO_{2max}
Almansba et al. [96]	17	SOC	NAT	16 ± 0	175 ± 1	67 ± 9	NC	CRO (ran)	2 RS tests performed on AG, separated by 72 hrs.
Altimari et al. [181]	46	SOC	NAT	18 ± 0	174 ± 5	64 ± 4	NC	OBS	RS test on a SOC field. U17 group only, birth tertiles combined.
Archiza et al. [182]	18 (0%)	SOC	NAT	Sham: 20 ± 2<br>INT: 22 ± 4	Sham: 160 ± 0<br>INT: 160 ± 0	Sham: 55 ± 5<br>INT: 56 ± 6	C	PAG (r)	Baseline RS test on a grass field, before an intervention.
Attene et al. [115]	36 (39%)	BB	NAT	M: 16 ± 1;<br>F: 16 ± 1	M: 178 ± 1<br>F: 165 ± 1	M: 66 ± 6<br>F: 56 ± 7	NC	PAG (r)	2 different baseline RS tests on an indoor court, as part of a testing battery, before a RST intervention.
Ayarra et al. [183]	40	FUT	TRA	22 ± 5	176 ± 7	70 ± 10	NC	OBS	Single RS test on an indoor wooden surface.
Aziz et al. [184]	40	MIX	INTL	23 ± 4	173 ± 1	64 ± 6	NC	OBS	RS test on NG, as part of a testing battery.
Baldi et al. [185]	26	SOC	NAT	23 ± 4	178 ± 6	72 ± 8	NC	OBS	RS test on outdoor NG, as part of a testing battery.
Balsalobre-Fernández et al. [186]	11	BB	NAT	25 ± 6	200 ± 11	99 ± 9	NC	OBS	RS test in an indoor hall.
Beato et al. [187]	36	SOC	TRA	21 ± 2	179 ± 7	74 ± 7	NC	PAG (r)	Baseline RS test before an intervention and RS training data

Study	Participants						Experimental Approach		
	N[#]	Sport	Level	Age (yrs)	Stature (cm)	Body mass (kg)	Design	Type	Details
Beato et al. [188]	20	SOC	NAT	18–21	177 ± 6	71 ± 7	NC	PAG (r)	Baseline RS test before an intervention and RS training data
Beato & Drust [162]	16	SOC	TRA	21 ± 1	179 ± 8	71 ± 8	NC	CRO (ran)	RS test on a synthetic outdoor track. Sub-maximal RS test excluded from the review.
Beaven et al. [189]	12	RUG	NAT	22 ± 1	185 ± 4	96 ± 9	C	CRO (ran)	RS test on an indoor running track.
Binnie et al. [190]	24 (0%)	HOC	NR	SAN: 19 ± 7<br>GRA: 21 ± 4	SAN: 168 ± 12<br>GRA: 167 ± 67	SAN: 66 ± 9<br>GRA: 63 ± 6	NC	PAG (r)	Baseline RS test in a gymnasium, before an intervention. Participant's pair-matched by VO_{2max}.
Binnie et al. [191]	10 (70%)	HOC/ NET	NAT	M: 23 ± 3<br>F: 20 ± 3	M: 182 ± 5<br>F: 176 ± 11	M: 83 ± 6<br>F: 69 ± 15	NC	CRO (ran)	Baseline RS test in a gymnasium.
Binnie et al. [192]	10 (80%)	HOC/ NET	NR	M: 22 ± 2<br>F: 21 ± 1	M; 181 ± 5<br>F: 179 ± 14	M: 78 ± 6<br>F: 74 ± 18	NC	CRO (ran)	Baseline RS test in a gymnasium
Blasco-Lafarga et al. [108]	13	SOC	NAT	18 ± 1	172 ± 4	68 ± 6	NC	CRO	RS test on a SOC pitch.
Borges et al. [193]	20	SOC	NAT	17 ± 1	175 ± 7	69 ± 9	NC	PAG (r)	Baseline RS test before an intervention.
Brahim et al. [97]	27	SOC	NAT	DEF: 18 ± 1<br>MID: 18 ± 1<br>FWD: 17 ± 1	DEF: 183 ± 6<br>MID: 178 ± 5<br>FWD: 180 ± 5	DEF: 75 ± 9<br>MID: 70 ± 7<br>FWD: 72 ± 4	NC	OBS	3 different RS tests on NG, separated by > 1 day.
Brini et al. [154]	16	BB	NR	23 ± 3	186 ± 10	78 ± 8	NC	CRO (ran)	4 different RS protocols, separated by 48-hrs.
Brini et al. [98]	16	BB	NAT	22 ± 3	186 ± 10	78 ± 8	C	PAG (r)	RS test before an intervention.
Brini et al. [194]	16	BB	NR	23 ± 2	186 ± 9	78 ± 11	C	PAG (r)	RS test before an intervention.
Brini et al. [195]	16	BB	NAT	23 ± 2	186 ± 10	78 ± 8	NC	CRO (ran)	2 different RS tests on a BB court, separated by > 48-hrs.
Brini et al. [46]	40	BB	NAT	27 ± 3	192 ± 9	88 ± 9	NC	OBS	RS test on a wooden BB court.
Brocherie et al. [196]	16	SOC	INTL	27 ± 4	177 ± 4	72 ± 5	NC	OBS	RS test on indoor AG, as part of a testing battery.
Brocherie et al. [54]	8	SOC	INTL	28 ± 5	176 ± 4	72 ± 3	NC	OBS	RS test on indoor AG.

Study	Participants						Experimental Approach		
	N#	Sport	Level	Age (yrs)	Stature (cm)	Body mass (kg)	Design	Type	Details
Brocherie et al. [197]	36	HOC	NAT	25 ± 5	178 ± 6	76 ± 8	C	PAG (r)	Baseline RS test on an indoor synthetic floor, before an intervention.
Broderick et al. [141]	19	MIX	TRA	21.0 ± 2.0	178.8 ± 7.2	8.1 ± 8.9	C	CRO (ran)	RS tests in an indoor gymnasium, separated by 7 days.
Buchheit [198]	27	MIX	NAT	HB: 23 ± 3<br>TS3: 23 ± 4	HB: 188 ± 7<br>TS3: 180 ± 8	HB: 88 ± 11<br>TS3: 77 ± 9	NC	OBS	RS tests were performed by different groups of athletes on an indoor synthetic track.
Buchheit et al. [59]	13	MIX	NR	22 ± 3	179 ± 5	75 ± 5	NC	CRO (ran)	4 different RS protocols on an indoor synthetic track, separated by > 48-hrs.
Buchheit et al. [60]	13	MIX	NR	22 ± 3	179 ± 5	75 ± 5	NC	CRO (ran)	2 different RS protocols on an indoor synthetic track, separated by > 48-hrs.
Buchheit et al. [99]	12	MIX	NAT	22 ± 2	178 ± 8	76 ± 4	NC	CRO (ran)	4 different RS protocols on an indoor synthetic track, separated by > 48-hrs.
Campa et al. [168]	36	SOC	NAT	17 ± 1	EL: 177 ± 6<br>S-EL: 178 ± 6	EL: 69 ± 4<br>S-EL: 70 ± 7	NC	OBS	RS test on NG.
Campos et al. [199]	11	FUT	NAT	19 ± 1	178 ± 7	71 ± 6	NC	PAG	Baseline RS test on an indoor FUT court before an intervention.
Campos-Vazquez et al. [200]	21	SOC	NAT	18 ± 1	177 ± 6	70 ± 7	NC	PAG (r)	Baseline RS test on AG, before an intervention.
Caprino et al. [201]	10	BB	TRA	16 ± 1	184 + 7	77 + 8	NC	OBS	RS test before an official BB match.
Castagna et al. [153]	16	BB	TRA	17 ± 1	181 ± 6	73 ± 10	NC	OBS (ran)	2 different RS tests on an indoor wooden BB court, separated by > 48-hrs, as part of a testing battery.
Castagna et al. [202]	18	BB	TRA	17 ± 1	181 ± 6	73 ± 10	NC	OBS	RS test on an indoor wooden BB court, separated by > 48-hrs.
Chaouachi et al. [203]	23	SOC	NAT	19 ± 1	181 ± 6	73 ± 4	NC	CRO (ran)	RS test on an indoor synthetic track.
Charlot et al. [204]	10	FUT	NAT	26 ± 4	170 ± 7	70 ± 9	NC	OBS*	RS test before a FUT tournament
Chen et al. [205]	26	SOC	NAT	21 ± 1	173 ± 4	65 ± 5	C	PAG (r)	RS test on an indoor synthetic surface.
Clifford et al. [34]	20	MIX	NAT	CON: 21 ± 2<br>INT: 23 ± 3	CON: 177 ± 1<br>INT: 183 ± 1	CON: 73 ± 12<br>INT: 77 ± 10	C	PAG (r)	Baseline RS test before an intervention period.

Study	Participants						Experimental Approach		
	N[#]	Sport	Level	Age (yrs)	Stature (cm)	Body mass (kg)	Design	Type	Details
Corrêa et al. [206]	10	SOC	TRA	19 ± 1	179 ± 0	71 ± 7	NC	OBS*	Baseline RS test on outdoor NG.
Costello et al. [127]	24	RUG	NAT	21 ± 2	182 ± 5	88 ± 9	C	CRO (ran)	RS protocol (session 1 & day 1 only)
Cuadrado-Peñafiel et al. [207]	37	SOC/ FUT	NAT	SOC: 29 ± 1<br>FUT: 27 ± 5	SOC: 178 ± 1<br>FUT: 179 ± 1	SOC: 73 ± 12<br>FUT: 75 ± 7	NC	OBS	Single RS test
Da Silva et al. [208]	29	SOC	NAT	18 ± 1	179 ± 5	74 ± 7	NC	OBS	Single RS test.
Dal Pupo [116]	14	FUT	TRA	U17	170 ± 6	63 ± 8	NC	OBS (ran)	2 different RS tests on a FUT court, separated by 48-hrs.
Dal Pupo et al. [209]	7	FUT	TRA	16 ± 1	172 ± 9	65 ± 8	NC	OBS (ran)	2 different RS tests on a concrete floor, separated by 48-hrs.
Daneshfar et al. [210]	20	HB	INTL	16 ± 1	185 ± 5	83 ± 6	NC	OBS (ran)	2 different RS tests were performed indoors, separated by 48-hrs, as part of a testing battery.
Dardouri et al. [211]	29	MIX	NR	23 ± 2	180 ± 10	69 ± 9	NC	OBS	RS test, indoors, as part of a testing battery.
de Andrade et al. [212]	16	MIX	NAT	22 ± 3	186 ± 10	79 ± 23	NC	OBS	Single RS test on an indoor rigid surface.
Delextrat et al. [213]	17 (53%)	BB	TRA	M: 22 ± 3<br>F: 21 ± 3	M: 19 ± 9<br>F: 176 ± 8	M: 91 ± 10<br>F: 74 ± 10	C	CRO (ran) (r)	Baseline RS test, before an intervention.
Delextrat et al. [214]	31	BB	TRA	FWD: 16 ± 1<br>G: 17 ± 1<br>CEN: 16 ± 1	FWD: 183 ± 5<br>G: 175 ± 6<br>CEN: 191 ± 8	FWD: 75 ± 7<br>G: 69 ± 5<br>CEN: 81 ± 3	NC	OBS (ran)	RS test, as part of a testing battery.
Delextrat et al. [175]	16 (50%)	BB	TRA	M: 23 ± 3<br>F: 22 ± 2	M: 191 ± 9<br>F: 179 ± 9	M: 90 ± 10<br>F: 78 ± 9	C	PAG (ran) (r)	Baseline RS test, before an intervention.
Dellal et al. [61]	22	SOC	INTL	24 ± 4	178 ± 6	80 ± 6	NC	OBS	3 different RS protocols performed indoors, separated by > 48-hrs, as part of a testing battery.
Dellal & Wong [100]	39	SOC	NAT	Open age to U17	PRO: 180 ± 4<br>U19: 178 ± 7<br>U17: 180 ± 6	PRO: 72 ± 4<br>U19: 69 ± 6<br>U17: 67 ± 5	NC	OBS	2 different RS tests on AG, separated by 1 week.
Dent et al. [131]	15 (47%)	SOC	TRA	M: 20 ± 2<br>F: 19 ± 2	NR	M: 79 ± 11<br>F: 62 ± 7	NC	CRO	Single RS protocol.

Study	Participants						Experimental Approach		
	N[#]	Sport	Level	Age (yrs)	Stature (cm)	Body mass (kg)	Design	Type	Details
Donghi et al. [215]	12	SOC	NAT	17 ± 1	178 ± 6	69 ± 4	C	CRO (ran)	Baseline RS test in an indoor gym,
Doyle et al. [216]	25 (0%)	SOC	INTL	19 ± 3	167 ± 6	63 ± 7	NC	OBS	RS test performed on an indoor surface.
Dupont et al. [217]	12	SOC	TRA	23 ± 4	179 ± 6	72 ± 7	NC	OBS	RS test on an indoor tartan track.
Dupont et al. [86]	11	SOC	TRA	25 ± 4	176 ± 6	68 ± 4	NC	OBS	RS test on an indoor tartan track.
Eliakim et al. [124]	12	BB	NAT	16 ± 1	186 ± 10	76 ± 6	C	CRO (ran)	RS test on a BB court, CON condition only.
Elias et al. [218]	14	ARF	NAT	21 ± 3	186 ± 7	80 ± 7	NC	CRO (ran)	Baseline RS test on an indoor, wooden surface.
Elias et al. [219]	24	ARF	NAT	20 ± 3	186 ± 6	81 ± 8	NC	PAG (r)	Baseline RS test on an indoor wooden sprung floor, before an intervention.
Eniseler et al. [220]	19	SOC	NAT	17 ± 1	174 ± 5	66 ± 6	C	PAG (r)	Baseline RS test on NG before an intervention
Eryilmaz & Kaynak [221]	16	VB	TRA	21 ± 1	184 ± 5	74 ± 8	NC	OBS	RS test on an indoor VB court.
Eryilmaz et al. [37]	12	MIX	TRA	24 ± 4	179 ± 6	73 ± 9	NC	SG	Data extracted from one session during a RST intervention.
Essid et al. [222]	18	HB	NAT	17 ± 0.3	190 ± 10	78 ± 10	NC	CRO (ran)	RS test (morning session only)
Farjallah et al. [223]	20	SOC	NAT	19 ± 1	180 ± 10	70 ± 11	C	PAG	RS test on a SOC field, before an intervention.
Figueira et al. [119]	12	BB	NAT	21 ± 2	190 ± 7	86 ± 6	NC	CRO (ran)	2 different RS tests.
Fornasier-Santos et al. [224]	35	RUG	NAT	18 ± 1	182 ± 7	95 ± 15	C	PAG (r)	Baseline RS test on an indoor, concrete floor and RS training data from the control group.
Fort-Vanmeerhaeghe et al.[225]	11	HB (0%)	NAT	17 ± 1	182 ± 7	70 ± 8	NC	OBS	RS test on a BB court
Fortin & Billaut [226]	15	AF	TRA	21 ± 2	188 ± 19	82 ± 3	NC	PAG	Baseline RS test before an intervention.

Study	Participants						Experimental Approach		
	N[#]	Sport	Level	Age (yrs)	Stature (cm)	Body mass (kg)	Design	Type	Details
Freitas et al. [227]	9	BB	NAT	21 ± 3	198 ± 8	93 ± 15	NC	CRO (r)	Baseline RS test in an indoor centre.
Gabbett [228]	19 (0%)	SOC	NAT / INTL	18 ± 3	NR	NR	NC	OBS	Same RS test, repeated twice.
Gabbett et al. [89]	58	RUG	NAT	24 ± 4	184 ± 6	97 ± 10	NC	OBS	RS test on a synthetic surface, as part of a testing battery.
Gabbett et al. [229]	86	RUG	NAT	ST: 25 ± 4<br>N-ST: 23 ± 4<br>N-SEL: 22 ± 4	ST: 185 ± 5<br>N-ST: 182 ± 6<br>N-SEL: 183 ± 7	ST: 96 ± 8<br>N-ST: 99 ± 12<br>N-SEL: 96 ± 11	NC	OBS	RS test on a synthetic surface, as part of a testing battery.
Gabbett et al. [230]	16 (0%)	SOC	NAT / INTL	18.3 ± 2.8	NR	NR	NC	PAG	Baseline RS test, before an intervention.
Galvin et al. [231]	42	RUG	NAT	18 ± 2	183 ± 7	88 ± 9	C	PAG (r)	RS test performed outdoors, before an intervention.
Galy et al. [177]	22	FUT	INTL	MG: 24 ± 4<br>N-MG: 23 ± 5	MG: 173 ± 5 | N-MG: 180 ± 8	MG: 72 ± 7<br>N-MG: 74 ± 12	NC	OBS	RS test on an indoor synthetic court, as part of a testing battery.
Gantois et al. [232]	20	BB	NAT	18-24	180 ± 6	81 ± 13	NC	OBS	RS test on a BB court.
Gantois et al. [14]	20	BB	NAT	21 ± 2	181 ± 8	74 ± 9	C	PAG (r)	RS test on a BB court, before an intervention.
Gantois et al. [233]	12	BB	NAT	22 ± 3	180 ± 2	81 ± 14	NC	SG	Baseline RS test, before an intervention.
García-Unanue et al. [169]	33	FUT	NAT / TRA	23 ± 4	176 ± 6	73 ± 6	NC	OBS	RS test on a FUT field. Results according to playing level.
Gatterer et al. [234]	14	SOC	TRA	24 ± 2	178 ± 7	77 ± 7	C	PAG	Baseline RS test, before an intervention.
Gharbi et al. [83]	20	MIX	TRA	22 ± 3	178 ± 7	71 ± 8	NC	CRO (ran)	Series of RS protocols on an indoor synthetic surface, separated by >24 hrs.
Gharbi et al. [235]	16	MIX	TRA	23 ± 2	178 ± 4	72 ± 3	C	OBS (ran)	RS test on an indoor synthetic surface
Gibson et al. [101]	32	SOC	TRA	18 ± 1	179 ± 5	177 ± 5	NC	OBS	RS test on an indoor synthetic surface

Study	Participants						Experimental Approach		
	N[#]	Sport	Level	Age (yrs)	Stature (cm)	Body mass (kg)	Design	Type	Details
Girard et al. [236]	12	SOC	INTL	28 ± 5	176 ± 4	64 ± 5	NC	OBS	RS test on indoor AG, wearing normal football boots with plantar pressure insoles inserted.
Girard et al. [149]	13	SOC	NAT	18 ± 1	190 ± 10	83 ± 10	NC	OBS	RS test on indoor AG, wearing normal football boots with plantar pressure insoles inserted.
González-Frutos et al. [237]	13 (0%)	HOC	INTL	25 ± 6	167 ± 4	59 ± 4	NC	OBS	Single RS test
Gonzalo-skok et al. [102]	22	BB	NAT	16 ± 1	180 ± 6	81 ± 13	C	PAG (r)	2 different RS tests were performed on an indoor BB court, as part of a testing battery, before an intervention.
Goodall et al. [238]	12	MIX	NR	25 ± 6	180 ± 7	77 ± 7	NC	OBS	Single RS protocol.
Hamlin et al. [239]	20 (85%)	RUG	NAT	19 ± 1	180 ± 10	85 ± 14	NC	CRO (r)	Baseline RS protocol, before an intervention.
Hamlin et al. [240]	19	RUG	TRA	CON: 22 ± 4<br>INT: 20 ± 2	CON: 178 ± 5<br>INT: 174 ± 5	CON: 88 ± 14<br>INT: 77 ± 10	C	PAG (r)	Baseline RS test in an indoor stadium, on 2 separate occasions, 4–5 days apart.
Hammami et al. [241]	28	HB	NAT	INT: 17 ± 0<br>CON: 17 ± 0	INT: 163 ± 4<br>CON: 164 ± 4	INT: 61 ± 5<br>CON: 60 ± 4	C	PAG (r)	Baseline RS test before an intervention
Haugen et al. [62]	25 (52%)	SOC	TRA	INT: 17 ± 1<br>CON: 17 ± 1	INT: 174 ± 8<br>CON: 173 ± 6	INT: 65 ± 8<br>CON: 62 ± 7	C	PAG (r)	Baseline RS test before an intervention
Haugen et al. [128]	42	SOC	TRA	17 ± 1	178 ± 6	66 ± 9	C	PAG (r)	Baseline RS test before an intervention
Hermassi et al. [242]	22	HB	NAT	19 ± 0	179 ± 2	83 ± 1	NC	OBS (ran)	2 different RS tests, separated by 3–7 days, as part of a testing battery.
Higham et al. [90]	18	RUG	INTL	22 ± 2	183 ± 6	90 ± 8	NC	OBS	RS test on an indoor synthetic track, as part of a testing battery.
Hollville et al. [243]	10	HOC	NAT	19 ± 1	180 ± 6	72 ± 5	NC	OBS	RS test on AG. Results from the 1st set only.
Howatson et al. [35]	20	MIX	NAT	22 ± 2	178 ± 7	85 ± 14	NC	OBS	Single RS protocol performed on an outdoor track.
Iaia et al. [120]	18	SOC	NAT	19 ± 1	180 ± 7	74 ± 7	NC	PAG (r)	Baseline RS test on AG, as part of a testing battery, performed by 2 different groups.

Study	Participants						Experimental Approach		
	N#	Sport	Level	Age (yrs)	Stature (cm)	Body mass (kg)	Design	Type	Details
Iaia et al. [19]	29	SOC	NAT	17 ± 1	178 ± 10	69 ± 8	C	PAG (r)	Data extracted from baseline RS tests on AG and the 1st RS training session of an intervention.
Impellizzeri et al. [170]	22	SOC	NAT	22 ± 1	177 ± 4	73 ± 5	NC	OBS	Same RS test on NG, performed twice on different occasions
Impellizzeri et al. [170]	30	SOC	NAT	25 ± 5	181 ± 5	78 ± 8	NC	OBS*	RS test on NG, performed at different timepoints across a regular season.
Impellizzeri et al. [170]	108	SOC	NAT / TRA	24 ± 4	75 ± 7	179 ± 5	NC	OBS*	RS test on NG. Results according to player level.
Ingebrigtsen et al. [244]	57	SOC	NAT	22 ± 5	181 ± 5	75.2 ± 7.6	NC	OBS	RS test on indoor AG, as part of a testing battery
Ingebrigtsen et al. [171]	51	SOC	NAT	PRO: 26 ± 7<br>SEMI: 20 ± 3	PRO: 183 ± 5<br>SEMI: 181 ± 5	NR	NC	OBS	RS test. Results according to player level.
Iacono et al. [42]	18	HB	NAT	25 ± 4	188 ± 7	91 ± 9	NC	PAG (r)	RS test on an indoor court before an intervention
Izquierdo et al. [140]	19	HB	NAT	INT: 21 ± 5<br>PLA: 24 ± 5	INT: 182 ± 8<br>PLA: 190 ± 8	INT: 79 ± 8<br>PLA: 87 ± 12	C	PAG (r)	Baseline RS test on an indoor HB court, before an intervention.
Jang & Joo [245]	12	SOC	NAT	23 ± 2	175 ± 6	71 ± 5	NC	CRO (r)	Single RS test.
Jiménez-Reyes et al. [246]	20	RUG	INTL	24 ± 4	188 ± 5	96 ± 7	NC	OBS	RS test on an indoor synthetic athletics track.
Johnston & Gabbett [40]	12	RUG	NR	23 ± 2	179 ± 10	85 ± 11	NC	CRO (ran)	The same RS test was performed twice on different occasions.
Joo [140]	11	SOC	TRA	22 ± 2	174 ± 6	NR	NC	SG	Baseline RS test before an intervention.
Jorge et al. [247]	43	SOC	NAT	18 ± 1	178 ± 8	74 ± 10	NC	OBS*	RS test on NG, performed at different timepoints across a season.
Kaplan [109]	85	SOC	TRA	21 ± 3.8	176 ± 6	69 ± 7	NC	OBS	RS test on NG as part of a testing battery.
Keir et al. [25]	8	SOC	NAT	21 ± 2	176 ± 5	75 ± 4	NC	OBS (ran)	Single RS test.

Study	Participants						Experimental Approach		
	N#	Sport	Level	Age (yrs)	Stature (cm)	Body mass (kg)	Design	Type	Details
Keogh [172]	74 (0%)	HOC	TRA	REP: 19 ± 1<br>Club: 20 ± 2	REP: 165 ± 1<br>Club: 164 ± 1	REP: 59 ± 1<br>Club: 57 ± 1	NC	OBS	RS test as part of a testing battery
Kilduff et al. [248]	20	RUG	NAT	26 ± 2	185 ± 4	96 ± 8	C	CRO (ran)	Baseline RS test on an indoor synthetic track, before an intervention.
Klatt et al. [36]	29	HB	NAT	U20: 18 ± 1<br>SEN: 27 ± 6	U20: 182 ± 8<br>SEN: 192 ± 9	U20: 79 ± 9<br>SEN 90 ± 14	NC	OBS*	Single RS protocol
Krakan et al. [249]	41 (NR)	MIX	TRA	NR	RS-G, 181 ± 7<br>PLY, 175 ± 6	RS-G, 81 ± 8<br>PLY, 77 ± 9	NC	PAG	RS test before an intervention
Krueger et al. [250]	18	HOC	INTL	17 ± 1	182 ± 6	74 ± 8	C	PAG (r)	Baseline RS test, before an intervention.
Lakomy et al. [78]	18	HOC	NAT	24 ± 4	179 ± 5	77 ± 4	C	CRO (ran) (r)	2 different RS protocols on AG
Lapointe et al. [251]	17 (71%)	BB	NAT	22	186 ± 12	89 ± 17	C	PAG (r)	Baseline RS test before an intervention.
Le Rossignol et al. [173]	20	ARF	NAT	22 ± 2	188 ± 6	88 ± 8	NC	OBS	RS test on an outdoor synthetic track, as part of a testing battery
Little & Williams [121]	6	SOC	NAT	18–27	NR	NR	NC	CRO (ran)	4 different RS protocols, performed on non-consecutive days.
Lockie et al. [252]	17	SOC	INTL	20 ± 2	181 ± 6	78 ± 7	NC	OBS	RS test on outdoor NG, as part of a testing battery.
Lockie et al. [253]	19 (0%)	SOC	INTL	20 ± 1	164 ± 6	61 ± 8	NC	OBS	RS test on outdoor NG, as part of a testing battery.
Lockie et al. [254]	18	SOC	INTL	21 ± 2	181 ± 6	78 ± 6	NC	OBS	RS test on outdoor NG, as part of a testing battery. Results are for all players.
Lombard et al. [255]	23	HOC	NAT / INTL	24 ± 3	178 ± 3	77 ± 5	NC	OBS	RS test on AG, as part of a testing battery. Results are for all players.
Madueno et al. [24]	8 (75%)	BB	NAT	20 ± 2	183 ± 10	78 ± 17	NC	CRO (ran)	2 different RS protocols on an indoor hardwood floor, separated by 2–7 days.
Maggioni et al. [16]	36	BB	NAT	19 ± 1	182 ± 7	74 ± 10	C	PAG (r)	RS training data from an intervention.
Mancha-Triguero et al. [139]	61	BB	NAT	U18	M: 195<br>F: 168	M: 85<br>F: 57	NC	OBS	RS test on BB court.

Study	Participants						Experimental Approach		
	N#	Sport	Level	Age (yrs)	Stature (cm)	Body mass (kg)	Design	Type	Details
Marcelino et al. [256]	12	BB	TRA	19 ± 1	193 ± 7	89 ± 15	NC	CRO	Same 2 baseline RS tests, separated by 24-hrs, before an intervention.
Matzenbacher et al. [152]	9	FUT	TRA	17 ± 0	176 ± 7	68 ± 9	NC	OBS *	RS test performed at the beginning and end of the season.
McGawley & Andersson [258]	18	SOC	NAT	23 ± 4	180 ± 8	76 ± 6	NC	PAG	Baseline RS test on AG, before an intervention.
Meckel et al. [259]	18	SOC	NAT	22-32	NR	77 ± 8	NC	OBS *	RS test performed at different timepoints across a season.
Meckel et al. [260]	12	BB	NAT	17 ± 1	187 ± 9	78 ± 6	NC	CRO (ran)	RS test on a BB court, after a game day warm-up.
Meckel et al. [261]	33	SOC	NAT	16-18	175 ± 4	67 ± 7	NC	OBS (ran)	2 different RS tests on NG, separated by ~1 week, as part of a testing battery.
Meckel et al. [262]	16	VB	NAT	26 ± 5	192 ± 6	84 ± 7	NC	OBS (ran)	RS test in a sports arena, as part of a testing battery.
Meckel et al. [263]	20	SOC	NAT	17 ± 1	174 ± 7	67 ± 7	NC	CRO (ran)	RS test on a SOC pitch, after a match warm up.
Michalsik et al.[264]	26	HB	INTL	26 ± 3	189 ± 6	91 ± 9	NC	OBS	RS test on an indoor HB court. Results are all players combined.
Mohr et al. [265]	40	SOC	NAT	22 ± 0	177 ± 1	73 ± 1	C	PAG (r)	Baseline RS test on NG, before an intervention.
Mohr et al. [266]	18	SOC	TRA	19 ± 1	179 ± 6	79 ± 4	NC	PAG (r)	Baseline RS test on AG, before an intervention.
Mohr et al. [267]	17	SOC	NAT	27 ± 1	184 ± 1	80 ± 2	C	CRO	Baseline RS test on indoor AG, before an intervention.
Moncef et al. [268]	44	HB	NAT	22 ± 3	182 ± 6	85 ± 2	NC	OBS	RS test, as part of a testing battery.
Morcillo et al. [48]	18	SOC	NAT	27 ± 4	180 ± 5	78 ± 5	NC	OBS	Single RS test.
Moreira et al. [269]	10	FUT	NAT	24 ± 3	174 ± 5	73 ± 9	C	CRO (ran)	Baseline RS test before an intervention.
Mujika et al. [164]	28	SOC	TRA	U17 & U18	U17: 178 ± 6<br>U18: 179 ± 9	U17: 70 ± 7<br>U18: 72 ± 8	NC	OBS	RS test on indoor AG.
Müller et al. [270]	12	RUG	TRA	25 ± 4	177 ± 5	92 ± 12	NC	CRO (ran)	Single RS test.

Study	Participants						Experimental Approach		
	N#	Sport	Level	Age (yrs)	Stature (cm)	Body mass (kg)	Design	Type	Details
Nakamura et al. [272]	13	HB	NAT	24 ± 4	187 ± 7	88 ± 3	NC	OBS	RS test in a gymnasium.
Nascimento et al [273]	18	FUT	TRA	17 ± 1	177 ± 5	69 ± 7	C	PAG (r)	Baseline RS test before a long-term RS intervention.
Nedrehagen &Saeterbakken [274]	22 (41%)	SOC	TRA	INT: 20 ± 3<br>CON: 22 ± 3	INT: 20 ± 3<br>CON: 22 ± 3	69 ± 10	C	PAG (r)	Baseline RS test on indoor AG before an intervention
Nikolaidis et al. [275]	36	SOC	TRA	22 ± 5	180 ± 6	75 ± 8	NC	OBS	RS test on AG, as part of a testing battery.
Okuno et al. [271]	12	HB	NAT	19 ± 2	185 ± 87	85 ± 10	NC	CRO	Single RS test
Padulo et al. [276]	18	SOC	NAT	16 ± 0	174 ± 10	65 ± 10	NC	CRO (ran)	Same RS test, repeated twice, on AG, separated by > 6 days.
Padulo et al. [277]	17	SOC	NAT	17 ± 1	179 ± 5	69 ± 7	NC	CRO (ran)	Same 2 RS tests and 1 different RS test on AG, separated by 3 days.
Padulo et al. [114]	18	BB	NAT	16 ± 1	178 ± 10	66 ± 9	NC	CRO	2 different RS tests on an indoor BB court, repeated twice, separated by > 48-hrs, as part of a testing battery.
Padulo et al [156]	18	SOC	NAT	16 ± 0	174 ± 10	65 ± 10	NC	CRO	The same RS test was repeated twice, and 1 different RS test, on AG, separated by 1-week.
Padulo et al. [150]	17	SOC	INTL	16 ± 0	181 ± 10	66 ± 10	NC	CRO	3 different RS tests on AG, separated by 5 days.
Paulauskas et al. [122]	12	BB	NAT	21 ± 2	190 ± 7	86 ± 6	NC	CRO (ran)	2 different RS protocols, on an indoor wooden BB court, separated by 1-week.
Perroni et al. [103]	12	SOC	TRA	23 ± 6	177 ± 6	75 ± 7	NC	SG	Baseline RS test on AG, before an intervention.
Petisco et al. [278]	10	SOC	NAT	22 ± 3	178 ± 4	70 ± 3	C	CRO (ran)	RS test following the regular warm-up protocol.
Purkhús et al. [279]	25 (0%)	VB	NAT	18 ± 4	172 ± 7	63 ± 11	C	PAG (r)	Baseline RS test on an indoor HB court, before an intervention.
Pyne et al. [280]	60	ARF	NAT	18 ± 0	188 ± 7	82 ± 8	NC	OBS	RS test on an indoor sprung wooden floor, as part of a testing battery.
Ramírez-Campillo et al. [281]	30 (0%)	SOC	TRA	CON: 23 ± 2<br>PLA: 23 ± 2<br>INT: 23 ± 3	CON: 161 ± 6<br>PLA: 164 ± 9<br>CR: 162 ± 4	CON: 60 ± 8<br>PLA: 57 ± 5<br>INT: 60 ± 8	C	PAG (r)	Baseline RS test, before an intervention.
Rampinini et al. [282]	18	SOC	NAT	26 ± 5	182 ± 4	81 ± 8	NC	OBS	RS test on outdoor NG.

Study	Participants						Experimental Approach		
	N[#]	Sport	Level	Age (yrs)	Stature (cm)	Body mass (kg)	Design	Type	Details
Rampinini et al. [174]	23	SOC	NAT / TRA	PRO: 25 ± 4<br>AM: 26 ± 6	PRO: 180 ± 3<br>AM: 177 ± 5	PRO: 74 ± 5<br>AM: 71 ± 8	NC	OBS	RS test on outdoor NG.
Rey et al. [283]	19	SOC	TRA	INT: 24 ± 3<br>CON: 24 ± 2	INT: 179 ± 5<br>CON: 178 ± 5	INT: 74 ± 7<br>CON: 75 ± 7	C	PAG (r)	Baseline RS test on an indoor court, before an intervention.
Rodríguez-Fernández et al. [165]	33	SOC	NAT	PRO: 24 ± 3<br>YTH: 18 ± 1	PRO: 180 ± 2<br>YTH: 174 ± 10	PRO: 75 ± 5<br>YTH: 65 ± 1	NC	SG	Baseline RS test before an intervention.
Rodríguez-Fernández et al. [284]	24	SOC	TRA	19 ± 2	176 ± 6	67 ± 9	NC	SG	Baseline RS test before an intervention
Røksund et al. [285]	75	SOC	NAT	19 ± 3	181 ± 6	75 ± 10	NC	OBS	Single RS test as part of a testing battery.
Ruscello et al. [286]	15 (0%)	SOC	NAT	23 ± 6	165 ± 6	59 ± 9	NC	CRO (r)	2 different RS tests on AG, separated by > 48-hrs.
Ruscello et al. [104]	17	SOC	NAT	22 ± 4	177 ± 6	72 ± 10	NC	CRO (r)	2 different RS tests on AG, separated by > 48-hrs.
Russell et al. [123]	14	SOC	NAT	18 ± 2	178 ± 5	75 ± 6	NC	CRO (ran)	Baseline RS test before an intervention.
Salleh et al. [287]	24	SOC	TRA	21 ± 2	173 ± 3	65 ± 3	NC	OBS	Single RS test.
Sánchez-Sánchez et al. [117]	18	SOC	TRA	22 ± 2	175 ± 6	74 ± 9	NC	OBS	RS test on 4 different AG pitches, separated by 72 hrs.
Sánchez-Sánchez et al. [288]	21	SOC	NAT	U18	NR	NR	NC	OBS	Single RS test.
Sánchez-Sánchez et al. [289]	16	SOC	NAT / TRA	21 ± 1	69 ± 5	177 ± 5	C	PAG	Baseline RS test before an intervention
Sanders et al. [290]	20 (50%)	SOC	INTL	M: 21 ± 1<br>F: 20 ± 1	M: 178 ± 7<br>F: 168 ± 6	M: 75 ± 5<br>F: 63 ± 5	NC	OBS	Single RS test.
Scanlan et al. [291]	9 (67%)	MIX	TRA	22 ± 4	171 ± 6	73 ± 12	NC	CRO (ran)	Two different RS protocols on an indoor, sprung, hardwood surface.
Scanlan et al. [292]	8 (75%)	BB	TRA	20 ± 1	183 ± 10	78 ± 17	NC	CRO	RS protocol an indoor, hardwood BB court.
Selmi et al. [58]	24	SOC	NAT	17 ± 0	172 ± 9	68 ± 7	NC	CRO (ran)	3 different RS tests on outdoor AG, separated by > 48-hrs.
Selmi et al. [293]	30	SOC	NAT	18 ± 1	178 ± 5	70 ± 7	C	PAG (r)	Baseline RS test before an intervention

Study	Participants						Experimental Approach		
	N#	Sport	Level	Age (yrs)	Stature (cm)	Body mass (kg)	Design	Type	Details
Shalfawi et al. [294]	30 (0%)	SOC	NAT	19 ± 4	167 ± 4	58 ± 7	NC	OBS	RS test in an indoor arena.
Shalfawi et al. [295]	15	SOC	NAT	16 ± 1	179 ± 7	68 ± 9	C	PAG (r)	RS test on indoor AG before an intervention
Shalfawi et al. [296]	17 (0%)	SOC	TRA	21 ± 3	1769 ± 5	64 ± 6	C	PAG (r)	RS test on an indoor Mondo track
Silva et al. [297]	22	SOC	NAT	18 ± 1	175 ± 6	71 ± 5	NC	SG	Baseline RS test before an intervention.
Soares-Caldeira et al. [298]	14	FUT	NAT	INT: 25 ± 8<br>CON: 21 ± 5	172 ± 6	72 ± 9	C	PAG (r)	RS test on an indoor synthetic floor, before an intervention.
Spineti et al. [299]	22	SOC	NAT	18 ± 0	180 ± 8	70 ± 9	NC	PAG (r)	RS test before an intervention
Stojanovic et al. [300]	24	BB	NAT	22. ± 3	197 ± 6	96 ± 9	NC	OBS	RS test on a BB court, as part of a testing battery.
Suarez-Arrones et al. [105]	16	RUG	TRA	27 ± 5	180 ± 7	91 ± 16	C	PAG (r)	Data extracted from baseline RS tests (both groups) and training data (RST group).
Taylor et al. [2]	15	SOC	TRA	24 ± 4	179 ± 6	77 ± 8	NC	PAG	Data extracted from a RST intervention.
Teixeira et al. [301]	20 (0%)	FUT	NAT	19 ± 2	162 ± 5	59 ± 8	NC	PAG (r)	Baseline RS test on an indoor FUT court, as part of a testing battery, before a long-term training intervention.
Thomassen et al. [302]	18	SOC	NAT	23 ± 1	182 ± 2	79 ± 2	NC	PAG (r)	Baseline RS test on an indoor wooden surface.
Tønnessen et al. [303]	20	SOC	NAT	16 ± 1	176 ± 7	67 ± 9	C	PAG (r)	Baseline RS test before an intervention.
Torreblanca-Martinez et al.[304]	18 (0%)	SOC	NAT	18 ± 2	162 ± 5	56 ± 7	NC	SG	RS test on outside AG.
Tounsi et al. [176]	33	SOC	NAT	17 ± 0	NR	NR	NC	CRO (ran)	RS test on NG
Trecroci et al. [305]	9	SOC	NAT	17–19	177 ± 2	66 ± 6	NC	CRO (r)	Baseline RS test on NG, before an intervention.
Turki et al. [111]	19	SOC	NR	18 ± 1	175 ± 7	70 ± 8	C	CRO (ran) (r)	Baseline RS test.

Study	Participants						Experimental Approach		
	N#	Sport	Level	Age (yrs)	Stature (cm)	Body mass (kg)	Design	Type	Details
Ulupinar et al. [126]	18	SOC	TRA	20 ± 2	178 ± 5	72 ± 6	NC	CRO (ran)	2 different RS protocols on outdoor NG, separated by > 48 hrs
Ulupinar et al. [125]	16	SOC	TRA	19 ± 2	176 ± 5	70 ± 6	NC	CRO (ran)	4 different RS protocols on indoor AG, separated by > 48 hrs
Van den Tillaar et al. [306]	17 (0%)	SOC	NR	17 ± 1	168 ± 5	62 ± 7	NC	OBS	Single RS test on a track.
Vasquez-Bonilla et al. [307]	38 (0%)	SOC	NAT	23 ± 4	165 ± 11	61 ± 7	NC	OBS	Single RST test on an indoor court
Wadley & Le Rossignol [308]	17	ARF	NAT	21 ± 2	182 ± 5	81 ± 10	NC	OBS	RS test on an asphalt surface, as part of a testing battery.
West et al. [309]	15	RUG	NAT	28 ± 3	188 ± 6	99 ± 9	C	CRO (ran)	RS test on an indoor sprint track.
Woolley et al. [33]	10	MIX	NR	27 ± 3	178 ± 6	78 ± 8	NC	CRO (ran)	RS protocol on a non-slip indoor surface
Yanci et al. [310]	39	FUT	TRA	23 ± 5	170 ± 10	69 ± 10	C	PAG (r)	Baseline RS test before an intervention
Zagatto et al. [106]	20	BB	NAT	17 ± 1	191 ± 8	84 ± 12	NC	CRO (ran)	2 different RS tests on an indoor court, separated by 2–4 days.
Zagatto et al. [311]	12	BB	NAT	25 ± 7	200 ± 10	97 ± 9	C	CRO (r)	RS test on a BB court, CON condition only.
Zagatto et al. [107]	10	BB	NAT	17 ± 1	191 ± 7	87 ± 15	C	CRO (ran)	Single RS protocol on a BB court

Data are presented as mean ± standard deviation.

Abbreviations: N# = number of participants (unless stated, the proportion of males was 100%). M = male; F = female; NR = not reported; NA = not applicable; OBS = observational design; CRO = crossover design; SG = single group pre-test post-test design; ran = experimental treatment or measurements delivered in a randomised order; r = random assignment of participants to experimental groups; C = controlled study; NC = non-controlled study; PLA = placebo; SOC = soccer, FUT = futsal; RUG = rugby; HOC = field hockey; BB = basketball; AF = American football; ARF = Australian rules football; VB = volleyball; HB = handball; NET = netball; MIX = mixture of team sports; TRA = trained/developmental athletes; INT = international/elite athletes; NAT = national/highly trained athletes; PRO = professional; SEMI = semi-professional; AM = amateur; YTH = youth; CON = control group; INT = intervention group; Sham = sham group; RS = repeated-sprint; RS-G = repeated-sprint group; PLY = plyometric group; REP = representative players; Club = club players; MID = midfielders; FWD = forwards; DEF = defenders; G = guards; CEN = centres; U17 = under 17 players; U18 = under 18 players; U20 = under 20 players; SEN = senior players; VO_{2max} = maximal oxygen consumption; High = high $V0_2max$ group; Med = medium VO_{2max} group; Low = low VO_{2max} group; SAN = sand training group; GRA = grass training group; TS3 = team sport 3; ST = starting players; N-ST = non-starting players; N-SEL = non-selected players; MG = Melanesian group; N-MG = Non-Melanesian group; S_{dec} = percentage sprint decrement; yrs = years; hrs = hours; AG = artificial grass; NG = natural grass; cm = centimetre; kg = kilogram; ~ = approximately; * = single group time series.

Supplementary Table S3. Summary of exercise protocol information and outcomes from all studies.

Study	Exercise protocol						Outcomes			
	RST Mode	Sets × Reps	Distance / Duration	Rest Time	Rest Mode	I-set Rest	Performance	Perceptual	Neuromuscular	Physiological
Abt et al. [118]	STR	1 × 22	15 m	1:10^{N} (~26 s)	A^{H}	-	S_{avg}: 2.64 ± 0.06 s	-	-	$B[La]_{peak}$: 1.3 ± 0.2 to 7.6 ± 0.6 mmol·L^{-1}
	STR	1 × 22	15 m	1:10^{N} (~26 s)	P	-	S_{avg}: 2.63 ± 0.07 s	-	-	$B[La]_{peak}$: 1.0 ± 0.1 to 8.7 ± 0.9 mmol·L^{-1}
	STR	1 × 22	30 m	1:10^{N} (~45 s)	A^{H}	-	S_{avg}: 4.57 ± 0.22 s	-	-	$B[La]_{peak}$: 1.2 ± 0.2 to 10.6 ± 0.7 mmol·L^{-1}
	STR	1 × 22	30 m	1:10^{N} (~45 s)	P	-	S_{avg}: 4.59 ± 0.15	-	-	$B[La]_{peak}$: 1.3 ± 0.2 to 11.1 ± 0.8 mmol·L^{-1}
AbuMoh'd [180]	STR	1 × 7	30 m	30 s	P	-	INT, S_{avg}: 3.71 ± 0.05; PLA, S_{avg}: 3.70 ± 0.05	-	-	INT, $B[La]^{5'}$: 9.0 ± 0.1 mmol·L^{-1}; PLA, $B[La]^{5'}$: 9.2 ± 0.2 mmol·L^{-1}
Akenhead et al. [57]	SHU	1 × 12	25 m (12.5 + 12.5)	20 s	P	-	S_{dec}: 5.3%	-	-	-
Aguiar et al. [95]	MD^{A}	1 × 7	34.2 m	25 s	A^{K}	-	INT, S_{avg}: 6.69 ± 0.20 s CON, S_{avg}: 7.31 ± 0.34 s	-	-	-
Alemdaroğlu et al. [23]	SHU	1 × 6	40 m	On 25 s (~17 s)	A^{H}	-	S_{best}: 7.35 ± 0.17 s; S_{total}: 45.93 ± 0.84 s; S_{dec}: 4.13 ± 1.81%	-	-	$B[La]^{3'}$: 9.3 ± 2.5 mmol·L^{-1}
	STR	1 × 6	40 m	On 25 s (~19 s)	A^{H}	-	S_{best}: 5.68 ± 0.20 s; S_{total}: 34.90 ± 1.21 s; S_{dec}: 2.42 ± 1.43%	-	-	$B[La]^{3'}$: 7.6 ± 1.4 mmol·L^{-1}
	SHU	1 × 8	30 m (15 + 15)	On 25 s (~19 s)	A^{H}	-	S_{best}: 5.64 ± 0.16 s; S_{total}: 46.41 ± 1.32 s; S_{dec}: 2.85 ± 1.51%	-	-	$B[La]^{3'}$: 7.9 ± 2.1 mmol·L^{-1}
	STR	1 × 8	30 m	On 25 s (~20 s)	A^{H}	-	S_{best}: 4.50 ± 0.15 s; S_{total}: 37.21 ± 1.23 s; S_{dec}: 3.29 ± 0.91%	-	-	$B[La]^{3'}$: 8.1 ± 1.4 mmol·L^{-1}
Alizadeh et al. [167]	STR	1 × 6	35 m	10 s	P	-	High, S_{best}: 5.34 ± 0.13 s; S_{total}: 33.47 ± 0.99 s; S_{dec}: 9.6 ± 0.1%; Med, S_{best}: 5.39 ± 0.14 s; S_{total}: 34.77 ± 0.56 s; S_{dec}: 9.3 ± 0.2%; Low, S_{best}: 6.22 ± 0.39 s; S_{total}: 40.56 ± 3.50 s; S_{dec}: 9.2 ± 0.3%	-	-	High, △ $B[La]^{3'}$: 1.73 to 6.97 mmol·L^{-1}; MED, △ $B[La]^{3'}$: 1.9 to 9.0 mmol·L^{-1}
Almansba et al. [96]	MD^{Y}	1 × 6	40	20 s	P	-	S_{best}: 7.97 ± 0.39 s; S_{avg}: 8.37 ± 0.30 s; S_{dec}: 4.8 ± 2.0%	6–20: 15.2 ± 1.6 au	-	$B[La]^{2'}$: 12.9 ± 1.5 mmol·L^{-1}; HR_{peak}: 189 ± 7 b·min^{-1} HRav: 195 ± 8 b·min^{-1}

Study	Exercise protocol						Outcomes			
	**RST Mode**	**Sets × Reps**	**Distance / Duration**	**Rest Time**	**Rest Mode**	**I-set Rest**	**Performance**	**Perceptual**	**Neuromuscular**	**Physiological**
	STR	1 × 6	40	20 s	P	-	S_{best}: 5.75 ± 0.28 s; S_{avg}: 6.16 ± 0.29 s; S_{dec}: 6.7 ± 3.1%	6–20: 13.9 ± 1.8 au		$B[La]^{2'}$: 11.6 ± 1.2 mmol·L^{-1}; HR_{peak}: 185 ± 6 b·min^{-1}; HR_{avg}: 178 ± 9 b·min^{-1}
Altimari et al. [181]	SHU	1 × 6	40 m (20 + 20)	20 s	P	-	1TR, S_{avg}: 7.08 ± 0.27 s; S_{dec}: 5.3 ± 1.3%; 2TR, S_{avg}: 7.16 ± 0.25 s; S_{dec}: 5.4 ± 1.2%; 3TR, S_{avg}: 7.08 ± 0.27 s; S_{dec}: 5.6 ± 1.5%	-	-	-
Archiza et al. [182]	SHU	1 × 6	40 m (20 + 20)	20 s	P	-	Sham, S_{best}: 7.50 ± 0.20 s; S_{avg}: 7.90 ± 0.20 s; S_{dec}: 6.3 ± 3.0%; INT, S_{best}: 7.60 ± 0.30 s; S_{avg}: 8.20 ± 0.30 s; S_{dec}: 7.9 ± 2.4%	-	-	-
Attene et al. [115]	SHU	1 × 10	30 m (15 + 15)	30 s	P	-	S_{best}: 6.41 ± 0.43 s; S_{total}: 67.27 ± 4.43 s; S_{dec}: 10.9 ± 4.3%	CR10: 8.6 ± 0.5 au	-	$B[La]^{3'}$: 9.5 ± 1.6 mmol·L^{-1}
Ayarra et al. [183]	STR	1 × 6	30 m	25 s	A	-	S_{total}: 26.03 ± 2.09 s; S_{dec}: 1.7 ± 3%	-	-	-
Aziz et al. [184]	STR	1 × 8	40 m	30 s	A[1]	-	S_{best}: 5.45 ± 0.23 s; S_{total}: 45.90 ± 1.64 s; S_{dec}: 5.4 ± 2.7%		-	-
Baldi et al. [185]	SHU	1 × 6	40 m (20 + 20)	20 s	P	-	S_{best}: 7.13 ± 0.24 s; S_{dec}: 5.2 ± 1.6%		-	$B[La]_{peak}$:17.6 ± 2.6 mmol·L^{-1}
Balsalobre-Fernández et al. [186]	STR	1 × 6	35 m	10 s	P	-	-	-	△ CMJ[AA]: -4.2 cm (-9.2 ± 4.8%)	-
Beato et al. [187]	SHU	1 × 6	40 m (20 + 20)	20 s	P	-	STR-G, S_{best}: 7.13 ± 0.17 s, S_{avg}:7.46 ± 0.19 s; SHU-G group, S_{best}: 7.14 ± 0.18 s, S_{avg}:7.50 ± 0.21 s	-	-	-
	STR	3 × 7	30 m	20 s	P	4 min P	-	CR10: 6.3 ± 0.5 au	-	-
	SHU	3 x 7	40 m	20 s	P	4 min P	-	CR10: 6.4 ± 0.6 au	-	-
Beato et al. [188]	SHU	1 × 6	40 m (20 + 20)	20 s	P	-	STR-G, S_{best}: 7.30 ± 0.15 s; S_{avg}: 7.56 ± 0.20 s SHU-G, S_{best}: ± 7.23 ± 0.32 s; S_{avg}: 7.46 ± 0.31 s	-	-	-
	STR	3 × 7	30 m	20 s	P	4 min P	-	CR10: 6.1 ± 0.8 au	-	-

Study	Exercise protocol						Outcomes			
	RST Mode	Sets × Reps	Distance / Duration	Rest Time	Rest Mode	I-set Rest	Performance	Perceptual	Neuromuscular	Physiological
	SHU	3 × 7	40 m (20 + 20)	20 s	P	4 min p	-	CR10: 6.4 ± 0.7	-	-
Beato & Drust [162]	STR	3 × 7	30 m	25 s	A[Q]	3 min P	-	-	-	HR_{peak}: 192 ± 12 b·min^{-1}
Beaven et al. [189]	STR	1 × 5	40 m	On 30 s (~24 s)	P	-	S_{total}: 27.58 ± 1.58 s		-	HR_{post}: 139 ± 8 b·min^{-1}
Binnie et al. [190]	STR	1 × 8	20 m	20 s	A[W]	-	SAN, S_{total}: 30.97 ± 1.58 s; S_{dec}: 4.8 ± 2.1%; GRA, S_{total}: 29.56 ± 1.69 s; S_{dec}: 4.5 ± 2.2%	-	-	SAN, $B[La]_{peak}$: 6.5 ± 2.3 mmol·L^{-1}; GRA, $B[La]_{peak}$: 5.7 ± 2.5 mmol·L^{-1}
Binnie et al. [191]	STR	1 × 8	20 m	20 s	A[K]	-	S_{best}: 3.31 s; S_{total}: 27.46 s; S_{dec}: 3.7%		-	$B[La]_{post}$: 8.2 mmol·L^{-1} HR_{peak}: 160 b·min^{-1}
Binnie et al. [192]	STR	1 × 8	20 m	20 s	A[K]	-	S_{best}: 3.34 s; S_{total}: 27.94 s; S_{dec}: 4.4%	-	-	$B[La]_{post}$: 7.5 mmol·L^{-1} HR_{peak}: 163 b·min^{-1}
Blasco-Lafarga et al. [108]	MD[C]	1 × 7	34.2 m	25 s	A[K]	-	S_{best}: 5.72 ± 0.13 s; S_{avg}: 5.91 ± 0.14 s; S_{total}: 41.41 ± 0.99 s; S_{dec}: 3.5 ± 1.6%	CR10: 9.1 ± 2.2 au	-	$B[La]^{3'}$: 8.5 ± 1.4 mmol·L^{-1}
Borges et al. [193]	SHU	1 × 6	40 m (20 + 20)	20 s	P	-	RES, S_{best}: 7.35 ± 0.07 s; S_{avg}: 7.70 ± 0.14 s; PLY, S_{best}: 7.21 ± 0.18 s; S_{avg}: 7.55 ± 0.22 s	-	-	-
Brahim et al. [97]	SHU	1 × 6	40 m (20 + 20)	20 s	P	-	S_{dec}: 2.7 ± 1.3%		-	-
	MD[B]	1 × 12	20 m	40 s	P	-	S_{dec}: 3.8 ± 2.3%		-	-
	MD[A]	1 × 7	34.2 m	25 s	A[K]	-	S_{dec}: 4.3 ± 3.4%		-	-
Brini et al. [154]	SHU	1 × 10	30 m	30 s	P	-	S_{best}: 5.80 ± 0.21 s; S_{total}: 58.99 ± 1.67 s	CR10: 4.3 ± 0.5 au	-	$B[La]_{post}$: 5.3 ± 1.7 mmol·L^{-1}; HR_{peak}: 194 ± 2 b·min^{-1}
	SHU	1 × 10	30 m	30 s	A[X]	-	S_{best}: 5.88 ± 0.15 s; S_{total}: 59.58 ± 1.36 s	CR10: 5.0 ± 0.6 au		$B[La]_{post}$: 5.5 ± 2.1 mmol·L^{-1}; HR_{peak}: 195 ± 2 b·min^{-1}
	SHU	1 × 10	30 m	30 s	A[Y]	-	S_{best}: 5.91 ± 0.15 s; S_{total}: 60.02 ± 1.11 s	CR10: 7.4 ± 0.8 au	-	$B[La]_{post}$: 6.8 ± 2.2 mmol·L^{-1}; HR_{peak}: 195 ± 2 b·min^{-1}
	SHU	1 × 10	30 m	30 s	A[Z]	-	S_{best}: 5.92 ± 0.11 s; S_{total}: 60.10 ± 0.94 s	CR10: 8.2 ± 0.8 au	-	$B[La]_{post}$: 6.6 ± 2.1 mmol·L^{-1}; HR_{peak}: 196 ± 2 b·min^{-1}

Study	Exercise protocol						Outcomes			
	RST Mode	Sets × Reps	Distance / Duration	Rest Time	Rest Mode	I-set Rest	Performance	Perceptual	Neuromuscular	Physiological
Brini et al. [194]	SHU	1 × 10	30 m (15 + 15)	30 s	P	-	SSG, S_{best}: 5.90 ± 0.11 s; S_{avg}: 5.98 ± 0.68 s; S_{total}: 59.78 ± 0.68 s; RS, S_{best}: 5.88 ± 0.13 s; S_{avg}: 5.97 ± 1.14 s; S_{total}: 59.72 ± 1.14 s	-	-	SSG, HR_{peak}: 186 ± 4 b·min^{-1}; RS, HR_{peak}: 189 ± 3 b·min^{-1}
Brini et al. [195]	SHU	1 × 10	30 m (15 + 15)	30 s	P	-	S_{best}: 5.89 ± 0.10 s; S_{total}: 59.60 ± 0.90 s; S_{dec}: 1.2 ± 0.5%	CR10: 7 ± 1 au	-	B[La]$^{3'}$: 6.6 ± 2.1 mmol·L^{-1}; HR_{peak}: 191 ± 1 b·min^{-1}
	MD	1 × 10	30 m	30 s	P	-	S_{best}: 5.90 ± 0.10 s; S_{total}: 59.80 ± 0.90 s; S_{dec}: 1.3 ± 0.5%	CR10: 8 ± 1 au	-	B[La]$^{3'}$: 6.8 ± 2.2 mmol·L^{-1}; HR_{peak}: 195 ± 1
Brini et al. [98]	MD	1 × 10	30 m	30 s	P	-	INT, S_{best}: 6.91 ± 0.1s; S_{total}: 70.90 ± 0.98 s; CON, S_{best}: 6.87 ± 0.12 s; CON, S_{total}: 69.81 ± 0.62 s	INT, CR10: 5.6 ± 1.4 au; CON, CR10: 6.0 ± 1.3 au	-	INT, B[La]$^{3'}$: 5.4 ± 2.1 mmol·L^{-1}; HR_{peak}: 187 ± 3 b·min^{-1}; CON, B[La]$^{3'}$: 5.8 ± 2.4 mmol·L^{-1}; HR_{peak}: 187 ± 6 b·min^{-1}
Brini et al. [46]	MD^{A}	1 × 10	30 m	30 s	P	-	PRO, S_{best}: 8.07 ± 0.03 s; S_{total}: 83.35 ± 2.19 s; SEMI, S_{best}: 8.21 ± 0.16 s; S_{total}: 83.56 ± 2.17 s;	PRO, CR10: 6.8 ± 0.6 SEMI, CR10: 6.9 ± 0.6	-	PRO, B[La]$^{3'}$: 8.0 ± 2.0 mmol·L^{-1}; HR_{peak}: 187 ± 2 b·min^{-1} SEMI, B[La]$^{3'}$: 9.5 ± 0.6 mmol·L^{-1}; HR_{peak}: 189 ± 1 b·min^{-1}
Brocherie et al. [196]	STR	1 × 6	35 m	10 s	P	-	S_{best}: 4.87 ± 0.14 s; S_{total} 31.73 ± 1.13 s; S_{dec}: 8.7 ± 2.3%		-	-
Brocherie et al. [54]	STR	1 × 6	35 m	10 s	P	-	S_{avg}: 5.34 ± 0.25 s; S_{dec}: 9.5 ± 2.4%	6–20: 15.9 ± 0.9 au	△ sprint 1–6: ΔL: 17.5 ± 2.5 to 18.0 ± 2.9 cm; Δz: 1.9 ± 0.3 to 2.7 ± 0.3 cm; $F_z$max: 2.36 ± 0.18 to 2.41 ± 0.14 N; K_{vert}: 127.6 ± 17.7 to 91.4 ± 10.4 kN·m^{-1}; K_{leg}: 13.7 ± 1.7 to 13.8 ± 2.7 kN·m^{-1}	B[La]$^{4'}$: 10.5 ± 2.0 mmol·L^{-1}
Brocherie et al. [197]	STR	1 × 8	20 m	On 20 s (~17 s)	P	-	HYP, S_{total}: 27.23 ± 1.15 s; S_{dec}: 4.0 ± 1.7%; NOR, 27.05 ± 0.81 s; S_{dec}: 4.3 ± 1.9%; CON, 26.98 ± 1.03 s; S_{dec}: 5.2 ± 2.1%	-	-	-

Study	Exercise protocol						Outcomes			
	**RST Mode**	**Sets × Reps**	**Distance / Duration**	**Rest Time**	**Rest Mode**	**I-set Rest**	**Performance**	**Perceptual**	**Neuromuscular**	**Physiological**
Broderick et al. [141]	STR	1 × 3	15 m	20 s	P	-	INT, S_{best}: 2.58 ± 0.10 s; S_{total}: 7.82 ± 0.32 s; CON, S_{best}: 2.58 ± 0.10 s; S_{total}: 7.84 ± 0.31 s	-	-	-
Buchheit [198]	STR	1 × 6	30 m	20 s	P	-	S_{best}: 5.73 ± 0.27 s; S_{avg}: 5.90 ± 0.27 s; S_{dec}: 2.8 ± 0.9%	-	-	-
	SHU	1 × 6	25 m	25 s	A^L	-	S_{best}: 3.96 ± 0.15 s; S_{avg}: 4.09 ± 0.17 s; S_{dec}: 3.2 ± 1.3%			
Buchheit et al. [59]	STR	1 × 6	25 m	On 25s (~21 s)	A^L	-	S_{best}: 3.97 ± 0.15 s; S_{avg}: 4.09 ± 0.16 s S_{dec}: 2.8 ± 1.2%	CR10: 7 ± 1 au	-	$B[La]^{3'}$: 9.4 ± 2.4 mmol·L^{-1}; VO_{2avg}: 38.1 ± 5.0 ml·min^{-1}·kg^{-1} (% VO_{2max}: 76 ± 10%); HR_{peak}: 175 ± 11 b·min^{-1} (% HR_{max}: 95 ± 6%)
	SHU	1 × 6	25 m (12.5 + 12.5)	On 25 s (~20 s)	A^L	-	S_{best}: 5.186 ± 0.16 s; S_{avg}: 5.29 ± 0.17 s; S_{dec}: 2.5 ± 1.0%	CR10: 7 ± 1 au	-	$B[La]^{3'}$: 9.9 ± 2.0 mmol L^{-1}; VO_{2avg}: 39.7 ± 5.0 ml·min^{-1}·kg^{-1} (% VO_{2max}: 79 ± 10%); HR_{peak}: 177 ± 11.0 b·min^{-1} (% HRmax: 96 ± 6%)
	STR	1 × 6	25 m	On 25 s (~21 s)	A^M	-	S_{best}: 3.98 ± 0.14 s; S_{avg}: 4.14 ± 0.17 s S_{dec}: 3.9 ± 1.5%	CR10: 8 ± 1 au	-	$B[La]^{3'}$: 10.2 ± 2.4 mmol·L^{-1}; VO_{2avg}: 40.2 ± 4.5 ml·min^{-1}·kg^{-1} (% VO_{2max}: 80 ± 9%); HR_{peak}: 176 ± 11 b·min^{-1} (% HRmax: 96 ± 6%)
	SHU	1 × 6	25 m (12.5 + 12.5)	On 25 s (~20 s)	A^M	-	S_{best}: 5.18 ± 0.18 s; S_{avg}: 5.43 ± 0.18 s S_{dec}: 3.4 ± 2.3%	CR10: 8 ± 1 au	-	$B[La]^{3'}$: 10.4 ± 2.1 mmol·L^{-1}; VO_{2avg}: 42.2 ± 5.0 ml·min^{-1}·kg^{-1} (% VO_{2max}: 84 ± 10%); HR_{peak}: 178 ± 11 b·min^{-1} (% HRmax: 97 ± 6%)
Buchheit et al. [60]	STR	1 × 6	25 m	On 25 s (~21 s)	A^L	-	S_{best}: 3.96 ± 0.15 s; S_{avg}: 4.09 ± 0.17 s S_{dec}: 3.2 ± 1.3%	CR10: 7.2 ± 1.4 au	-	△ $B[La]^{3'}$: 2.2 ± 0.2 to 9.3 ± 2.4 mmol·L^{-1}; VO_{2avg}: 35.8 ± 4.7 ml·min^{-1}·kg^{-1} (% VO_{2max}: 77.4 ± 9.3%); HR_{peak}: 173 ± 9 b·min^{-1} (% HRmax: 94 ± 5%)

Study	Exercise protocol						Outcomes			
	RST Mode	Sets × Reps	Distance / Duration	Rest Time	Rest Mode	I-set Rest	Performance	Perceptual	Neuromuscular	Physiological
	SHU	1 × 6	25 m (12.5 + 12.5)	On 25 s (~20 s)	A^L	-	S_{best}: 5.16 ± 0.17 s; S_{avg}: 5.30 ± 0.17 s S_{dec}: 2.6 ± 1.2%	CR10: 7.2 ± 0.8 au	-	△ $B[La]^{3'}$: 2.2 ± 0.2 to 10.0 ± 1.7 mmol·L^{-1}; VO_{2avg}: 40.4 ± 5.2 ml·min^{-1}·kg^{-1} (% VO_{2max}: 80.5 ± 10.3%); HR_{peak}: 173 ± 10 b·min^{-1} (% HRmax: 94 ± 5%)
Buchheit et al. [99]	STR	1 × 6	30 m	On 25 s (~20 s)	A^L	-	S_{best}: 4.37 ± 0.17 s; S_{avg}: 4.69 ± 0.20 s S_{dec}: 6.7 ± 2.5%	CR10: 7.4 ± 1.5 au	-	△ $B[La]^{3'}$: ↑ 10.1 ± 2.2 mmol·L^{-1}; HR_{peak}: 184 ± 7 b·min^{-1}
	MD^D	1 × 6	~27.6 m	On 25 s (~20 s)	A^L	-	S_{best}: 4.38 ± 0.17 s; S_{avg}: 4.61 ± 0.29 s S_{dec}: 4.8 ± 3.6%	CR10: 6.9 ± 1.7 au	-	△ $B[La]^{3'}$: ↑ 8 ± 2.3 mmol·L^{-1}; HR_{peak}: 181 ± 8 b·min^{-1}
	MD^E	1 × 6	~21.2 m	On 25 s (~20 s)	A^L	-	S_{best}: 4.36 ± 0.15 s; S_{avg}: 4.69 ± 0.16 s S_{dec}: 7.0 ± 3.2%	CR10: 6.0 ± 1.6 au	-	△ $B[La]^{3'}$: ↑ 6.1 ± 2.5 mmol·L^{-1}; HR_{peak}: 178 ± 9 b·min^{-1}
	MD^F	1 × 6	~ 19.2 m	On 25 s (~20 s)	A^L	-	S_{best}: 4.39 ± 0.19 s; S_{avg}: 4.73 ± 0.19 s S_{dec}: 7.1 ± 3.0%	CR10: 6.0 ± 1.1 au	-	△ $B[La]^{3'}$: ↑ 7.4 ± 2.3 mmol·L^{-1}; HR_{peak}: 180 ± 8 b·min^{-1}
Campa et al. [168]	SHU	1 × 6	40 m (20 + 20)	20 s	P	-	EL, S_{best}: 7.00 ± 0.30 s; S_{avg}: 7.50 ± 0.40 s; S_{dec}: 6.3 ± 3.1%; S-EL, S_{best}: 7.70 ± 0.20 s; S_{avg}: 7.90 ± 0.20 s; S_{dec}: 3.4 ± 1.1%	-	-	-
Campos et al. [199]	SHU	1 × 8	40m (10 + 20 + 10)	20 s	P	-	IT_{100}, S_{best}: 8.12 ± 0.20 s; S_{avg}: 8.69 ± 0.36 s; IT_{86}, S_{best}: 8.28 ± 0.24 s; S_{avg}: 8.50 ± 0.18 s	-	-	-
Campos-Vazquez et al. [200]	SHU	1 × 6	40 m (20 + 20)	20 s	P	-	SQ, S_{best}: 6.99 ± 0.11 s; S_{avg}: 7.40 ± 0.18 s; TG, S_{best}: 7.07 ± 0.18 s; S_{avg}: 7.42 ± 0.15 s	-	-	-
Caprino et al. [201]	SHU	1 × 10	30 m (15 + 15)	30 s	P	-	S_{total}: 58.80 ±2.10 s; S_{dec}: 2.3 ± 1.0%	-	-	△ $B[La]^{3'}$: 5.1 ± 1.4 mmol·L^{-1} to 12.4 ± 2.8 mmol·L^{-1}
Castagna et al. [153]	SHU	1 × 10	30 m (15 + 15)	30 s	P	-	S_{avg}: 6.17 ± 0.10 s; S_{total}: 60.56 ± 1.60 s; S_{dec}: 3.4 ± 2.3%	-	-	△ $B[La]^{3'}$: 2.5 ± 0.7 mmol L^{-1} to 14.1 ± 3.5 mmol·L^{-1}
	SHU	1 × 10	30 m (15 + 15)	30 s	A^Z	-	S_{avg}: 6.32 ± 0.10 s; S_{total}: 62.15 ± 2.99 s S_{dec}: 5.0 ± 2.4%	-	-	△ $B[La]^{3'}$: 2.4 ± 0.5 to 13.2 ± 2.9 mmol·L^{-1}

Study	Exercise protocol						Outcomes			
	RST Mode	Sets × Reps	Distance / Duration	Rest Time	Rest Mode	I-set Rest	Performance	Perceptual	Neuromuscular	Physiological
Castagna et al. [202]	SHU	1 × 10	30 m (15 + 15)	30 s	P	-	S_{dec}: 3.4 ± 2.3%	-	-	△ $B[La]_{post}$: 2.5 ± 0.7 to 13.6 ± 3.1 mmol·L^{-1}; △ $B[La]^{3'}$: 2.5 ± 0.7 to 14.2 ± 3.5 mmol·L^{-1}
Chaouachi et al. [203]	STR	1 × 7	30 m	25 s	A^{Q}	-	S_{avg}: 4.50 ± 0.13 s; S_{total}: 31.21 ± 1.13 s; S_{dec}: 6.0 ± 2.5%	-	-	-
Charlot et al. [204]	STR	1 × 6	25 m	25 s	A^{K}	-	S_{avg}: 3.84 ± 0.17 s: S_{total}: 23.10 ± 1.10 s; S_{dec}: 7.4 ± 3.9%	-	-	-
	SHU	1 × 6	25 m (12.5 + 12.5)	25 s	A^{K}	-	S_{avg}: 5.32 ± 0.17 s: S_{total}: 30.50 ± 2.30 s; S_{dec}: 4.1 ± 1.3%	-	-	-
Chen et al. [205]	SHU	1 × 6	40 m (20 + 20)	20 s	P	-	S_{best}: 7.50 ± 0.50 s; S_{total}: 45.9 ± 3.34 s; S_{dec}: 3.5 ± 2.5%	6–20: 15 ± 3.6 au	-	$B[La]_{post}$: 9.8 ± 2.1 mmol·L^{-1}; HR_{peak}:171 ± 12 b·min^{-1}
Clifford et al. [34]	STR	1 × 20	30 m	30 s	P	-	INT, S_{best}: 4.41 ± 0.23 s; S_{avg}: 4.65 ± 0.25 s; PLA: 4.48 ± 0.14 s; S_{avg}: 4.70 ± 0.15 s	INT, 6–20: 15 ± 1 au; PLA, 6–20: 14 ± 2 au	INT, △ CMJ^{AA}: -11.8 ± 8.9%; PLA, △ CMJ^{AA}: -9.6 ± 4.8%	INT, CK 24 h: 188 ± 62 to 542 ± 461 u·L^{-1} (188%); PLA, CK 24 h: 318 ± 145 to 592 ± 321 u·L^{-1} (86%)
Corrêa et al. [206]	STR	1 × 6	35 m	10 s	P	-	S_{total}: 31.17 ± 1.03 s; S_{dec}: 8.2 ± 2.77%	-	-	-
Costello et al. [127]	STR	1 × 20	20 m	20 s	A	-	S_{avg}: 3.43 ± 0.2 s	CR10: 9 ± 1.1	-	$B[La]_{post}$: 12.4 ± 2.6 mmol·L^{-1}: HR_{avg}: 178 ± 8 b·min^{-1}
Cuadrado-Peñafiel et al. [207]	SHU	1 × 6	40 m (20 + 20)	30 s	P	-	SOC, S_{best}: 7.01 ± 0.22 s; S_{dec}: 2.7 ± 0.6%; FUT: 7.26 ± 0.19 s; S_{dec}: 4.4 ± 1.2%	-	-	SOC, $B[La]_{post}$: 13.7 ± 2.8 mmol·L^{-1}; FUT, $B[La]_{post}$: 14.3 ± 3.4 mmol·L^{-1}
Da Silva et al. [208]	SHU	1 × 7	34.2 m	25 s	P	-	S_{best}: 6.30 ± 0.24 s; S_{avg}: 6.56 ± 0.23 s; S_{dec}: 4.0 ± 1.9%	-	-	$B[La]_{peak}$: 15.4 ± 2.2 mmol·L^{-1}
Dal Pupo [116]	STR	1 × 6	25 m	15 s	A	-	S_{best}: 3.80 ± 0.18 s; S_{avg}: 3.98 ± 0.20 s; S_{dec}: 4.7 ± 2.0%	-	△CMJ^{AB}: 43.52 ± 1.48 to 41.68 ± 1.25 cm (-4.2%)	$B[La]_{peak}$: 11.1 ± 2.4 mmol·L^{-1}
	SHU	1 × 6	25 m (12.5 + 12.5)	15 s	A	-	S_{best}: 5.17 ± 0.23 s; S_{avg}: 5.34 ± 0.23 s; S_{dec}: 3.2 ± 1.4%	-	△ CMJ^{AB}: 43.52 ± 1.48 to 40.37 ± 1.28 cm (-7.2%)	$B[La]_{peak}$: 12.2 ± 3.3 mmol·L^{-1}

Study	Exercise protocol						Outcomes			
	RST Mode	Sets × Reps	Distance / Duration	Rest Time	Rest Mode	I-set Rest	Performance	Perceptual	Neuromuscular	Physiological
Dal Pupo et al. [209]	STR	1 × 6	25 m	15 s	A	-	S_{best}: 3.73 ± 0.12 s; S_{avg}: 3.91 ± 0.15 s; S_{dec}: 4.7 ± 1.8%	-	-	-
	SHU	1 × 6	25 m (12.5 + 12.5)	15 s	A	-	S_{best}: 5.13 ± 0.22 s; S_{avg}: 5.30 ± 0.20 s; S_{dec}: 3.3 ± 0.9%	-	-	-
Daneshfar et al. [210]	SHU	1 × 10	30 m (15 + 15)	30 s	P	-	Test, S_{best}: 6.35 ± 0.08 s; S_{total}: 68.97 ± 0.23 s; S_{dec}: 9.1 ± 1.1%<br>Retest, S_{best}: 6.30 ± 0.08 s; S_{total}: 69.25 ± 0.24 s; S_{dec}: 9.3 ± 1.1%	CR10: 8.8 ± 0.1 au	-	$B[La]^{3'}$: 10.0 ± 0.1 mmol·L^{-1}
Dardouri et al. [211]	SHU	1 × 10	30 m (15 + 15)	30 s	P	-	S_{best}: 6.15 ± 0.25 s; S_{total}: 63.90 ± 2.50 s; S_{dec}: 4.1 ± 1.4%	-	-	$B[La]^{3'}$: 14.8 ± 0.4 mmol·L^{-1}
De Andrade et al. [212]	STR	1 × 6	35 m	10 s	P	-	S_{best}: 4.43 ± 0.17 s; S_{avg}: 4.91 ± 0.23 s; S_{total}: 29.45 ± 1.39 s; S_{dec}: 11.3 ± 7.6%	-	-	$B[La]_{peak}$: 13.7 ± 2.4 mmol·L^{-1}
Delextrat et al. [213]	SHU	1 × 10	30 m (15 + 15)	30 s	P	-	M, S_{total}: 58.40 ± 2.80 s; S_{dec}: 4.3 ± 0.4%; F, S_{total}: 63.50 ± 2.20 s; S_{dec}: 3.6 ± 0.9%	-	-	-
Delextrat et al. [214]	SHU	1 × 6	20 m (10 + 10)	On 20 s (~15 s)	P	-	S_{total}: 29.00 ± 2.10 s; S_{dec}: 4.0 ± 2.7%	-	-	-
Delextrat et al. [175]	SHU	1 × 10	30 m (15 + 15)	30 s	P	-	M, S_{total}: 58.01± 3.01 s; S_{dec}: 4.3 ± 1.5%; F, S_{total}: 63.34 ± 2.38 s; S_{dec}: 3.6 ± 0.3%	-	-	-
Dellal et al. [61]	STR	1 × 10	20 m	30 s	A^{K}	-	-	-	-	HR_{peak}:191 b·min^{-1} (% HRmax: 91%)
	STR	1 × 10	30 m	30 s	A^{K}	-	-	-	-	HR_{peak}:198 b·min^{-1} (% HRmax: 95%)
	STR	1 × 15	20 m	30 s	A^{K}	-	-	-	-	HR_{peak}:198 b·min^{-1}; (% HRmax: 95%)
Dellal & Wong [100]	MD^{X}	1 × 10	20 m	25 s	A^{K}	-	U17, S_{best}: 5.39 ± 0.03 s; S_{avg}: 5.47 ± 0.04 s; S_{total}: 32.76 ± 0.24 s; S_{dec}: 1.4 ± 0.6%;<br>U19, S_{best}: 5.34 ± 0.03 s; 5.39 ± 0.04 s; S_{total}: 32.25 ± 0.26 s; S_{dec}: 1.0 ± 0.4%;<br>PRO, S_{best}: 5.31 ± 0.05 s; S_{avg}: 5.37 ± 0.07 s; S_{total}: 32.22 ± 0.42 s; S_{dec}: 1.2 ± 0.5%	-	-	-

Study	Exercise protocol						Outcomes			
	RST Mode	Sets × Reps	Distance / Duration	Rest Time	Rest Mode	I-set Rest	Performance	Perceptual	Neuromuscular	Physiological
Dent et al. [131]	STR	4 × 6	30 m	On 30 s (~25 s)	A^K	7 min P	M, S_{best}: set 1, 4.29 ± 0.05 s; set 2, 4.35 ± 0.02 s; set 3, 4.45 ± 0.10 s; set 4, 4.49 ± 0.11 s;<br>S_{avg}: set 1, 4.47 ± 0.9 s; set 2, 4.54 ± 0.12 s; set 3, 4.60 ± 0.13 s; set 4, 4.54 ± 0.12 s;<br>S_{dec}: set 1, 4.7 ± 1.4%; set 2, 4.9 ± 1.4%; set 3, 5.4 ± 2.0%; set 4, 4.3 ± 1.1%<br>F, S_{best}: set 1, 4.74 ± 0.18 s; set 2, 4.87 ± 0.14 s; set 3, 4.96 ± 0.27 s; set 4, 4.97 ± 0.22 s<br>S_{avg}: set 1, 5.09 ± 0.21 s; set 2, 5.17 ± 0.31 s; set 3, 5.24 ± 0.27 s; set 4, 5.23 ± 0.31 s;<br>S_{dec}: set 1, 7.1 ± 2.1%; set 2, 6.6 ± 2.8%; set 3, 7.2 ± 1.3%; set 4, 7.2 ± 2.8%	-	-	M: set 1, △ $B[La]^{3'}$: 0.9 ± 0.4 to 10.0 ± 1.6 mmol·L^{-1}; set 2, $B[La]^{3'}$: 11.9 ± 2.9 mmol·L^{-1}; set 3, 11.6 ± 3.3 mmol·L^{-1}; set 4, 11.6 ± 4.0 mmol·L^{-1};<br>HR_{post}: set 1, 179 ± 20 b·min^{-1}; set 2, 175 ± 38 b·min^{-1}, set 3, 188 ± 10 b·min^{-1}; set 4, 189 ± 10 b·min^{-1};<br>F: set 1, △ $B[La]^{3'}$: 0.8 ± 0.3 to 10.0 ± 3.5 mmol·L^{-1}; set 2, $B[La]^{3'}$: 12 ± 3.6 mmol·L^{-1}; set 3, 12.0 ± 3.3 mmol·L^{-1}; set 4, 12.2 ± 3.7 mmol·L^{-1}<br>HR_{post}: set 1, 189 ± 9 b·min^{-1}; set 2, 190 ± 8 b·min^{-1}; set 3, 191 ± 6 b·min^{-1}; set 4, 190 ± 8 b·min^{-1}
Donghi et al. [215]	SHU	1 × 6	40 m (20 + 20)	20 s	P	-	-	CR10: 5 ± 1.2 au	-	-
Doyle et al. [216]	STR	1 × 6	20 m	On 15 s (~12 s)	A^W	-	S_{best}: 3.43 ± 0.16 s; S_{total}: 21.42 ± 0.97 s; S_{dec}: 4.4 ± 0.3%	-	-	-
Dupont et al. [217]	STR	1 × 7	30 m	20 s	A	-	S_{avg}: 4.60 ± 0.14 s	-	-	-
Dupont et al. [86]	STR	1 × 15	40 m	25 s	A^Z	-	S_{avg}: 6.41 ± 0.31 s; S_{dec}: 8.6 ± 3.2%	-	-	$B[La]^{3'}$: 13.8 ± 3.1 mmol·L^{-1};<br>VO_{2avg}: 60.5 ± 4.3 ml·min^{-1}·kg^{-1}
Eliakim et al. [124]	STR	1 × 12	20 m	On 20 s (~ 17 s)	P	-	S_{best}: 3.23 ± 0.17 s; S_{avg}: 3.24 ± 0.04 s; S_{total}: 38.91 ± 0.52 s; S_{dec}: 2.3 ± 0.6	CR10: 7 ± 1 au	-	HR_{avg}: 177 ± 6 b·min^{-1}<br>HR_{post}: 181 ± 4 b·min^{-1}
Elias et al. [218]	STR	1 × 6	20 m	On 30 s (~27 s)	P	-	PAS, S_{total}: 18.53 ± 0.28 s;<br>COL, S_{total}: 18.62 ± 0.46 s;<br>CWT, S_{total}: 18.63 ± 0.45 s	-	-	-
Elias et al. [219]	STR	1 × 6	20 m	On 30 s (~27 s)	P	-	PAS, S_{total}: 18.66 ± 0.37 s;<br>COL, S_{total}: 18.50 ± 0.47 s;<br>CWT, S_{total}: 18.68 ± 0.39 s	-	-	-
Eniseler et al. [220]	SHU	1 × 6	40 m (20 + 20)	20 s	P	-	RS, S_{best}: 6.75 ± 0.19 s; S_{avg}: 7.13 ± 0.17 s; S_{dec}: 5.5 ± 0.8%<br>SSG, S_{best}: 6.73 ± 0.19 s; S_{avg}: 7.12 ± 0.17 s; S_{dec}: 5.8 ± 1.1%	-	-	-

Study	Exercise protocol						Outcomes			
	RST Mode	Sets × Reps	Distance / Duration	Rest Time	Rest Mode	I-set Rest	Performance	Perceptual	Neuromuscular	Physiological
Eryilmaz & Kaynak [221]	STR	1 × 10	20 m	20 s	A^K	-	S_{best}: 2.97 ± 0.10 s; S_{avg}: 3.21 ± 0.10 s; S_{dec}: 8.0 ± 2.7%	-	-	-
Eryilmaz et al. [37]	STR	1 × 10	20 m	20 s	A^K	-	S_{avg}: 4.28 ± 0.10 s	-	-	-
Essid et al. [222]	SHU	1 × 6	30 m (15 + 15)	On 20 s (~14 s)	P	-	S_{best}: 6.19 ± 0.03 s; S_{avg}: 6.78 ± 0.03; S_{dec}: 8.7 ± 0.0%	-	-	-
Farjallah et al. [223]	SHU	1 × 6	40 m (20 + 20)	20 s	P	-	INT, S_{avg}: 7.18 ± 0.23 s; PLA, S_{avg}: 7.34 ± 0.03 s	-	-	-
Figueira et al. [119]	SHU	3 × 10	30 m (15 + 15)	30 s	P	5 min P	S_{total}: 59.22 ± 2.10 s; S_{dec}: 3.6 ± 1.6%	-	-	$B[La]^{3'}$: 13.0 ± 2.3 $mmol \cdot L^{-1}$; HR_{peak}: 174 ± 7 $b \cdot min^{-1}$
	STR	3 × 20	15 m	15 s	P	5 min P	S_{total}: 53.66 ± 1.56 s; S_{dec}: 4.9 ± 2.1%	-	-	$B[La]^{3'}$: 8.5 ± 3.4 $mmol \cdot L^{-1}$ HR_{peak}: 174 ± 7 $b \cdot min^{-1}$
Freitas et al. [227]	SHU	1 × 10	30 m (15 + 15)	30 s	P	-	S_{total}: 57. 50 ± 2.89 s; S_{dec}: 2.9 ± 1.0%	-	-	-
Fornasier-Santos et al. [224]	STR	1 × 10	40 m	On 30 s (~25 s)	P	-	-	HYP, CR10: 9.2 ± 0.7 au NOR, CR10: 9.2 ± 0.7 au	-	HYP: 13.7 ± 4.3 $mmol \cdot L^{-1}$ NOR: 13.0 ± 4.2 $mmol \cdot L^{-1}$
	STR	2 × 8	40 m	NR	P	3 min P	-	CR10: 8.3 ± 0.5	-	10.2 ± 3.3 $mmol \cdot L^{-}$
Fort-Vanmeerhaeghe et al. [225]	SHU	1 × 10	30 m (15 + 15)	30 s	P	-	S_{best}: 6.20 ± 0.20 s; S_{avg}: 6.34 ± 0.19 s	-	-	-
Fortin & Billaut [226]	STR	1 × 12	20 m	20 s	A^K	-	Sham, S_{best}: 3.07 ± 0.13 s; S_{total}: 39.69 ± 1.34 s; INT, S_{best}: 3.05 ± 0.08 s; S_{total}: 39.79 ± 1.65 s	-	-	-
Gabbett [228]	STR	1 × 6	20 m	On 15 s (~12 s)	A^J	-	S_{total}: 21.50 ± 1.20 s; S_{dec}: 5.6 ± 1.6%	-	-	$B[La]_{post}$: 9.3 ± 2.0 $mmol \cdot L^{-1}$ HR_{peak}: 182 ± 6 $b \cdot min^{-1}$
Gabbett et al. [89]	STR	1 × 12	20 m	On 20 s (~17 s)	P	-	S_{total}: 38.70 ± 2.30 s	-	-	-
Gabbett et al. [229]	STR	1 × 12	20 m	On 20 s (~17 s)	P	-	ST, S_{total}: 38.30 ± 2.80 s; N-ST, S_{total}: 38.90 ± 3.20; N-SEL, S_{total}: 39.10 ± 3.30	-	-	-

Study	Exercise protocol						Outcomes			
	RST Mode	Sets × Reps	Distance / Duration	Rest Time	Rest Mode	I-set Rest	Performance	Perceptual	Neuromuscular	Physiological
Gabbett et al. [230]	STR	1 × 6	20 m	On 15 s (~12 s)	A[J]	-	INT, S_{total}: 21.16 ± 1.06 s; CON, S_{total}: 20.71 ± 0.52 s	-	-	-
Galvin et al. [231]	STR	1 × 10	30 m	30 s	P	-	HYP, S_{total}: 32.20 ± 1.10 s; S_{dec}: 4.0 ± 3.0%; NOR, S_{total}: 32.70 ± 1.20 s; S_{dec}: 5.1 ± 3 .9%	-	-	-
Galy et al. [177]	STR	1 × 6	25 m	25 s	A[K]	-	MG, S_{best}: 3.77 ± 0.19 s; S_{avg}: 3.99 ± 0.17 s; S_{total}: 23.96 ± 1.05 s; S_{dec}: 5.9 ± 3.1%; N-MG, S_{best}: 3.92 ± 0.19 s; S_{avg}: 4.09 ± 0.17 s; S_{total}: 24.55 ± 1.01 s; S_{dec}: 4.4 ± 1.8%	-	-	-
	SHU	1 × 6	25 m (12.5 + 12.5)	25 s	A[K]	-	MG, S_{best}: 5.29 ± 0.19 s; S_{avg}: 5.47 ± 0.19 s; S_{total}: 32.79 ± 1.14 s; S_{dec}: 3.4 ± 1.0%; N-MG, S_{best}: 5.31 ± 0.18 s; S_{avg}: 5.53 ± 0.15 s; S_{total}: 33.21 ± 0.92 s; S_{dec}: 4.3 ± 0.8%	-	-	-
Gantois et al. [232]	STR	1 × 6	30 m	20 s	P	-	S_{best}: 4.59 ± 0.24 s; S_{avg}: 4.82 ± 0.31 s; S_{total}: 27.60 ± 6.77 s; S_{dec}: 5.3 ± 2.9%	-	-	-
Gantois et al [14]	STR	1 × 6	30 m	20 s	P	-	RS, S_{best}: 4.56 ± 0.24 s; S_{avg}: 4.83 ± 0.38 s; S_{total}: 29.00 ± 2.30; S_{dec}: 6.4 ± 3.5%; CON, S_{best}: 4.64 ± 0.24 s; S_{avg}: 4.87 ± 0.22; S_{total}: 29.08 ± 1.56 s; S_{dec}: 4.1 ± 1.8%	-	-	-
Gantois et al. [233]	STR	1 × 6	30 m	20 s	P	-	S_{best}: 4.58 ± 0.21 s; S_{avg}: 4.84 ± 0.31; S_{total}: 29.00 ± 1.91 s; S_{dec}: 7.6 ± 5.8%	-	-	-
García-Unanue et al. [169]	STR	1 × 7	30 m	20 s	P	-	ELT, S_{avg}: 4.37 ± 0.15 s; S_{dec}: 4.2 ± 1.4%; AM, S_{avg}: 4.67 ± 0.18 s; S_{dec}: 6.4 ± 2.2%	-	ELT, △ CMJ [AA]: 35.7 ± 6.0 to 34.0 ± 4.3 cm (-4.8%) AM, △ CMJ [AA]: 33.8 ± 4.2 to 31.8 ± 3.6 cm (-5.9%)	-
Gatterer et al. [234]	SHU	1 × 6	40 m (20 + 20)	20 s	P	-	NOR, S_{best}: 7.18 ± 0.24 s; S_{avg}: 7.60 ± 0.19 s; S_{dec}: 5.8 ± 1.9%; HYP, 7.28 ± 0.21 s; S_{avg}: 7.66 ± 0.32 s; S_{dec}: 5.2 ± 2.6%	-	-	-

Study	Exercise protocol						Outcomes			
	RST Mode	Sets × Reps	Distance / Duration	Rest Time	Rest Mode	I-set Rest	Performance	Perceptual	Neuromuscular	Physiological
Gharbi et al. [83]	SHU	1 × 2	30 m (15 + 15)	30 s	P	-	S_{best}: 6.26 ± 0.24 s; S_{total}: 12.63 ± 0.47 s; S_{dec}: 1.0 ± 0.7%	-	-	$B[La]^{3'}$: 1.8 ± 0.6 to 5.7 ± 1.2 mmol·L^{-1}
	SHU	1 × 3	30 m (15 + 15)	30 s	P	-	S_{best}: 6.18 ± 0.23 s; S_{total}: 18.75 ± 0.61 s; S_{dec}: 1.5 ± 1.0%	-	-	$B[La]^{3'}$: 1.8 ± 0.6 to 9.4 ± 1.7 mmol·L^{-1}
	SHU	1 × 4	30 m (15 + 15)	30 s	P	-	S_{best}: 6.17 ± 0.21 s; S_{total}: 25.05 ± 0.81 s; S_{dec}: 2.0 ± 1.1%	-	-	$B[La]^{3'}$: 1.8 ± 0.6 to 9.6 ± 1.9 mmol·L^{-1}
	SHU	1 × 5	30 m (15 + 15)	30 s	P	-	S_{best}: 6.29 ± 0.20 s; S_{total} 32.36 ± 1.23 s; S_{dec}: 2.6 ± 1.4%	-	-	$B[La]^{3'}$: 1.8 ± 0.6 to 10.5 ± 2.6 mmol·L^{-1};
	SHU	1 × 9	30 m (15 + 15)	30 s	P	-	S_{best}: 6.28 ± 0.23 s; S_{total}: 58.68 ± 2.38 s; S_{dec}: 3.9 ± 1.3%	-	-	$B[La]^{3'}$: 1.8 ± 0.6 to 12.6 ± 2.3 mmol·L^{-1};
	SHU	1 × 10	30 m (15 + 15)	30 s	P	-	S_{best}: 6.23 ± 0.23 s; S_{total}: 64.96 ± 2.57 s; S_{dec}: 4.5 ± 1.4%	-	-	$B[La]^{3'}$: 1.8 ± 0.6 to 12.7 ± 1.0 mmol·L^{-1}
Gharbi et al. [235]	SHU	1 × 10	30 m (15 + 15)	30 s	P	-	S_{best}: 6.10 ± 0.20 s; S_{total}: 63.20 ± 2.20 s; S_{dec}: 3.5 ± 1.1%	-	-	$B[La]^{3'}$: 15.3 ± 2.1 mmol·L^{-1}
Gibson et al. [101]	MD^{A}	1 × 6	40 m	25 s	P	-	S_{best}: 7.11 ± 0.25 s; S_{total}: 44.40 ± 1.62 s; S_{dec}: 3.6 ± 1.2%	-	-	-
Girard et al. [236]	STR	1 × 6	35 m	10 s	P	-	S_{avg}: 5.36 ± 0.29 s; S_{dec}: 8.6 ± 2.8%	-	-	-
Girard et al. [149]	STR	1 × 6	20 m	20 s	P	-	S_{avg}: 3.23 ± 0.13 s; S_{dec}: 2.8 ± 1.7%	-	Δ*L*: 13.6 ± 2.1 to 15.4 ± 2.7 cm; Δz: 1.7 ± 0.4 to 2.2 ± 0.4 cm; $F_z$max: 2.0 ± 0.28 to 2.1 ± 0.26 N; K_{vert}: 120 ± 9.3 to 97 ± 5.2 kN·m^{-1}; K_{leg}: 15.0 ± 10.0 to 13.7 ± 7.0 kN·m^{-1}	-
González-Frutos et al. [237]	STR	1 × 6	30 m	30 s	A^{K}		S_{avg}: 4.89 ± 0.07 s	-	-	-
Gonzalo-skok et al. [102]	SHU	1 × 6	40 m (20 + 20)	20 s	P	-	INT, S_{best}: 7.16 ± 0.23 s; S_{avg}: 7.52 ± 0.23 s; S_{dec}: 5.1 ± 1.8%; CON, S_{best}: 7.17 ± 0.24 s; 7.50 ± 0.24 s; S_{dec}: 4.6 ± 1.8%	-	-	-

Study	Exercise protocol						Outcomes			
	**RST Mode**	**Sets × Reps**	**Distance / Duration**	**Rest Time**	**Rest Mode**	**I-set Rest**	**Performance**	**Perceptual**	**Neuromuscular**	**Physiological**
	MD[G]	1 × 5	25 m (5 m per turn)	20 s	P	-	INT, S_{best}: 6.58 ± 0.21 s; S_{avg}: 6.86 ± 0.25 s; S_{dec}: 2.0 ± 0.7; CON, S_{best}: 6.56 ± 0.3; S_{avg}: 6.84 ± 0.22 s; S_{dec}: 2.3 ± 1.5%	-	-	-
Goodall et al. [238]	STR	1 × 12	30 m	30 s	P	-	S_{best}: 4.23 ± 0.13 s; S_{avg}: 4.68 ± 0.08 s;	-	-	△ $B[La]^{3'}$: 3.1 ± 1.4 to 12.8 ± 3.0, $mmol·L^{-1}$; $B[La]_{post}$ sprint 1: 2.7 $mmol·L^{-1}$; sprint 3: 4.8 $mmol·L^{1}$; sprint 5: 7.2 $mmol·L^{-1}$; sprint 7: 9.1 $mmol·L^{-1}$; sprint 9: 10.4 $mmol·L^{-1}$; sprint 11: 11.6 $mmol·L^{-1}$
Hamlin et al. [239]	STR	1 × 10	40 m	On 30 s (~24 s)	P	-	CWT, S_{avg}: 6.36 ± 0.40 s; ARC: 6.38 ± 0.50 s	-	-	CTWI, $B[La]^{3'}$: 13.6 ± 2.6 $mmol·L^{-1}$; HR_{avg}: 171 ± 9 $b·min^{-1}$; AR, $B[La]^{3'}$: 14.2 ± 2.3 $mmol·L^{-1}$; HR_{avg}: 173 ± 11 $b·min^{-1}$
Hamlin et al. [240]	STR	1 × 8	20 m	On 20 s (~17 s)	P	-	NOR, S_{total}: 27.40 ± 3.20; S_{dec}: 3.5 ± 1.2%; HYP, S_{total}: 27.50 ± 3.90 s; S_{dec}: 3.5 ± 1.3%	-	-	-
Hammami et al. [241]	SHU	1 × 6	40 m (20 + 20)	20 s	P	-	INT, S_{best}: 7.13 ± 0.32 s; S_{avg}: 7.39 ± 0.33 s, S_{total}: 44.4 ± 2.0 s; S_{dec}: 3.7 ± 1.4%<br>INT, S_{best}: 7.21 ± 0.13 s; S_{avg}: 7.44 ± 0.15 s, S_{total}: 44.6 ± 0.9 s; S_{dec}: 3.1 ± 1.7%	-	-	-
Haugen et al. [62]	STR	1 × 12	20 m	60 s	P	-	INT, S_{best}: 3.11 ± 0.17 s; S_{avg}: 3.16 ± 0.17 s<br>CON, S_{best}: 3.02 ± 0.17 s; S_{avg}: 3.07 ± 0.17 s	-	-	INT, $B[La]_{post}$: 3.8 ± 1.3 $mmol·L^{-1}$; HR_{peak} (% HRmax): 85 ± 4%<br>CON, $B[La]_{post}$: 3.5 ± 1.4 $mmol·L^{-1}$; HR_{peak} (% HRmax): 86 ± 4%
Haugen et al. [128]	STR	1 × 15	20 m	60 s	P	-	S_{best}: 2.94 ± 0.15; S_{avg}: 2.98 ± 0.15	CR10: 3.8 ± 1.2 au	-	$B[La]_{post}$: 4.4 ± 1.8 $mmol·L^{-1}$
Hermassi et al. [242]	STR	1 × 6	30 m	On 20 s (~16 s)	P	-	S_{best}: 4.42 ± 0.14; S_{avg}: 4.57 ± 0.12; S_{total}: 27.40 ± 0.70 s; 3.4 ± 1.6%	-	-	-
	SHU	1 × 6	30 m (15 + 15)	On 20 s (~14 s)	P	-	S_{best}: 5.97 ± 0.36; S_{avg}: 6.23 ± 0.25; S_{total}: 37.40 ± 1.50 s; S_{dec}: 4.5 ± 3.3%	-	-	-

Study	Exercise protocol						Outcomes			
	RST Mode	Sets × Reps	Distance / Duration	Rest Time	Rest Mode	I-set Rest	Performance	Perceptual	Neuromuscular	Physiological
Higham et al. [90]	STR	1 × 6	30 m	On 20 s (~16 s)	P	-	S_{total}: 24.76 ± 0.62 s	-	-	-
Hollville et al. [243]	STR	1 × 6	20 m	On 20 s (~17 s)	P	-	S_{best}: 3.14 s± 0.12 s; S_{total}: 19.30 ± 0.60 s; S_{dec}: 2.4 ± 1.3%	CR10: 4.5 ± 1.6 au		HR_{post} (% HRmax): 88 ± 5%
Howatson et al. [35]	STR	1 × 15	30 m	60 s	P	-	S_{best}: 4.33 ± 0.21 s; S_{avg}: 4.49 ± 0.09 s; S_{dec}: 4.5 ± 1.5%	-	-	△ CK 24 h: 158 ± 56 to 776 ± 312 u·L^{-1} (385%)
Iaia et al. [120]	STR	1 × 15	40 m	30 s	P	-	SEP, S_{total}: 86.09 ± 6.30 s; S_{dec}: 5.0 ± 2.3%; SEM, S_{total}: 83.81 ± 2.37 s; S_{dec}: 4.1 ± 1.3%	-	-	-
Iaia et al. [19]	STR	1 × 6	5 s (~30 m)	15 s	P	-	-	-	-	$B[La]_{post}$: 3.1 ± 0.8 to 9.3 ± 1.6 mmol·L^{-1}
	STR	1 × 6	5 s (~30 m)	30 s	P	-	-	-	-	$B[La]_{post}$: 3.5 ± 1.1 to 6.6 ± 1.8 mmol·L^{-1}
	STR	1 × 15	40 m	30 s	P	-	RS15, S_{total}: 92.91 ± 4.66 s; S_{dec}: 5.9 ± 2.2%; RS30, S_{total}: 91.45 ± 4.35 s; S_{dec}: 5.2 ± 2.1%	-	-	-
Impellizzeri et al. [170]	SHU	1 × 6	40 m (20 + 20)	20 s	P	-	Test, S_{best}: 6.90 ± 0.09 s; S_{avg}: 7.20 ± 0.11; S_{dec}: 4.3 ± 1.2%; Retest, S_{best}: 6.92 ± 0.10 s; S_{avg}: 7.19 ± 0.14 s; S_{dec}: 3.8 ± 1.4%	-	-	-
Impellizzeri et al. [170]	SHU	1 × 6	40 m (20 + 20)	20 s	P	-	PRE, S_{best}: 6.94 ± 0.15 s; S_{avg}: 7.32 ± 0.13 s; S_{dec}: 5.4 ± 2.2%; ELY, S_{best}: 6.87 ± 0.17 s; S_{avg}: 7.16 ± 0.15 s; S_{dec}: 4.3 ± 1.7%; MID, S_{best}: 6.93 ± 0.15 s; S_{avg}: 7.22 ± 0.14 s; S_{dec}: 4.2 ± 1.6%; END, S_{best}: 6.92 ± 0.15 s; S_{avg}: 7.20 ± 0.13 s; S_{dec}: 4.0 ± 1.7%	-	-	-
Impellizzeri et al. [170]	SHU	1 × 6	40 m (20 + 20)	20 s	P	-	PRO, S_{best}: 6.88 ± 0.19 s; S_{avg}: 7.12 ± 0.17 s; S_{dec}: 3.3 ± 1.5%; M-PRO, S_{best}: 6.83 ± 0.18 s; S_{avg}: 7.20 ± 0.19 s; S_{dec}: 5.1 ± 1.8%; AM, S_{best}: 7.08 ± 0.23 s; S_{avg}: 7.55 ± 0.25 s; S_{dec}: 6.1 ± 2.0%	-	-	-
Ingebrigtsen et al. [244]	STR	1 × 7	35 m	25 s	A	-	S_{avg}: 5.25 ± 0.19 s	-	-	-

Study	Exercise protocol						Outcomes			
	RST Mode	Sets × Reps	Distance / Duration	Rest Time	Rest Mode	I-set Rest	Performance	Perceptual	Neuromuscular	Physiological
Ingebrigtsen et al. [171]	STR	1 × 7	35 m	25 s	A	-	EL, S_{avg}:.5.24 ± 0.24 s; S_{dec}: 8.3 ± 5.3%; S-EL, S_{avg}: 5.26 ± 0.18 s; S_{dec}: 6.4 ± 3.7%	-	-	EL, HR_{peak}: 179 ± 9; S-EL, HR_{peak}: 188 ± 7
Iacono et al. [42]	SHU	1 × 6	40 m (20 + 20)	On 20 s (~14 s)	P	-	SSG, S_{best}: 5.30 ± 0.15 s; S_{avg}: 5.48 ± 0.15; S_{dec}: 3.4 ± 0.5% RS, S_{best}: 5.31 ± 0.22 s; S_{avg}: 5.48 ± 0.18; S_{dec}: 3.3 ± 1.0%	-	-	-
Izquierdo et al. [140]	STR	1 × 6	15 m	60 s	P	-	PLA, S_{avg}: 2.45 ± 0.06 s; INT, S_{avg}: 2.39 ± 0.06 s	-	-	-
Jiménez-Reyes et al. [246]	STR	1 × 10	40 m	30 s	P	-	-	-	△ sprint 1–10: V_0: ↓ 15.1 ± 1.3%, F_0: ↓ 5.9 ± 4.5%; P_0: ↓ 20.1 ± 3.3%, RF: ↓ 6.8 ± 2.0%, D_{RF}: ↓ 14.0 ± 6.0	-
Johnston & Gabbett [40]	STR	1 × 12	20 m	On 20 s (~17 s)	A^W	-	S_{best}: 3.09 ± 0.04 s; S_{avg}: 3.49 ± 0.14 s; S_{total}: 41.89 ± 0.20 s; S_{dec}: 11.4 ± 4.5%	6–20: 12.3 ± 1.2 au	-	HR_{peak}: 166 ± 9 b·min^{-1} HR_{avg}: 154 ± 9 b·min^{-1}
Joo [110]	MD^A	1 × 7	34.2 m	25 s	A	-	S_{total}: 45.7 ± 2.6 s	-	-	-
Jorge et al. [247]	MD^A	1 × 7	34.2 m	25	A^L	-	U20 ELY, S_{avg}: 6.68 ± 0.16 s; S_{dec}: 4.3 ± 1.0%; U20 MID, S_{avg}: 6.20 ± 0.13 s; S_{dec}: 4.1 ± 1.0%; U20 END, S_{avg}: 6.40 ± 0.14 s; S_{dec}: 4.0 ± 1.0%; U17 ELY, S_{avg}: 7.01 ± 0.21 s; S_{dec}: 5.3 ± 2.0%; U17 MID, S_{avg}: 6.25 ± 0.16 s; S_{dec}: 4.5 ± 1.8%; U17 END: S_{avg}: 6.32 ± 0.13 s; S_{dec}: 3.8 ± 1.3%	-	-	-
Kaplan [109]	MD^A	1 × 7	34.2 m	25	A^L	-	S_{best}: 7.37 ± 0.26 s; S_{avg}: 7.57 ± 0.25 s; S_{dec}: 4.4 ± 1.7%	-	-	-
Keir et al. [25]	STR	1 × 6	35 m	10 s	P	-	-	-	-	$B[La]_{peak}$: 14.8 ± 2.8 mmol·L^{-1}; VO_{2avg}: 45.6 ± 9.4 ml·min^{-1}·kg^{-1}; HR_{peak}: 182 ± 10 b·min^{-1}
Keogh [172]	STR	1 × 6	40 m	On 30 s (~25 s)	A^K	-	REP, S_{dec}: 13.1 ± 1.0; Club, S_{dec}: 12.7 ± 1.4	-	-	-

Study	Exercise protocol						Outcomes			
	RST Mode	Sets × Reps	Distance / Duration	Rest Time	Rest Mode	I-set Rest	Performance	Perceptual	Neuromuscular	Physiological
Kilduff et al. [248]	SHU	1 × 6	40 m	20 s	P	-	S_{best}: 6.72 ± 0.16 s; S_{avg}: 7.01 ± 0.16 s; S_{total}: 42.09 ± 0.94 s	-	-	-
Klatt et al. [36]	SHU	4 × 6	40 m (20 + 20)	30 s	P	5 min P	U20, S_{best}: 6.99 ± 0.17 s; S_{avg}: 7.39 ± 0.26 s<br>SEN, S_{best}: 7.12 ± 0.29 s; S_{avg}: 7.65 ± 0.32 s;	U20, CR10: 8.7 ± 1.2 au<br>SEN, CR10: 8.3 ± 2.0 au	U20, △CMJ^{AC}: 37.5 ± 5.1 cm to 39 ± 4.7 cm (4.0%)<br>SEN, △CMJ^{AC}: 31.6 ± 3.9 cm to 34.0 ± 3.9 cm (7.6%)	$B[La]_{post}$: 10.2 ± 2.6 $mmol \cdot L^{-1}$<br><br>U20, △ CK 24 h: 285 ± 155 to 354 ± 134 $u \cdot L^{-1}$ (24%)<br>SEN, △ CK 24 h: 214 ± 82 to 443 ± 207 $u \cdot L^{-1}$ (47%)
Krakan et al. [249]	STR	1 × 6	25 m	25 s	P	-	RS, S_{best}: 3.78 ± 0.08 s; S_{avg}: 3.97 ± 0.10 s; S_{dec}: 5.0 ± 3.2%<br>PLY, S_{best}: 3.74 ± 0.11 s; S_{avg}: 3.96 ± 0.14 s, S_{dec}: 5.8 ± 0.1	RS, CR10: 7.3 ± 1.5 au<br>PLY, CR10: 8 ± 1.1 au	-	RS, $B[La]_{post}$: 13.1 ± 2.5 $mmol \cdot L^{-1}$<br>PLY, $B[La]_{post}$: 14.8 ± 2.3 $mmol \cdot L^{-1}$
Krueger et al. [250]	STR	1 × 6	30 m	On 25 s (~21 s)	P	-	CWI, S_{total}: 26.23 ± 1.06 s;<br>CON, S_{total}: 26.05 ± 0.69 s	-	-	-
Lakomy et al. [78]	STR	1 × 6	40 m	30 s	A^{W}	-	S_{avg}: 5.97 ± 0.40 s; S_{dec}: 4.2 ± 2.4%	-	-	-
	STR	1 × 6	40 m	30 s	P^{R}	-	S_{avg}: 6.03 ± 0.52 s; S_{dec}: 3.9 ± 1.3%	-	-	-
Lapointe et al. [251]	STR	1 × 12	30 m	20 s	A^{K}	-	CON, S_{best}: 4.83 ± 0.36 s; S_{avg}: 5.18 ± 0.51 s; S_{dec}: 7.1 ± 3.1%;<br>INT, S_{best}: 4.80 ± 0.35 s; S_{avg}: 5.16 ± 0.47 s; S_{dec}: 7.3 ± 3.2%	CON, CR10: 8 ± 1.2 au<br>INT, CR10: 7.5 ± 1.1 au	-	CON, $B[La]^{1'}$: 14.0 ± 2.4 $mmol \cdot L^{-1}$;<br>INT, $B[La]^{1'}$: 13.5 ± 1.5 $mmol \cdot L^{-1}$
Le Rossignol et al. [173]	STR	1 × 6	30 m	On 20 s (~16 s)	P	-	SEL, S_{total}: 25.26 ± 0.55 s;<br>N-SEL, S_{total}: 25.92 ± 0.8 s	-	-	-
Little & Williams [121]	STR	1 × 15	40 m	$1{:}6^{N}$ (~34 s)	P	-	S_{avg}: 5.73 ± 0.07 s	6–20: 14.4 ± 1.0 au	-	$B[La]^{2'}$: 9.6 ± 0.6 $mmol \cdot L^{-1}$; HR_{avg} (% HR_{max}): 85.8 ± 0.8%
	STR	1 × 15	40 m	$1{:}4^{N}$ (~22 s)	P	-	S_{avg}: 5.93 ± 0.19 s	6–20: 17.1 ± 0.4 au	-	$B[La]^{2'}$: 14.1 ± 1.0 $mmol \cdot L^{-1}$; HR_{avg} (% HR_{max}): 89.2 ± 1.9%
	STR	1 × 40	15 m	$1{:}6^{N}$ (~16 s)	P	-	S_{avg}: 2.59 ± 0.05 s	6–20: 17.3 ± 0.5 au	-	$B[La]^{2'}$: 8.8 ± 1.1 $mmol \cdot L^{-1}$; HR_{avg} (% HR_{max}) : 86.8 ± 1.0%
	STR	1 × 40	15 m	$1{:}4^{N}$ (~10 s)	P	-	S_{avg}: 2.65 ± 0.10 s	6–20: 18.8 ± 0.4 au	-	$B[La]^{2'}$: 13.0 ± 1.7 $mmol \cdot L^{-1}$; HR_{avg} (% HR_{max}): 89.3 ± 1.2%

Study	Exercise protocol						Outcomes			
	RST Mode	Sets × Reps	Distance / Duration	Rest Time	Rest Mode	I-set Rest	Performance	Perceptual	Neuromuscular	Physiological
Lockie et al. [252]	STR	1 × 7	30 m	On 20 s (~16 s)	A[X]	-	FSH, S_{avg}: 32.08 ± 1.31 s; EXP, S_{avg}: 31.67 ± 0.76 s	-	-	-
Lockie et al. [253]	STR	1 × 6	20 m	On 15 s (~11s)	A[X]	-	S_{total}: 31.95 ± 1.06 s	-	-	-
Lockie et al. [254]	STR	1 × 7	20 m	On 20 s (~15s)	A[X]	-	S_{total}: 31.95 ± 1.06 s	-	-	-
Lombard et al. [255]	STR	1 × 6	30 m	On 25 s (~21 s)	A[X]	-	S_{total}: 26.77 ± 0.96 s	-	-	-
Madueno et al. [24]	SHU	1 × 12	20 m (15 + 5)	20 s	P	-	-	CR10: 6.5 ± 0.5 au	-	△ $B[La]_{post}$: 2.0 to 6.8 mmol·L^{-1}; △ $B[La]^{5'}$: 4.8 mmol·L^{-1}; VO_{2avg}: 33.3 ± 4.0 mL·kg^{-1}· min^{-1}; VO_{2avg} (% VO_{2max}): 73.1 ± 9.8%; HR_{avg}: 166 ± 8 b·min^{-1} (% HR_{max}: 83 ± 6%)
	SHU	1 × 12	20 m (15 + 5)	20 s	A[Z]	-	-	CR10: 6.0 ± 0.5 au	-	△ $B[La]_{post}$: 2.0 to 8.6 mmol·L^{-1}; $B[La]^{5'}$: 6.3 mmol·L^{-1}; VO_{2avg}: 37.7 ± 7.1 mL·kg^{-1}·min^{-1} (% VO_{2max}: 82.5 ± 14.9%); HR_{avg}: 173 ± 5 b·min^{-1} (% HR_{max}: 86 ± 2%)
Maggioni et al. [16]	SHU	3 × 6	40 m (20 + 20)	20 s	P	3 min P	-	CR10: 6.1 ± 2.7 au	-	-
Mancha-Triguero et al. [139]	STR	1 × 5	14 m	30 s	A	-	M, S_{best}: 2.48 ± 0.18 s; S_{avg}: 2.65 ± 0.16 s; S_{total}: 13.27 ± 0.83 s; F, S_{best}: 2.70 ± 0.16 s; S_{avg}: 2.99 ± 0.15 s; S_{total}: 14.98 ± 0.73 s	-	-	-
Marcelino et al. [256]	STR	1 × 12	20 m	20 s	A[k]	-	SSG 1, S_{best}: 3.20 ± 0.10 s; S_{avg}: 3.36 ± 0.10; S_{dec}: 5.3 ± 3.9%; SSG 2, S_{best}: 3.18 ± 0.07 s; S_{avg}: 3.37 ± 0.07 s; S_{dec}: 6.1 ± 3.3%	-	-	-
Matzenbacher et al. [257]	SHU	1 × 6	40 m (20 + 20)	20 s	P	-	PRE, S_{best}: 7.13 ± 0.26 s; S_{avg}: 7.49 ± 0.34 s; S_{dec}: 4.9 ± 1.7%; END, S_{best}:7.15 ± 0.24 s; S_{avg}: 7.42 ± 0.27 s; S_{dec}: 3.8 ± 1.9%	-	-	-

Study	Exercise protocol						Outcomes			
	RST Mode	Sets × Reps	Distance / Duration	Rest Time	Rest Mode	I-set Rest	Performance	Perceptual	Neuromuscular	Physiological
McGawley & Andersson [258]	STR	1 × 6	30 m	On 20 s (~16 s)	P	-	Condition 1, S_{best}: 27.70 ± 0.50 s; S_{dec}: 4.7 ± 1.6%; Condition 2, S_{best}: 26.70 ± 0.90 s; S_{dec}: 5.2 ± 1.1%	-	-	-
Meckel et al. [259]	STR	1 × 6	30 m	30 s	P	-	PRE, S_{total}: 22.50 ± 0.60 s; S_{dec}: 2.9 ± 0.3%; MID, S_{total}: 23.70 ± 0.63 s; S_{dec}: 2.3 ± 0.2%; END, S_{total}: 23.51 ± 0.62 s; S_{dec}: 2.2 ± 0.2%	-	-	-
Meckel et al. [260]	STR	1 × 12	20 m	On 20 s (~17 s)	P	-	S_{total}: 39.70 ± 0.60 s; S_{dec}: 5.0 ± 0.5%	CR10: 6.9 ± 0.4 au	-	△ $B[La]^{2'}$: 2.0 ± 0.1 to 8.8 ± 0.7 mmol·L^{-1}; HR_{peak}: 182 ± 2 b·min^{-1}
Meckel et al. [261]	STR	1 × 6	40 m	On 30 s (~24 s)	P	-	S_{best}: 5.60 ± 0.26 s; S_{total}: 35.10 ± 1.50 s; S_{dec}: 4.8 ± 1.9%	CR10: 4.9 ± 1.4 au	-	$B[La]^{2'}$: 11.3 ± 2.5 mmol·L^{-1}; HR_{peak}: 179 ± 8 b·min^{-1}
	STR	1 × 12	20 m	On 20 s (~17 s)	P	-	S_{best}: 3.10 ± 0.10 s; S_{total}: 38.80 ± 1.20 s; S_{dec}: 5.0 ± 2.0%	CR10: 4.0 ± 1.3 au	-	$B[La]^{2'}$: 10.5 ± 1.8 mmol·L^{-1}; HR_{peak}: 184 ± 8 b·min^{-1}
Meckel et al. [262]	STR	1 × 6	30 m	30 s	P	-	S_{total}: 27.71 ± 1.40 s; S_{dec}: 1.6 ± 0.7%	CR10: 5.4 ± 1.5 au	-	$B[La]^{2'}$: 10.1 ± 2.1 mmol·L^{-1}; HR_{peak}: 171 ± 7 b·min^{-1}
Meckel et al. [263]	STR	1 × 12	20 m	On 20 s (~17 s)	P	-	S_{total}: 37.80 ± 1.40 s; S_{dec}: 4.4 ± 1.5%	CR10: 5.2 ± 1.3 au	-	$B[La]^{2'}$: 6.7 ± 1.1 mmol·L^{-1}; HR_{peak}: 174 ± 9 b·min^{-1}
Michalsik et al. [264]	STR	1 × 7	30 m	25 s	A^{Q}	-	S_{best}: 4.09 ± 0.12 s; S_{avg}: 4.30 ± 0.13 s	-	-	-
Mohr et al. [265]	STR	1 × 5	30 m	25 s	A^{K}	-	S_{avg}: 4.58 ± 0.15 s	-	-	-
Mohr et al. [266]	STR	1 × 5	30 m	25 s	A^{K}	-	SEP, S_{best}: 4.34 ± 0.05 s; S_{avg}: 4.45 ± 0.05 s; SEM, S_{best}: 4.32 ± 0.06 s; S_{avg}: 4.41 ± 0.07 s	-	-	-
Mohr et al. [267]	STR	1 × 3	30 m	25 s	A^{K}	-	S_{total}: 13.36 ± 0.11 s	-	-	-
Moncef et al. [268]	SHU	1 × 6	40 m (20 + 20)	On 20 s (~14 s)	P	-	S_{avg}: 6.38 ± 0.86 s	-	-	-
Morcillo et al. [48]	STR	1 × 12	30 m	30 s	P	-	S_{best}: 4.09 ± 0.05 s; S_{dec}: 3.7 ± 1.5%	-	-	$B[La]_{peak}$: 9.5 ± 2.3 mmol·L^{-1}
Moreira et al. [269]	STR	1 × 5	30 m	25 s	A^{Q}	-	S_{total}: 4.65 ± 0.68 s	-	-	-

Study	Exercise protocol						Outcomes			
	RST Mode	Sets × Reps	Distance / Duration	Rest Time	Rest Mode	I-set Rest	Performance	Perceptual	Neuromuscular	Physiological
Mujika et al. [164]	STR	1 × 6	30 m	On 30 s (~26 s)	A^{L}	-	U17, S_{avg}: 4.43 ± 0.11 s; S_{total}: 26.61 ± 0.53 s; S_{dec}: 4.1 ± 1.1%; U18, S_{avg}: 4.39 ± 0.12 s; S_{total}: 26.34 ± 0.94 s; S_{dec}: 4.6 ± 1.1%	-	-	U17, $B[La]_{peak}$: 10.9 ± 1.7 $mmol·L^{-1}$; U18, $B[La]_{peak}$: 12.3 ± 1.5 $mmol·L^{-1}$
Müller et al. [270]	STR	1 × 6	35 m	10 s	P	-	-	-	△ CMJ ^{AC}: 36.1 ± 5.7 to 34.4 ± 4.9 cm (-4.8%)	$B[La]_{post}$: 11.2 ± 4.4 $mmol·L^{-1}$; $B[La]^{5'}$: 15.0 ± 3.9 HR_{peak}: 174 ± 20 $b·min^{-1}$
Nakamura et al. [272]	SHU	1 × 6	30 m (15 + 15)	On 20 s (~15 s)	P	-	S_{best}: 5.62 ± 0.16 s; S_{avg}: 6.03 ± 0.18 s; S_{dec}: 7.4 ± 2.5%	-	-	$B[La]^{3'}$: 10.6 ± 2.1 $mmol·L^{-1}$; HR_{peak}: 180 ± 6 $b·min^{-1}$
Nascimento et al [273]	SHU	1 × 8	40 m (10 + 20 + 10)	20 s (~14 s)	P	-	CON, S_{best}: 8.53 ± 0.34 s; S_{avg}: 9.09 ± 0.39 s; S_{dec}: 6.5 ± 1.1%; INT, S_{best}: 8.14 ± 0.18 s; S_{avg}: 8.53 ± 0.15 s; S_{dec}: 4.8 ± 0.8%	-	-	CON, $B[La]_{peak}$: 13.2 ± 2.7 $mmol·L^{-1}$ INT, $B[La]_{peak}$: 16.2 ± 2.8 $mmol·L^{-1}$
Nedrehagen & Saeterbakken [274]	SHU	1 × 6	40 m (20 + 20)	30 s	P	-	INT, S_{avg}: 7.79 ± 0.37 s CON, S_{avg}: 7.79 ± 0.5	-	-	-
Nikolaidis et al. [275]	STR	1 × 10	20 m	On 30 s (~27 s)	A^{Q}	-	S_{best}: 3.14 ± 0.11 s; S_{avg}: 3.24 ± 0.11 s; S_{dec}: 3.4 ± 1.6%	-	-	-
Okuno et al. [271]	SHU	1 × 6	30 m (15 + 15)	On 20 s (~14 s)	P	-	S_{best}: 5.82 ± 0.15 s; S_{avg}: 6.06 ± 0.18; S_{dec}: 4.2 ± 1.1%	-	-	-
Padulo et al. [276]	SHU	1 × 6	40 m (20 + 20)	20 s	P	-	Test, S_{best}: 7.09 ± 0.18 s; S_{total}: 44.84 ± 1.09 s; S_{dec}: 5.5 ± 1.6%; Retest, S_{best}: 7.06 ± 0.15 s; S_{total}: 44.76 ± 1.09 s; S_{dec}: 5.7 ± 1.7%	Test, CR10: 7.2 ± 0.9 au; Retest, CR10: 7.2 ± 0.4 au	-	Test, $B[La]^{3'}$: 11.3 ± 2.0 $mmol·L^{-1}$; Retest, $B[La]^{3'}$: 11.7 ± 1.7 $mmol·L^{-1}$
Padulo et al. [277]	SHU	1 × 6	40 m (20 + 20)	20 s	P	-	Test, S_{best}: 6.97 ± 0.12 s; S_{total}: 43.76 ± 0.90 s; S_{dec}: 4.6 ± 1.5%; Retest, S_{best}: 7.03 ± 0.15 s; S_{total}: 44.08 ± 0.75 s; S_{dec}: 4.5 ± 1.1%	-	-	Test, $B[La]^{3'}$: 11.6 ± 2.2 $mmol·L^{-1}$; Retest, $B[La]^{3'}$: 11.6 ± 2.1 $mmol·L^{-1}$
Padulo et al. [114]	SHU	1 × 10	30 m (15 + 15)	30 s	P	-	Test, S_{best}: 5.81 ± 0.32 s; S_{total}: 60.19 ± 3.57 s; S_{dec}: 3.5 ± 1.7%; Retest, S_{best}: 5.82 ± 0.31 s; S_{total}: 60.50 ± 3.56 s; S_{dec}: 3.8 ± 1.6%;	Test, CR10: 7.8 ± 1.3 au; Retest, CR10: 8.0 ± 1.2 au	-	Test, $B[La]^{3'}$: 11.9 ± 2.5 $mmol·L^{-1}$; Retest, $B[La]^{3'}$: 11.9 ± 2.1 $mmol·L^{-1}$

Study	Exercise protocol						Outcomes			
	RST Mode	Sets × Reps	Distance / Duration	Rest Time	Rest Mode	I-set Rest	Performance	Perceptual	Neuromuscular	Physiological
	SHU	1 × 10	30 m (10 + 10 + 10)	30 s	P	-	Test, S_{best}: 7.02 ± 0.44 s; S_{total}: 72.49 ± 4.82 s; S_{dec}: 3.3 ± 1.3%; Retest, S_{best} 7.01 ± 0.44 s; S_{total}: 72.51 ± 4.77 s; S_{dec}: 3.4 ± 1.4%	Test, CR10: 7.8 ± 1.6 au Retest, CR10: 8.1 ± 1.5 au	-	Test, B[La]$^{3'}$: 11.3 ± 2.8 mmol·L^{-1} Retest, B[La]$^{3'}$: 11.4 ± 2.5 mmol·L^{-1}
Padulo et al. [156]	SHU	1 × 6	40 m (20 + 20)	20 s	P	-	Test, S_{best}: 7.10 ± 0.20 s; S_{total}: 44.89 ± 1.14 s; S_{dec}: 5.5 ± 1.9%; Retest, S_{best}: 7.09 ± 0.20; S_{total}: 44.79 ± 1.13 s; S_{dec}: 5.3 ± 1.7%	Test, CR10: 7.0 ± 1.2 au; Retest, CR10: 7.2 ± 0.7 au	-	Test, B[La]$^{3'}$: 11.2 ± 2.1 mmol·L^{-1} Retest, B[La]$^{3'}$: 11.3 ± 2.0 mmol·L^{-1}
	SHU	1 × 6	40 m (20 + 20)	20 s	A[P]	-	S_{best}: 7.16 ± 0.23; S_{total}: 45.77 ± 1.34 s; S_{dec}: 6.6 ± 1.6%	CR10: 7.9 ± 1.2 au	-	B[La]$^{3'}$: 13.1 ± 2.1 mmol·L^{-1}
Padulo et al. [150]	SHU	1 × 6	40 m (20 + 20)	15 s	P		S_{best}: 7.36 ± 0.10 s; S_{total}: 46.12 ± 0.85 s; S_{dec}: 4.5 ± 1.2%	-	△ CMJ [AA]: 39.2 cm to 35.6 ± 0.9 cm (-9.0%)	B[La]$^{3'}$: 14.5 ± 0.4 mmol·L^{-1}
	SHU	1 × 6	40 m (20 + 20)	20 s	P		S_{best}: 7.35 ± 0.16 s; S_{total}: 45.41 ± 0.94 s; S_{dec}: 3.0 ± 0.9%	-	△ CMJ [AA]: 39.2 cm to 37.5 ± 2.7 cm (-4.3%)	B[La]$^{3'}$: 12.7 ± 1.2 mmol·L^{-1}
	SHU	1 × 6	40 m (20 + 20)	25 s	P		S_{best}: 7.33 ± 0.13 s; S_{total}: 44.82 ± 0.90 s; S_{dec}: 1.9 ± 0.7%	-	△ CMJ [AA]: 39.2 cm to 38.3 ± 3.7 cm (-2.3%)	B[La]$^{3'}$: 8.0 ± 1.5 mmol·L^{-1}
Paulauskas et al. [122]	SHU	3 × 10	30 m (15 + 15)	30 s	P	5 min P	S_{best}: set 1, 58.45 ± 1.63 s; set 2, 59.25 ± 2.03 s; set 3, 60.02 ± 2.41 s;	-	-	B[La]$^{3'}$: 13.02 ± 2.28 mmol·L^{-1}; HR_{peak}: set 1, 175 ± 8 b·min^{-1}; set 2, 178 ± 5 b·min^{-1}; set 3, 182 ± 10 b·min^{-1}; HR_{avg}: set 1, 163 ± 9.1 b·min^{-1}; set 2, 169 ± 7 b·min^{-1}; set 3, 169 ± 6 b·min^{-1}
	SHU	3 × 20	15 m (7.5 + 7.5)	15 s	P	5 min P	S_{best}: set 1, 53.37 ± 1.64 s; set 2, 53.58 ± 1.48 s; set 3, 54.04 s	-	-	B[La]$^{3'}$: 8.5 ± 3.4 mmol·L$^{-1;}$ HR_{peak}: set 1, 174 ± 9 b·min^{-1}; set 2, 178 ± 8 b·min^{-1}; set 3, 179 ± 7 b·min^{-1}; HR_{avg}: set 1, 161 ± 10 b·min^{-1}; set 2, 170 ± 9 b·min^{-1}; 171 ± 8 b·min^{-1}
Perroni et al. [103]	MD[A]	1 × 7	30 m	25 s	A[K]	-	S_{avg}: 6.12 ± 0.04 s; S_{total}: 42.84 ± 1.96 s; S_{dec}: 3.7 ± 1.2%	-	-	-
Petisco et al. [278]	SHU	1 × 6	30 m (15 + 15)	20 s	P	-	S_{best}: 5.77 ± 0.15 s; S_{total}: 35.70 ± 0.65 s	-	-	-

Study	Exercise protocol						Outcomes			
	RST Mode	Sets × Reps	Distance / Duration	Rest Time	Rest Mode	I-set Rest	Performance	Perceptual	Neuromuscular	Physiological
Purkhús et al. [279]	STR	1 × 5	30 m	25 s	A^K	-	CON, S_{avg}: 5.46 ± 0.38 s<br>INT, S_{avg}: 5.64 ± 0.29 s	-	-	-
Pyne et al. [280]	STR	1 × 6	30 m	On 20 s (~16 s)	P	-	S_{total}: 25.83 ± 0.60 s;<br>S_{dec}: 3.8 ± 1.1%	-	-	-
Ramírez-Campillo et al. [281]	STR	1 × 6	35 m	10 s	P	-	CON, S_{avg}: 7.35 ± 0.50 s;<br>PLA, S_{avg}: 7.08 ± 0.60 s;<br>INT, S_{avg}: 7.48 ± 1.00 s	-	-	-
Rampinini et al. [312]	SHU	1 × 6	40 m (20 + 20)	20 s	P	-	S_{best}: 7.00 ± 0.19 s; S_{avg}: 7.25 ± 0.17 s;<br>S_{dec}: 3.3 ± 1.6%	-	-	-
Rampinini et al. [174]	SHU	1 × 6	40 m (20 + 20)	20 s	P	-	PRO, S_{best}: 6.86 ± 0.13 s; S_{avg}: 7.17 ± 0.09 s; S_{dec}: 4.5 ± 1.9%;<br>AM, S_{best}: 6.97 ± 0.15 s; S_{avg}: 7.41 ± 0.19 s; S_{dec}: 6.0 ± 1.9%	-	-	-
Rodríguez-Fernández et al. [165]	STR	1 × 8	30 m	25 s	A	-	YTH, S_{best}: 4.03 ± 0.15 s; S_{avg}: 4.19 ± 0.12 s; S_{total}: 33.52 ± 0.97 s; S_{dec}: 3.9 ± 1.6%<br>PRO, S_{best}: 3.92 ± 0.11 s; S_{avg}: 4.12 ± 0.12 s; S_{total}: 32.91 ± 0.91 s; S_{dec}: 5.2 ± 1.9%	-	-	-
Rodríguez-Fernández et al. [284]	STR	1 × 8	30 m	25 s	A^K	-	S_{best}: 3.87 ± 0.04 s; S_{avg}: 4.03 ± 0.04 s;<br>S_{total}: 32.26 ± 0.31 s; S_{dec}: 4.3 ± 0.3%	-	-	-
Rey et al. [283]	STR	1 × 6	25 m	25 s	A^K	-	INT, S_{best}: 3.21 ± 0.08 s; S_{avg}: 3.29 ± 0.07 s; S_{total}: 19.77 ± 0.46 s; S_{dec}: 2.4 ± 1.5%<br>CON, S_{best}: 3.15 ± 0.12 s; S_{avg}: 3.25 ± 0.15 s; S_{total}: 19.53 ± 0.95 s; S_{dec}: 3.1 ± 1.9%	-	-	-
Røksund et al.[285]	STR	1 × 8	30 m	On 30 s (~27 s)	P	-	S_{avg}: 3.14 ± 0.10 s	-	-	-
Ruscello et al. [286]	STR	1 × 7	30 m	$1{:}5^N$ (~26 s)	P	-	S_{avg}: 5.24 ± 0.33 s	-	-	$B[La]^{3'}$: 10.9 ± 1.8 $mmol \cdot L^{-1}$
	SHU	1 × 7	30 m (15 + 15)	$1{:}3^N$ (~21 s)	P	-	S_{avg}: 6.84 ± 0.44 s	-	-	$B[La]^{3'}$: 7.9 ± 2.4 $mmol \cdot L^{-1}$

Study	Exercise protocol						Outcomes			
	RST Mode	Sets × Reps	Distance / Duration	Rest Time	Rest Mode	I-set Rest	Performance	Perceptual	Neuromuscular	Physiological
Ruscello et al. [104]	STR	1 × 7	30 m	1:5[N] (~22 s)	P	-	S_{avg}: 4.53 ± 0.28 s; S_{dec}: 4.8%	-	△ CMJ [AD]: 46.8 ± 4.5 to 43.3 ± 5.0 cm (-7.5%)	-
	SHU	1 × 7	30 m (15 + 15)	1:5[N] (~30 s)	P	-	S_{avg}: 5.89 ± 0.35 s; S_{dec}: 3.4%	-	△ CMJ [AD]: 46.9 ± 4.5 to 43.0 ± 5.1 cm (-8.3%)	-
	MD[C]	1 × 7	30 m (5 m per turn)	1:5[N] (~42 s)	P	-	S_{avg}: 8.51 ± 0.41 s; S_{dec}: 2.5%	-	△ CMJ [AD]: 46.9 ± 4.4 to 43.5 ± 5.0 cm (-7.1%)	-
Russell et al. [123]	STR	1 × 15	30 m	60 s	P	-	CON, S_{avg}: 4.34 ± 0.17 s; S_{total}: 65.08 ± 2.56 s; INT, S_{avg}: 4.37 ± 0.23 s; S_{total}: 65.56 ± 3.38 s	-	-	CON, △ CK 24 h: 232 ± 44 u·L^{-1} to 785 ± 129 u·L^{-1} (238%); INT △ CK 24 h: 232 ± 49 u·L^{-1} to 799 ± 141 u·L^{-1} (244%)
Salleh et al. [287]	MD[C]	1 × 5	40 m	60 s	A[U]	-	S_{avg}: 7.54 ± 0.65 s; S_{dec}: 1.9 ± 1.6%	-	-	-
Sánchez-Sánchez et al. [117]	SHU	1 × 6	40 m (20 + 20)	20 s	A	-	Sys1, S_{best}: 7.38 ± 0.25 s; S_{avg}: 7.93 ± 0.30 s; S_{total}: 47.55 ± 1.74 s; Sys2, S_{best}: 7.5 ± 0.26 s; S_{avg}: 7.97 ± 0.26 s; S_{total}: 47.85 ± 1.59 s; Sys3, S_{best}: 7.74 ± 0.29 s; S_{avg}: 8.24 ± 0.29 s; S_{total}: 49.46 ± 1.75 s; Sys4, S_{best}: 7.51 ± 0.32 s; S_{avg}: 8.02 ± 0.25 s; S_{total}: 48.14 ± 1.48 s	-	Sys1, △ CMJ[AA]: 36.5 ± 4.4 to 28.3 ± 4.5cm (-22.5%); Sys2, △ CMJ[AA]: 35.5 ± 5.4 to 26.0 ± 4.9 cm (-26.1%); Sys3, △ CMJ[AA]: 36.4 ± 5.7 to 26.5 ± 5.2 cm (-27.1%); Sys4, △ CMJ[AA]: 36.9 ± 5.1 to 30.1 ± 5.9 cm (-18.5%)	Sys1, $B[La]^{1'}$: 12.9 ± 2.3 mmol·L^{-1}; $B[La]^{3'}$: 13.0 ± 2.5 mmol·L^{-1}; HR_{peak} 184 ± 13 b·min^{-1}; Sys2, $B[La]^{1'}$: 12.4 ± 2.4 mmol·L^{-1}; $B[La]^{3'}$: 13.0 ± 3.0 mmol·L^{-1}; HR_{peak} 185 ± 12 b·min^{-1}; Sys3, $B[La]^{1'}$: 11.0 ± 2.3 mmol·L^{-1}; $B[La]^{3'}$: 11.0 ± 1.9 mmol·L^{-1}; HR_{peak} 183 ± 13 b·min^{-1}; Sys4, $B[La]^{1'}$: 11.8 ± 2.5 mmol·L^{-1}; $B[La]^{3'}$: 11.1 ± 2.5 mmol·L^{-1}; HR_{peak} 185 ± 12 b·min^{-1}
Sánchez-Sánchez et al. [288]	STR	1 × 7	30 m	20 s	A	-	S_{avg}: 4.46 ± 0.17 s; S_{dec}: 4.7 ± 2.0%	-	-	-

Study	Exercise protocol						Outcomes			
	RST Mode	Sets × Reps	Distance / Duration	Rest Time	Rest Mode	I-set Rest	Performance	Perceptual	Neuromuscular	Physiological
Sánchez-Sánchez et al. [289]	STR	1 × 6	20 m	20 s	P	-	S_{best}: 3.19 ± 0.11 s; S_{avg}: 3.29 ± 0.08 s	-	-	-
Sanders et al. [290]	STR	1 × 10	30 m	25 s	P	-	-	-	-	HR_{post} (% HR_{max}): 93%
Scanlan et al. [291]	STR	1 × 10	20 m	30 s	P	-	S_{total}: 35.02 ± 2.1 s; S_{dec}: 2.7 ± 1.2%	6–20: 15.2 ± 2.1	-	$B[La]_{post}$: 4.6 ± 0.8 to 11.0 ± 1.6 mmol·L^{-1}; HR_{peak}: 169 ± 12 b·min^{-1}
	STR	1 × 10	20 m	30 s	A^{AG}	-	S_{total}: 37.73 ± 2.5 s; S_{dec}: 9.4 ± 5.2%	6–20: 18.4 ± 1.3 au	-	$B[La]_{post}$: 5.0 ± 1.1 to 16.5 ± 4.5 mmol·L^{-1}; HR_{peak}: 187 ± 9 b·min^{-1}
Scanlan et al. [292]	SHU	1 × 12	20 m ^{AE}	20 s	P	-	S_{dec}: 2.8 ± 0.8%	-	-	-
Selmi et al. [58]	STR	2 × 5	20 m	15 s	A^{J}	1 min P	S_{best}: set 1, 3.31 ± 0.14 s; set 2, 3.38 ± 0.12 s; S_{total}: set 1, 16.97 ± 0.69 s; set 2, 17.69 ± 0.58 s; S_{dec}: Set 1, 2.9 ± 1.6%; Set 2, 5.1 ± 2.8%	CR10: 6.3 ± 1.4 au	-	△ $B[La]^{3'}$: 1.8 ± 0.6 to 8.1 ± 2.2 mmol·L^{-1}<br>HR_{peak}: 186 ± 14 b·min^{-1}<br>HR_{avg}: 137 ± 12 b·min^{-1}
	STR	2 × 5	20 m	15 s	A^{J}	2 min P	S_{best}: set 1, 3.28 ± 0.10 s; set 2, 3.33 ± 0.11 s; S_{total}: set 1, 16.90 ± 0.57 s; set 2, 17.11 ± 0.47 s; S_{dec}: Set 1, 3.2 ± 1.6%; Set 2, 2.8 ± 1.6%	CR10: 3.2 ± 1.5 au	-	△ $B[La]^{3'}$: 1.5 ± 0.2 to 8.2 ± 1.0 mmol·L^{-1}<br>HR_{peak}: 182 ± 9 b·min^{-1}<br>HR_{avg}: 125 ± 11 b·min^{-1}
	STR	2 × 5	20 m	15 s	A^{J}	4 min P	S_{best}: set 1, 3.31 ± 0.11 s; set 2, 3.31 ± 0.11 s; S_{total}: set 1, 16.97 ± 0.64 s; set 2, 17.06 ± 0.55 s; S_{dec}: Set 1, 2.7 ± 1.3%; Set 2, 3.1 ± 1.4%	CR10: 3.4 ± 1.2 au	-	△ $B[La]^{3'}$: 1.6 ± 0.3 to 8.5 ± 1.8 mmol·L^{-1}<br>HR_{peak}: 180 ± 10 b·min^{-1}<br>HR_{avg}: 114 ± 5 b·min^{-1}
Selmi et al. [293]	SHU	1 × 20	40 m (20 + 20)	20 s	P	-	INT, S_{best}: 7.53 ± 0.48 s; S_{total}: 47.86 ± 2.81 s; S_{dec}: 6.0 ± 1.9%<br>CON, S_{best}: 7.69 ± 0.31 s; S_{total}: 49.05 ± 1.52 s; S_{dec}: 6.3 ± 2.0%	-	-	-
Shalfawi et al. [294]	STR	1 × 7	30 m	30 s	P	-	S_{best}: 4.93 ± 0.20 s; S_{avg}: 5.04 ± 0.20 s; S_{total}: 35.35 ± 1.40 s; S_{dec}: 2.2 ± 1.0%	-	-	-
Shalfawi et al. [295]	STR	1 × 10	40 m	60 s	P	-	INT, S_{avg}: 5.92 ± 0.26 s<br>CON, S_{avg}: 5.84 ± 0.27 s	-	-	-
Shalfawi et al. [296]	STR	1 × 10	40 m	60 s	P	-	ATG, S_{avg}: 6.15 ± 0.4 s<br>RS, S_{avg}: 6.19 ± 0.25 s	-	-	-

Study	Exercise protocol						Outcomes			
	RST Mode	Sets × Reps	Distance / Duration	Rest Time	Rest Mode	I-set Rest	Performance	Perceptual	Neuromuscular	Physiological
Silva et al. [297]	SHU	1 × 6	40 m (20 + 20)	20 s	P	-	S_{best}: 6.44 ± 0.14 s; S_{avg}: 6.57 ± 0.26 s; S_{total}: 44.20 ± 0.40 s; S_{dec}: 9.8 ± 1.4%	-	-	-
Soares-Caldeira et al. [298]	SHU	1 × 6	40 m (20 + 20)	20 s	P	-	INT, S_{best}: 7.17 ± 0.37 s; S_{avg}: 7.62 ± 0.35 s; S_{dec}: 6.3 ± 2.0%; CON, S_{best}: 6.95 ± 0.16 s; S_{avg}: 7.49 ± 0.20 s; S_{dec}: 7.8 ± 4.3%	-	-	-
Spineti et al. [299]	SHU	1 × 6	40 m (20 + 20)	20 s	P	-	CCT, S_{best}: 6.93 ± 0.15 s; S_{avg}: 7.43 ± 0.10; S_{dec}: 7.2 ± 2.2% TST, S_{best}: 7.11 ± 0.19 s; S_{avg}: 7.54 ± 0.23; S_{dec}: 6.1 ± 1.9%	-	-	-
Suarez-Arrones et al. [105]	MD[AF]	1 × 6	40 m (20 + 20)	20 s	P	-	RS, S_{best}: 7.60 ± 0.20 s; S_{avg}: 8.00 ± 0.20 s; S_{dec}: 5.3 ± 1.3%; SQ, S_{best}: 7.50 ± 0.30 s; S_{avg}: 7.90 ± 0.30 s: S_{dec}: 5.0 ± 2.0%	-	-	-
	SHU	3 × 6	40 m (20 + 20)	20 s	P	4 min P	-	6–20: 13.9 ± 0.4 au	-	-
Stojanovic et al. [300]	SHU	1 × 10	30 m (15 + 15)	30 s	P	-	S_{avg}: 5.77 ± 0.18 s; S_{dec}: 3.5 ± 1.1%	-	-	-
Taylor et al. [2]	STR	3–4 × 7	30 m	20 s	P	4 min P	-	-	-	HR_{peak} (% HRmax): 92 ± 5%
	SHU	3–4 × 7	30 m	20 s	P	4 min P	-	-	-	HR_{peak} (% HRmax): 89 ± 11%
Teixeira et al. [301]	STR	1 × 8	40 m	20 s	P	-	$IT_{7.5}$, S_{best}: 8.86 ± 0.25 s; S_{avg}: 9.39 ± 0.26 s; S_{dec}: 6.5 ± 1.4%; IT_{15}, S_{best}: 8.83 ± 0.36 s; S_{avg}: 9.33 ± 0.36 s; S_{dec}: 5.7 ± 3.2%	-	-	-
Thomassen et al. [302]	STR	1 × 10	20 m	15 s	A[K]	-	INT, S_{avg}: 3.35 ± 0.07 s; S_{total}: 33.44 ± 0.44 s; S_{dec}: 5.8 ± 1.0% NT, S_{avg}: 3.34 ± 0.09 s; S_{total}: 33.41 ± 0.32 s; S_{dec}: 5.9 ± 0.8%	-	-	-
Tønnessen et al. [303]	STR	1 × 10	40 m	60 s	-	P	INT, Savg: 5.42 ± 0.18 s; CON, S_{avg}: 5.41 ± 0.19 s	-	-	-

Study	Exercise protocol						Outcomes			
	RST Mode	Sets × Reps	Distance / Duration	Rest Time	Rest Mode	I-set Rest	Performance	Perceptual	Neuromuscular	Physiological
Torreblanca-Martinez et al. [304]	STR	1 × 12	30 m	30 s	P	-	S_{dec}: 6.5 ± 3.0%	6–20: 15.2 ± 2.5 au	-	HR_{post}: 179 ± 12 b·min^{-1}
Tounsi et al. [176]	SHU	1 × 6	40 m (20 + 20)	20 s	P	-	M, S_{best}: 7.09 ± 0.24 s; S_{avg}: 7.32 ± 0.28; S_{dec}: 3.2 ± 1.2%<br>F, S_{best}: 8.42 ± 0.47 s; S_{avg}: 8.85 ± 0.45; S_{dec}: 5.1 ± 2.5%	-	-	-
Trecroci et al. [305]	STR	1 × 5	30 m	25 s	P	-	SST, S_{best}: 4.26 ± 0.11 s; S_{total}: 21.94 ± 0.67 s; ARC, S_{best}: 4.25 ± 0.07 s; S_{total}: 21.91 ± 0.58 s	-	-	-
Turki et al. [111]	MD^{X}	1 × 6	20 m (4 m per turn)	25 s	A^{K}	-	PRO, S_{best}: 5.39 ± 0.18 s; S_{avg}: 5.52 ± 0.17 s; S_{total}: 33.09 ± 1.00 s; S_{dec}: 2.4 ± 1.0%; COL, S_{best}: 5.49 ± 0.26 s; S_{avg}: 5.62 ± 0.27 s; S_{total}: 33.70 ± 1.60 s; S_{dec}: 2.4 ± 0.6%	-	-	-
Ulupinar et al. [126]	STR	1 × 10	40 m	30 s	P	-	S_{best}: 5.43 ± 0.03 s; S_{total}: 56.7 ± 1.6 s; S_{dec}: 4.8 ± 1.7%	6–20: 17 ± 1 au	-	B[La]$_{peak}$: 18.6 ± 1.7 mmol·L^{-1}; HR_{peak}: 184 ± 8 b·min^{-1} HR_{avg}: 164 ± 7 b·min^{-1}
	STR	1 × 20	20 m	15 s	P	-	S_{best}: 3.18 ± 0.03 s; S_{total}: 67.3 ± 3.0 s; S_{dec}: 6.9 ± 2.8%	6–20: 19 ± 1 au	-	B[La]$_{peak}$: 16.6 ± 2.2 mmol·L^{-1}: HR_{peak}: 188 ± 8 b·min^{-1} HR_{avg}: 168 ± 9 b·min^{-1}
Ulupinar et al. [125]	STR	1 × 20	15 m	30 s	P	-	S_{total}: 49.9 ± 1.2; S_{dec}: 3.6 ± 1.8%	6–20: 11.5 ± 2.9 au	-	B[La]$_{peak}$: 9.1 ± 3.0 mmol·L^{-1}: HR_{peak}: 186 ± 9 b·min^{-1} HR_{avg}: 168 ± 9 b·min^{-1}
	STR	1 × 20	15 m	1:5^{N} (~12 s)	P	-	S_{total}: 52.7 ± 1.3; S_{dec}: 8.7 ± 2.8%	6–20: 16.3 ± 1.9 au	-	B[La]$_{peak}$: 14.9 ± 3.7 mmol·L^{-1}: HR_{peak}: 190 ± 11 b·min^{-1} HR_{avg} : 178 ± 11 b·min^{-1}
	STR	1 × 10	30 m	30 s	P	-	S_{total}: 44.9 ± 1.2; S_{dec}: 7.1 ± 3.8%	6–20: 13.9 ± 2.4 au	-	B[La]$_{peak}$: 15.0 ± 4.1 mmol·L^{-1}: HR_{peak}: 191 ± 13 b·min^{-1} HR_{avg}: 172 ± 10 b·min^{-1}
	STR	1 × 10	30 m	1:5^{N} (~22 s)	P	-	S_{total}: 45.8 ± 1.1; S_{dec}: 9.3 ± 2.3%	6–20: 15.8 ± 2.9 au	-	B[La]$_{peak}$: 16.9 ± 3.5 mmol·L^{-1}: HR_{peak}: 190 ± 12 b·min^{-1} HR_{avg}: 177 ± 8 b·min^{-1}
Van den Tillaar et al. [306]	STR	1 × 7	30 m	On 30 s (~25 s)	A^{K}	-	S_{avg}: 5.46 ± 0.33 s	-	-	-

Study	Exercise protocol						Outcomes			
	RST Mode	Sets × Reps	Distance / Duration	Rest Time	Rest Mode	I-set Rest	Performance	Perceptual	Neuromuscular	Physiological
Vasquez-Bonilla et al. [307]	STR	1 × 8	20 m	20 s	A[K]	-	S_{best}: 3.81 ± 0.17 s; S_{avg}: 4.08 ± 0.21 s; S_{total}: 32.64 ± 1.75 s; S_{dec}: 7 ± 3%	-	-	-
Wadley & Le Rossignol [308]	STR	1 × 12	20 m	20 s	P	-	S_{total}: 39.31 ± 0.12 s; S_{dec}: 5.5 ± 3.3%	-	-	-
West et al. [309]	SHU	1 × 6	40 m (20 + 20)	20 s	P	-	S_{best}: 6.60 ± 0.16 s; S_{avg}: 6.87 ± 0.15 s; S_{total}: 41.23 ± 0.92 s	-	-	-
Woolley et al. [33]	STR	1 × 40	15m	30s	P[R]	-	-	6–20: 16.7 ± 1.8 au	-	△ CK 24 h: 279 ± 322 to 1121 ± 1362 u·L^{-1} (302%)
Yanci et al. [310]	STR	1 × 6	30 m	25 s	A	-	CON, S_{avg}: 4.57 ± 0.20 s<br>PLY1: S_{avg}: 4.47 ± 0.22 s<br>PLY2: S_{avg}: 4.45 ± 0.23 s	-	-	-
Zagatto et al. [106]	SHU	1 × 10	30m	30 s	P	-	S_{best}: 6.56 ± 0.30 s; S_{avg}: 6.84 ± 0.30 s; S_{total}: 68.40 ± 2.91 s; S_{dec}: 4.2 ± 1.8%	-	-	$B[La]_{peak}$: 9.8 ± 2.5 mmol·L^{-1}; VO_{2avg}: 37.0 ± 2.9 ml·min^{-1}·kg^{-1}; HR_{peak}: 185 ± 9 b·min^{-1}
	MD[C]	1 × 10	30 m (5 m per turn)	30 s	P	-	S_{best}: 8.14 ± 0.36 s; S_{avg}: 8.39 ± 0.36 s; S_{total}: 83.99 ± 3.60 s; S_{dec}: 3.0 ± 1.1%	-	-	$B[La]_{peak}$: 8.2 ± 1.9 mmol·L^{-1}; VO_{2avg}: 36.1 ± 3.2 ml·min^{-1}·kg^{-1}; HR_{peak}: 186 ± 9 b·min^{-1}
Zagatto et al. [311]	SHU	2 × 10	30 m (10 + 10 + 10)	30 s	P	P 5.50 min	Set 1, S_{best}: 6.85 ± 0.35 s; S_{avg}: 7.01 ± 0.31 s; S_{total}: 70.15 ± 3.07 s; S_{dec}: 2.4 ± 1.5%<br>Set 2, S_{best}: 6.88 ± 0.32 s; S_{avg}: 7.13 ± 0.36 s; S_{total}: 71.31 ± 3.59 s; S_{dec}: 3.6 ± 1.58%	-	△ CMJ [AB]: 43.2 ± 9.7 to 37.6 ± 4.0 cm (-9.4 ± 18.0%)	-
Zagatto et al. [107]	MD[C]	1 × 10	30 m	30 s	P	-	S_{best}: 7.09 ± 0.57 s; S_{avg}: 7.30 ± 0.63 s; S_{total}: 72.84 ± 6.42 s	-	-	-

Data are presented as mean ± SD.

Abbreviations: I-set = inter-set; RST = repeated-sprint training; sRPE = session ratings of perceived exertion; au = arbitrary units; CR10 = category ration 0–10 rating of perceived exertion scale; 6–20 = 6–20 rating of perceived exertion scale; SHU = shuttle repeated-sprint; STR = straight-line repeated-sprint; MD = multi-directional repeated-sprint; A = active recovery; P = passive recovery; M = male; F = female; $B[La]_{post}$ = blood lactate measured immediately post-exercise; $B[La]_{peak}$ = highest blood lactate value measured from two or more time-points between 0–10 min post-exercise; $B[La]^{1'}$ = blood lactate measured 1 minutes post-exercise; $B[La]^{2'}$ = blood lactate measured 2 minutes post-exercise; $B[La]^{3'}$ = blood lactate measured 3 minutes post-exercise; $B[La]^{4'}$ = blood lactate measured 4 minutes post exercise; $B[La]^{5'}$ = blood lactate measured 5 minutes post-exercise; CK 24 h = serum creatine kinase measured 24 hours post-exercise S_{dec} = percentage sprint decrement; S_{avg} = average sprint time; S_{best} = best sprint time; S_{total} = total sprint time; CMJ = counter movement jump height; HR_{avg} = average heart rate; HR_{peak} = peak heart rate; HR_{post} = end-set heart rate recorded immediately post-exercise; % HRmax = percentage of maximal heart rate; VO_{2avg} = average oxygen consumption; % VO_{2max} = percentage of maximal oxygen consumption; V_0 = theoretical maximal velocity F_0 = theoretical maximal force; P_0 = theoretical maximal power; RF_{peak} = maximal ratio of force; D_{RF} = slope/rate of decrease in ratio of force with increasing velocity; K_{vert} vertical stiffness;; K_{leg} = leg stiffness; ΔL = leg compression; Δz = centre of mass vertical displacement; $F_z$max = maximal vertical force; PLA = placebo group = CON = control group; STR-G = straight-line repeated-sprints groups; SHU-G = shuttle repeated-sprints group; High = high VO_2 max group; Med = medium VO_2 max group; Low = low VO_2 max group; INT = intervention group; U17 = under 17 players; U18 = under 18 players; U19 = under 19 players;

U20 = under 20 players; PRE = pre-season; ELY = early/start of season; MID = mid-season; END = end/post of season; YTH = youth players; SEN = senior players; PRO = professional players; SEMI = semi-proffessional players; COL = college players; REP = representative players; Club = club players; AM = amateur players; EL = elite players; S-EL = sub-elite players; M-PRO = mid-proffessional players; EXP = experienced players; FSH = freshman players; FUT = futsal players; SOC = soccer players; SAN = sand training group; GRA = grass training group; NOR = normoxia group; HYP = hypoxia group; MG = Melaneysian group; N-MG = non-Meleynesian group; ARC = active recovery condition; SSG = small sided games group; SEM = speed endurance maintenance group; SEP = speed endurance production group; RS15 = repeated-sprint group with 15 s rest; RS30 = repeated-sprint group with 30 s rest; Sys1 = turf system 1; Sys2 = turf system 2; Sys3 = turf system 3; Sys4 = turf system 4; $IT_{7.5}$ = interval training 7.5 seconds group; IT_{15} = interval training 15 seconds group; RS = repeated sprint group; ATG = agility training group; 1TR = under 17 group born 1st tertile; 2TR = under 17 group born 2nd tertile; 3TR = under 17 group born 3rd tertile; Sham = sham group; RES = resisted sprint training group; PLY = plyometric group; PLY1 = plyometrics one day per week group; PLY2 plyometrics two days per week group; LLTL = live low-train low group; IT_{100} = interval training at 100% group; IT_{86} = interval training at 86% group; SQ = squat group; TG = take-off group; PAS = passive recovery group; COL = cold water recovery group; CWT = contrast water therapy group; NT = Non-training group; ST = starting players; N-ST = non-starting players; N-SEL = non-selected players; SST = soccer specific training condition; △ = change from baseline; - = not applicable.

[A] 3 × multi-angle turns

[B] 4 × multi-angle turns

[C] 5 × multi-angle turns

[D] 2 × 45° turns

[E] 2 × 90° turns

[F] 2 × 135° turns

[G] 4 × 45° turns

[H] Run at 8 $km \cdot h^{-1}$ back to one way start line

[I] Light stretching

[J] 10 m deceleration zone + 10m run zone at either end

[K] Jog back to one way start line

[L] Jogging at 2–2.1 $m \cdot s^{-1}$

[M] Single counter-movement jump following each sprint

[N] Exercise to rest ratio

[O] Walking or running to maintain 60-65% of HR maximum

[P] 3 × counter-movement jumps following each sprint

[Q] Self-paced jogging

[R] Short enforced deceleration zone (<10m)

[S] Run at 6 $km \cdot h^{-1}$

[T] 4 × 90° turns (quadrangle)

[U] Walk for 40 s, stationary rest for 20 s

[V] 4 × 100° turns

[W] 10m zone at both ends to decelerate, then jog back to two-way start line.

[X] Run at 20% maximal aerobic speed

[Y] Run at 35% maximal aerobic speed

[Z] Run at 50% maximal aerobic speed

[AA] Measured via an Optojump

[AB] Measured via force-platforms

[AC] Measured via a contact mat

[AD] Measured via FreePower Jump

[AE] Repeated 5-0-5 Agility test: total rep distance = 20 m, timed distance = 10 m

[AF] Change of direction performed around a cone

[AG] Run at 50% maximal speed

Supplementary Table S4. Influence of programming variables on the variance of meta-analysed acute physiological, perceptual and performance demands of repeated-sprint training in team sport athletes.

		Total Variance (σ^2)		Variance Explained by Moderators (R_{2META})
		**Observed (no moderators)**	**With Moderators**	
**HR_{avg}**	b·min^{-1}	335	-	-
	% HR_{max}	19	-	-
**HR_{peak}**	b·min^{-1}	59	55	0.07
**VO_{2avg}**	ml·kg^{-1}·min^{-1}	89.6	-	-
**B[La]**	mmol·L^{-1}	9.3	6.3	0.32
**sRPE**	au (deciMax)	3.1	3.0	0.03
**S_{best}**	s	2.71	1.10	0.60
**S_{avg}**	s	2.68	0.69	0.74
**S_{dec}**	%	4.8	3.5	0.27

Dashed lines indicate outcome measure where moderator analysis could not be performed.
